# Genome Mining from Agriculturally Relevant Fungi Led to a d-Glucose Esterified Polyketide with a Terpene-like Core Structure

**DOI:** 10.1021/jacs.3c10179

**Published:** 2023-11-10

**Authors:** Chunsheng Yan, Wenyu Han, Qingyang Zhou, Kanji Niwa, Melody J. Tang, Jessica E. Burch, Yalong Zhang, David A. Delgadillo, Zuodong Sun, Zhongshou Wu, Steven E. Jacobsen, Hosea Nelson, K. N. Houk, Yi Tang

**Affiliations:** ^†^Department of Chemical and Biomolecular Engineering, ^‡^Department of Chemistry and Biochemistry, ^§^Department of Molecular, Cell, and Developmental Biology, and ^∥^Howard Hughes Medical Institute, University of California, Los Angeles, California 90095, United States; ^⊥^Division of Chemistry and Chemical Engineering, California Institute of Technology, Pasadena, California 91125, United States

## Abstract

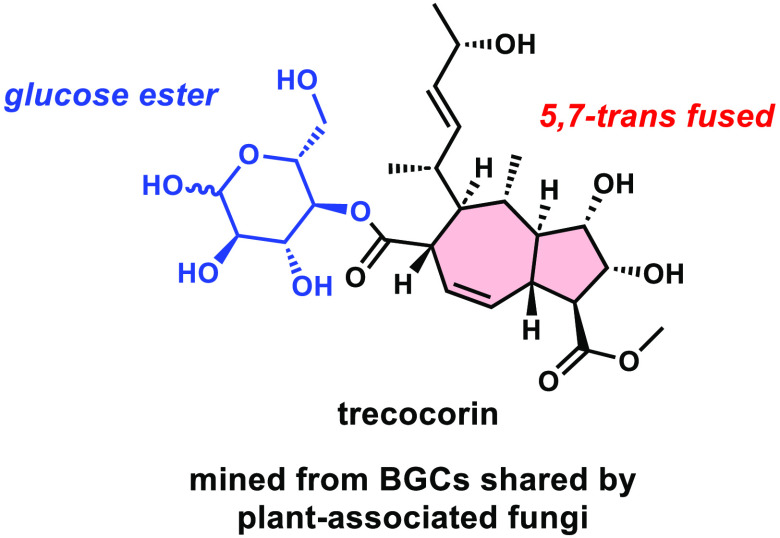

Comparison of biosynthetic gene clusters (BGCs) found in devastating plant pathogens and biocontrol fungi revealed an uncharacterized and conserved polyketide BGC. Genome mining identified the associated metabolite to be treconorin, which has a terpene-like, *trans-*fused 5,7-bicyclic core that is proposed to derive from a (4 + 3) cycloaddition. The core is esterified with d-glucose, which derives from the glycosidic cleavage of a trehalose ester precursor. This glycomodification strategy is different from the commonly observed glycosylation of natural products.

Phytopathogenic fungi that infect cereal grain crops can cause devastating loss to crop yield.^1,2^ Secondary metabolites (SMs) or natural products (NPs) biosynthesized by plant-associated fungi play important roles in colonization and pathogenesis.^3^ Numerous SMs are mycotoxins that adversely affect human health.^4−6^ Therefore, obtaining a complete inventory of SMs produced by plant-associated fungi is important. Genome sequencing revealed that these fungi encode a much large4 number of SM biosynthetic gene clusters (BGCs) than the number of identified SMs.^7^ Furthermore, the products of most BGCs cannot be predicted due to insufficient biosynthetic knowledge. As a result, the chemical space and biological activities of SMs from agriculturally important fungi are underexplored. Genome mining, which entails the activation or reconstitution of uncharacterized BGCs, has emerged as a powerful tool in establishing the inventory of SMs from microorganisms.^8,9^

Here we aim to identify new SMs from conserved BGCs among phytopathogenic fungi. Conservation of a BGC indicates a likely common role of the SM in plant–fungi interactions.^10,11^ We compared the annotated BGCs from *Bipolaris sorokiniana* and *Zymoseptoria tritici*, both of which are causal agents of wheat disease;^12,13^*Cercospora zeae-maydis*, which causes destructive foliar diseases of maize; and *Fulvia fulva*, which is the cause of leaf mold on tomato.^14,15^ Specifically, we focused on BGCs that are anchored by highly reducing polyketide synthases (HRPKSs), since notable mycotoxins and virulent factors such as fumonisins,^4^ AF-toxins,^5^ and T-toxins^6^ are biosynthesized by HRPKSs (Figure 1). Interestingly, we found that one BGC is well-conserved in all four fungi as well as a number of *Trichoderma* species, including the beneficial biocontrol fungus *Trichoderma afroharzianum* t-22 (ThT22) (Figures 2A and S1).^16^ These BGCs encode a set of five homologous enzymes (Table S10 and Figure S1), including an HRPKS, an α/β hydrolase (ABH), a P450 monooxygenase (P450), an α-glucosidase, and a protein predicted to be a terpene cyclase (TC) but having sequence homology to epoxide hydrolases (EHs).^17^ In the *Trichoderma* BGCs, such as the *tre* BGCs from ThT22, two additional conserved genes encode a P450 and an *O-*methyltransferase (*O-*MeT) (Figures 2A and S1). The combination of predicted tailoring enzymes such as α-glucosidase and EH in an HRPKS-anchored BGC suggests that the produced SM may be structurally distinct. Given that ThT22 was available in house and the pathogenic fungi are logistically difficult to work with, we targeted the *tre* BGC for heterologous expression to identify the associated SM.

**Figure 1:**
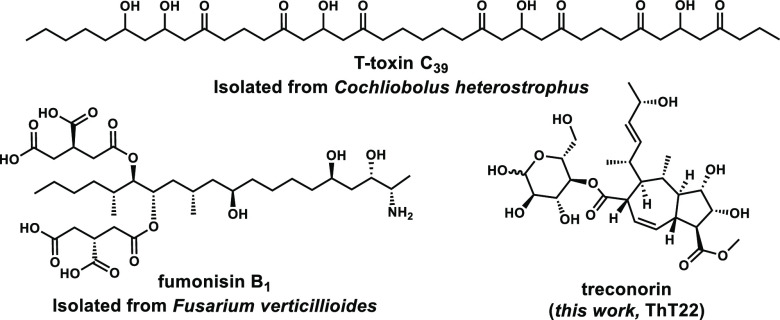
Examples of SMs biosynthesized from fungal BGCs anchored by HPRKSs. Treconorin was discovered in this work.

**Figure 2:**
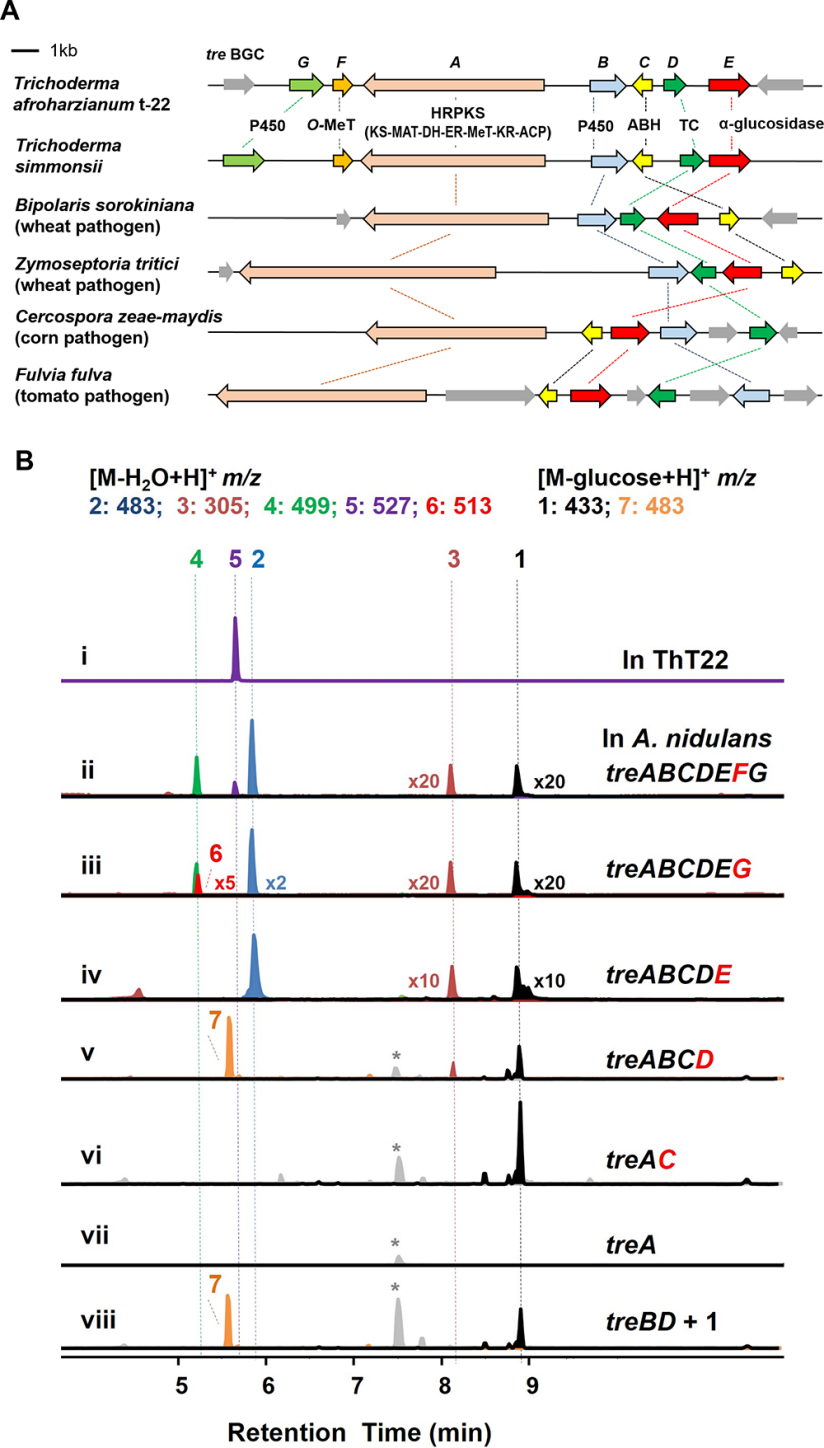
Bioinformatic analysis and reconstitution of *tre* BGC. (A) Comparison of the *tre* BGC in ThT22 to homologous BGCs in four major plant pathogens. (B) Metabolic analysis of heterologous reconstitution of the *tre* BGC in *A. nidulans*, except trace i, which is from ThT22 to indicate the presence of **5**. Selected ion monitoring traces at the indicated *m*/*z* values are shown. The multipliers indicated are used to magnify certain peaks. * indicates peaks (*m*/*z*+ 499) that are present in the control and are not related to the expression of *tre* BGC.

Upon expressing the seven *tre* genes in *Aspergillus nidulans* A1145 ΔEMΔST,^18^ five new compounds (**1**–**5**) were produced (Figure 2B, ii). When we searched the extract of the ThT22 strain cultured on CD media, only **5** (MWT: 544) could be found (Figure 2B, i). This indicates that **5** is likely the *bona fide* SM of the *tre* BGC in ThT22.^19^ Purification and NMR characterization of **5** (treconorin) from *A. nidulans* (∼1 mg/L) led to elucidation of the structure as an anomeric pair of 4′-glucosyl esters, as shown in Figure 3 (Figures S37–S42 and Table S7). **5** contains a *trans-*fused 5,7-bicyclic hydrocarbon core that is typically observed in guaiane-type sesquiterpenes.^20^ The three-dimensional structural features were established by different methods using **5** and related compounds (*vida infra*), including (1) the stereochemistry of substituents on the 5,7-ring system by NOE measurements (Figure S42); (2) the *S* configuration of C-15–OH through Mosher derivatization of **7** (Figures S2 and S49–S64 and Table S9);^21^ (3) the configuration of C-12 methyl relative to the 5,7-ring system through microcrystalline electron diffraction (MicroED) of **3** (Figure S3); (4) the absolute stereochemistry through electronic circular dichroism (ECD) calculation of **3** (Figure S4); and (5) identification of d-glucose as the sugar esterified to C-1 carboxylate (Figure S5).

**Figure 3:**
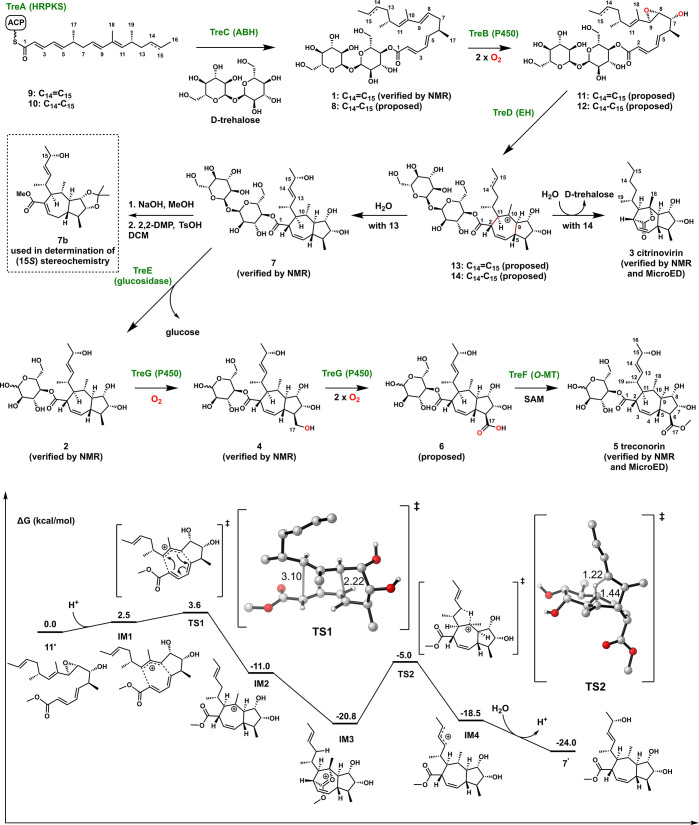
(Top) Proposed biosynthetic pathway of compounds **5** and **3**. (Bottom) Computed energy profile for the proposed mechanism from compound **11′** to compound **7′**. For the 3D structures of **TS1** and **TS2**, unimportant hydrogens have been omitted for clarity, and bond lengths for partially formed bonds are given in Å. The trehalose is replaced by methyl to simplify the computation. Computational method: ωB97X-D/def2-QZVPP/SMD(water)//ωB97X-D/def2-SVP/IEEPCM(water) under standard state (298 K, 1 atm, 1 M).

To investigate the biosynthetic pathway that affords these structural features in **5**, we performed bottom-up reconstitution of the *tre* genes in *A. nidulans* (Figures 2B and S6). While the expression of TreA alone did not lead to production of any new metabolites (Figure 2B, vii), coexpression of TreA with the ABH TreC led to accumulation of **1** with a titer of ∼40 mg/L. NMR characterization of **1** (Table S3 and Figures S13–S18) showed that it contains an acyclic polyketide esterified to trehalose through one of the C-4′–OH groups. While a number of trehalose lipids are known (Figure S7), the only examples of fungal trehalose lipids are fusaroside and emmyguyacins.^22−25^ The polyketide portion of **1**, which is **9**, is therefore synthesized by TreA, while TreC catalyzes the release of ACP-bound **9** with trehalose C-4′–OH to give **1**. Enzyme assays using TreA expressed from yeast and TreC from *Escherichia coli* were performed in the presence of malonyl-CoA, NADPH, *S-*adenosylmethionine (SAM), and d-trehalose,^26^ and **1** was only produced in the presence of all substrates and both enzymes (Figure S8). Replacement of the trehalose nucleophile with other mono- and disaccharides abolished the formation of product. Such a product release mechanism using free trehalose was observed with the TE-domain in PKS13 from *Mycobacterium tuberculosis* during the biosynthesis of 5′-trehalose monomycolate (TMM). TMM is the precursor to mycolipids that are integral in the membrane of mycobacteria.^25^

Cyclization of the polyketide portion of **1** into the 5,7-ring system in **5** requires the formation of C-2/C-11 and C-5/C-9 bonds. We proposed that the mechanism may involve carbocation intermediates typically seen in terpene cyclization but not during polyketide maturation. The final step should be quenching of the allylic C-15 carbocation with water. The required ionization of **1** may be accomplished through oxidation or epoxidation catalyzed by the conserved P450 (TreB) in the BGCs, while the EH homologue (TreD) may promote regio- and stereoselective cyclization. TreD was initially annotated as a TC but displays ∼21% sequence homology to AurD involved in epoxide-mediated polyether formation in aurovertin biosynthesis (Figure S9).^15^ While individual coexpression of TreB or TreD with TreAC did not lead to new products (Figure S6), coexpression of TreABCD led to the isolation of the new compound **7** (∼15 mg/L) (Figure 2B, v). **7** was characterized to have the same *trans-*fused 5,7-ring system as **5** but contains a C-1 trehalose ester and a C-17 methyl group (Figures S43–S48 and Table S8). To verify that **1** is the precursor to **7**, biotransformations of **1** using both *Saccharomyces cerevisiae* and *A. nidulans* expressing TreB and TreD were performed. In both hosts, formation of **7** can be observed (Figure 2B, viii and S6). *In vitro* reconstitutions of TreB and TreD were not successful, as both enzymes are membrane-bound (Figure S10). The two enzymes may form a complex to catalyze the ionization–cyclization cascade, which may rationalize why the expression of TreB alone did not lead to any oxidized intermediates.

Isolation of a minor metabolite **3**, produced by strains that coexpress TreABCD (Figure 2B, ii–v), offered clues into a potential cyclization mechanism. **3** was characterized to be citrinovirin, which was previously isolated from *Trichoderma citrinoviride* (Figures S25–S30 and S4 and Table S5) and inhibited the growth of *Staphylococcus aureus* and *Artemia salina*.^27^**3** also features the *trans-*5,7-bicyclic ring system, with a transannular lactone formed between C-10 and C-1. In addition, **3** does not contain the olefin between C-13 and C-14 and is not hydroxylated at C-15. The relative stereochemistry of **3** was unambiguously established with MicroED, while the absolute stereochemistry was confirmed by ECD (Figures S3 and S4). Because of the guaiane-like ring system, **3** was initially proposed to be synthesized from a terpene pathway.^27^ Biotransformation assays using **1** do not lead to **3** (Figure 2B, viii), suggesting that **3** is a shunt product of the pathway. We propose that **3** is derived from an over-reduced polyketide precursor **10** in which the ER domain performs an enoyl reduction in the first chain elongation cycle. Such programming “mistakes” by HRPKSs have been observed in other pathways.^28,29^ This precursor is then proposed to be released from TreA by TreC to give the trehalose ester **8** and by TreB and TreD to give **3**.

We propose that formation of the *trans-*fused 5,7-ring in **5** and **3** involves a (4 + 3) cycloaddition step to generate the key carbocation intermediates **13** and **14**, respectively (Figure 3). DFT calculations of key steps were performed to support the proposed mechanism (Figures 3 and S12). The P450 TreB is proposed to catalyze hydroxylation at C-7 as well as epoxidation of the C-8/C-9 olefin in **1** and **8** to afford **11** and **12**, respectively. As predicted by computation, the model substrate **11′** (C-1 methyl ester instead of trehalose ester) undergoes protonation to give the carbocation **IM1**, which has an allyl cation weakly associated with the diene. A nearly barrierless **TS1** (Δ*G*^⧧^ = 1.1 kcal/mol) can be formed en route to the (4 + 3) cycloadduct **IM2**. The *exo* cycloaddition with the s-*cis* diene is the most favorable and occurs in an asynchronous, concerted step in which the C-5/C-9 bond forms first, followed by the C-2/C-11 bond (Figure S12). **IM2** corresponds to the proposed biosynthetic intermediate **13**. Interestingly, **IM2** is proposed to undergo the formation of oxocarbenium **IM3** upon quenching of the C-10 carbocation with the C-1 ester oxygen. It can be readily envisaged that in the absence of additional carbocation rearrangements, such as in the case of **14**, the oxocarbenium intermediate can be attacked by water to expel trehalose and afford **3**. However, in the case of **IM3**, in which the C-14/C-15 olefin is present (as in **13**), computation predicts a facile 1,4-hydride shift from C-13 to C-10 via **TS2** with a barrier of 15.8 kcal/mol to give **IM4**. Note that the predicted stereochemistry at C-10 following the hydride shift is consistent with that confirmed in **7**. Finally, quenching of the C-15 carbocation in **IM4** by water affords **7′** (and **7**). The proposed (4 + 3) cycloaddition, while unprecedented in polyketide biosynthesis, is frequently used in synthetic chemistry to construct seven-membered rings.^30−32^ A (4 + 3) cycloaddition mechanism was recently proposed by Dickschat and co-workers in the cyclization of sodorifen from a methylated sesquiterpene precursor.^33^

To complete the biosynthetic pathway from **7** to **5**, we coexpressed TreE (α-glucosidase) with TreA–D, which led to the isolation of **2** at ∼4 mg/L (Figure 2B, iv) (Figures S19–S24 and Table S4). Compared to **7**, **2** is the glucose ester instead of the trehalose ester, consistent with the predicted function of an α-glucosidase in hydrolysis of the 1,1-glucosidic bond in **7**.^34^ The function of TreE was confirmed with the enzyme purified from *E. coli* (Figure S11). With **7** as a substrate, the addition of TreE readily led to **2** in the presence of Mg^2+^. The cassette of *treA*–*E* is conserved among the BGCs shown in Figure 2A, suggesting that **2** may be a shared intermediate or product of these pathways. The chemical logic to form the glucosyl ester in **2** is intriguing, as the pathway involves first esterification with trehalose followed by glycosidic hydrolysis to reveal the glucose ester. This strategy likely results from the high abundance of free trehalose in fungal cells,^35^ whereas free d-glucose is readily phosphorylated to glucose-6-phosphate.^36^ The d-glucosyl ester is a unique feature of **5**, as nearly all glucosylation of natural products occurs through the anomeric C-1′–OH by the action of glucosyltransferases.^37^ The combination of HRPKS, ABH, and a sugar-modifying enzyme in a BGC can be mined for new natural products that are esterified instead of glycosylated with a sugar moiety.

The two remaining enzymes TreG (P450) and TreF (*O-*MeT) are responsible for the conversion of **2** to **5** (Figure 2B, ii and iii). Coexpression of TreG with TreA and TreE led to the emergence of **4** and **6**. **4** was isolated (∼3 mg/L) and characterized to be the C-17–OH product (Figures S31–S36 and Table S6). Although **6** was not isolated due to low abundance, HRMS suggests that **6** is the C-17 carboxylate following iterative oxidation by TreG (Figure S6B). Lastly, methylation of **6** by TreF completes the biosynthesis of **5**.

Although no antimicrobial or herbicidal activities of treconorin were detected using standard assays, our biosynthetic analysis revealed new chemical logic in the formation of terpene-like scaffolds from a polyketide precursor as well as the unexpected glucose esterification.

## Supporting Information Available

Experimental details, spectroscopic data, computational details, and coordinates and energies of the calculated structures (PDF)
