# Supplementary material for: Genome Mining from Agriculturally Relevant Fungi Led to a d-Glucose Esterified Polyketide with a Terpene-like Core Structure
Source: J Am Chem Soc. 2023 Nov 10;145(46):25080–5. doi: 10.1021/jacs.3c10179 (PMC10682982; doi:10.1021/jacs.3c10179)
Supplement: Supplementary file 1 — ja3c10179_si_001.pdf [file ja3c10179_si_001.pdf]

Support Information

Genome Mining from Agriculturally Relevant Fungi Led to a D-Glucose Esterified Polyketide with a Terpene-like Core Structure

Chunsheng Yan[a], Wenyu Han[b], Qingyang Zhou[b], Kanji Niwa[a], Melody J. Tang[e], Jessica E. Burch[e], Yalong Zhang[a], David A. Delgadillo[e], Zuodong Sun[a], Zhongshou Wu[c,d], Steven E. Jacobsen[c,d], Hosea Nelson[e], K. N. Houk[a,b], Yi Tang[a,b] *

[a]Department of Chemical and Biomolecular Engineering, [b] Department of Chemistry and Biochemistry, [c]Department of Molecular, Cell, and Developmental Biology, [d]Howard Hughes Medical Institute University of California, Los Angeles, CA 90095, USA.

[e]Division of Chemistry and Chemical Engineering, California Institute of Technology, Pasadena, California 91125, USA

Table of Contents

Experimental procedures S6

1. Strains and culture conditions S6
2. General DNA manipulation techniques..S6
3. Heterologous expression of the *tre* gene cluster in *A. nidulans* S6
4. Heterologous biotransformation in *S. cerevisiae* S7
5. Chemical analysis settings and procedures S8
6. Expression and purification of TreE and TreC from *E. coli*................................S8
7. Expression and purification of TreA from *S. cerevisiae*.....................................S9
8. *In vitro* assays of TreE..S10
9. *In vitro* assays of TreA and TreC...S10
10. Isolation and structural elucidation S10
11. DFT calculation to investigate the (4+3) cycloaddition S12
12. Mosher method to determine the absolute stereochemistry of C-15...............S13
13. Microcrystal electron diffraction experiment for **3** S14
14. Sugar moiety determination S14
15. ECD calculations for possible enantiomers (**3a** and **3b**) of **3** S15
16. Expression and purification of microsomes containing TreB and TreD from *S. cerevisiae* and *in vitro* assays S15

Supplementary Tables.. S17

Table S1. Primers used in this study...S17

Table S2. Plasmids used in this study..S19

Table S3. ^{1}H (500 MHz) and ^{13}C NMR (125 MHz) for **1** in DMSO-d_6.................S20

Table S4. ^{1}H (500 MHz) and ^{13}C NMR (125 MHz) for **2** in DMSO-d_6.................S21

Table S5. ^{1}H (500 MHz) and ^{13}C NMR (125 MHz) for citrinovirin (**3**) in DMSO-d_6 ....S22

Table S6. ^{1}H (500 MHz) and ^{13}C NMR (125 MHz) for **4** in DMSO-d_6.................S23

Table S7. ^{1}H (500 MHz) and ^{13}C NMR (125 MHz) for **5** in DMSO-d_6.................S24

Table S8. ^{1}H (500 MHz) and ^{13}C NMR (125 MHz) for **7** in DMSO-d_6.................S25

Table S9. ^{1}H (500 MHz) and ^{13}C NMR (125 MHz) for **7a** in DMSO-d_6...............S26

Table S10. Homology of *tre* cluster and genes from the plant pathogens..............S27

Supplementary Figures.. S28

Figure S1. Homologous gene clusters in other *Trichoderma* sp. S28
Figure S2. Mosher derivatization to determine the absolute configuration of C-15. ....S29
Figure S3. ORTEP diagram of compound **3** from MicroED................................S30
Figure S4. Experimental ECD spectrum and calculated spectra of compound **3** . S31
Figure S5. Determination of the identity of the sugar ester.. ....S32
Figure S6. Heterologous expression in *A. nidulans* and feeding assay in *S. cerevisiae*, and mass spectra for the compounds **1-7**.. ....S33
Figure S7. Examples of reported trehalose lipids and glycoesters........................S35
Figure S8. *In vitro* assays of TreA and TreC. S36
Figure S9. Structural and sequential alignment of TreD.. ....S37
Figure S10. *In vitro* assays using microsomal fractions containing TreB and TreD S38
Figure S11. *In vitro* assays of recombinant TreE. S39
Figure S12. Geometries of compounds and transition states calculated by DFT...S40
Figure S13. ^{1}H NMR spectrum of compound **1** in DMSO-d_6 (500 MHz)............S41
Figure S14. ^{13}C NMR spectrum of compound **1** in DMSO-d_6 (125 MHz)...........S42
Figure S15. ^{1}H-^{13}C HSQC spectrum of compound **1** in DMSO-d_6 (500 MHz).....S43
Figure S16. ^{1}H-^{13}C HMBC spectrum of compound **1** in DMSO-d_6 (500 MHz)....S44
Figure S17. ^{1}H-^{1}H COSY spectrum of compound **1** in DMSO-d_6 (500 MHz)......S45
Figure S18. ^{1}H-^{1}H NOESY spectrum of compound **1** in DMSO-d_6 (500 MHz) ...S46
Figure S19. ^{1}H NMR spectrum of compound **2** in DMSO-d_6 (500 MHz)............S47
Figure S20. ^{13}C NMR spectrum of compound **2** in DMSO-d_6 (125 MHz)...........S48
Figure S21. ^{1}H-^{13}C HSQC spectrum of compound **2** in DMSO-d_6 (500 MHz).....S49
Figure S22. ^{1}H-^{1}H COSY spectrum of compound **2** in DMSO-d_6 (500 MHz)......S50
Figure S23. ^{1}H-^{13}C HMBC spectrum of compound **2** in DMSO-d_6 (500 MHz)....S51
Figure S24. ^{1}H-^{1}H NOESY spectrum of compound **2** in DMSO-d_6 (500 MHz) ...S52
Figure S25. ^{1}H NMR spectrum of compound **3** in DMSO-d_6 (500 MHz)............S53
Figure S26. ^{13}C NMR spectrum of compound **3** in DMSO-d_6 (125 MHz)...........S54
Figure S27. ^{1}H-^{13}C HSQC spectrum of compound **3** in DMSO-d_6 (500 MHz).....S55
Figure S28. ^{1}H-^{1}H COSY spectrum of compound **3** in DMSO-d_6 (500 MHz)......S56
Figure S29. ^{1}H-^{13}C HMBC spectrum of compound **3** in DMSO-d_6 (500 MHz)....S57
Figure S30. ^{1}H-^{1}H NOESY spectrum of compound **3** in DMSO-d_6 (500 MHz) ...S58
Figure S31. ^{1}H NMR spectrum of compound **4** in DMSO-d_6 (500 MHz)............S59

Figure S32. ^{13}C NMR spectrum of compound **4** in DMSO-d_6 (125 MHz)............S60
Figure S33. ^{1}H-^{13}C HSQC spectrum of compound **4** in DMSO-d_6 (500 MHz).....S61
Figure S34. ^{1}H-^{1}H COSY spectrum of compound **4** in DMSO-d_6 (500 MHz)......S62
Figure S35. ^{1}H-^{13}C HMBC spectrum of compound **4** in DMSO-d_6 (500 MHz)....S63
Figure S36. ^{1}H-^{1}H NOESY spectrum of compound **4** in DMSO-d_6 (500 MHz) ...S64
Figure S37. ^{1}H NMR spectrum of compound **5** in DMSO-d_6 (500 MHz).............S65
Figure S38. ^{13}C NMR spectrum of compound **5** in DMSO-d_6 (125 MHz)............S66
Figure S39. ^{1}H-^{13}C HSQC spectrum of compound **5** in DMSO-d_6 (500 MHz).....S67
Figure S40. ^{1}H-^{1}H COSY spectrum of compound **5** in DMSO-d_6 (500 MHz)......S68
Figure S41. ^{1}H-^{13}C HMBC spectrum of compound **5** in DMSO-d_6 (500 MHz)....S69
Figure S42. ^{1}H-^{1}H NOESY spectrum of compound **5** in DMSO-d_6 (500 MHz) ...S70
Figure S43. ^{1}H NMR spectrum of compound **7** in DMSO-d_6 (500 MHz).............S71
Figure S44. ^{13}C NMR spectrum of compound **7** in DMSO-d_6 (125 MHz)............S72
Figure S45. ^{1}H-^{13}C HSQC spectrum of compound **7** in DMSO-d_6 (500 MHz).....S73
Figure S46. ^{1}H-^{1}H COSY spectrum of compound **7** in DMSO-d_6 (500 MHz)......S74
Figure S47. ^{1}H-^{13}C HMBC spectrum of compound **7** in DMSO-d_6 (500 MHz)....S75
Figure S48. ^{1}H-^{1}H NOESY spectrum of compound **7** in DMSO-d_6 (500 MHz) ...S76
Figure S49. ^{1}H NMR spectrum of compound **7a** in DMSO-d_6 (500 MHz)...........S77
Figure S50. ^{13}C NMR spectrum of compound **7a** in DMSO-d_6 (125 MHz)..........S78
Figure S51. ^{1}H-^{13}C HSQC spectrum of compound **7a** in DMSO-d_6 (500 MHz)...S79
Figure S52. ^{1}H-^{1}H COSY spectrum of compound **7a** in DMSO-d_6 (500 MHz)....S80
Figure S53. ^{1}H-^{13}C HMBC spectrum of compound **7a** in DMSO-d_6 (500 MHz)..S81
Figure S54. ^{1}H-^{1}H NOESY spectrum of compound **7a** in DMSO-d_6 (500 MHz) .S82
Figure S55. ^{1}H NMR spectrum of compound **7b** in $CDCl_3$ (500 MHz)................S83
Figure S56. ^{13}C NMR spectrum of compound **7b** in $CDCl_3$ (125 MHz)...............S84
Figure S57. ^{1}H-^{13}C HSQC spectrum of compound **7b** in $CDCl_3$ (500 MHz)........S85
Figure S58. ^{1}H-^{1}H COSY spectrum of compound **7b** in $CDCl_3$ (500 MHz) S86
Figure S59. ^{1}H-^{13}C HMBC spectrum of compound **7b** in $CDCl_3$ (500 MHz).......S87
Figure S60. ^{1}H-^{1}H NOESY spectrum of compound **7b** in $CDCl_3$ (500 MHz).......S88
Figure S61. ^{1}H NMR spectrum of compound **7c** in $CDCl_3$ (500 MHz).................S89
Figure S62. ^{1}H-^{1}H COSY spectrum of compound **7c** in $CDCl_3$ (500 MHz)..........S90
Figure S63. ^{1}H NMR spectrum of compound **7d** in $CDCl_3$ (500 MHz)................S91
Figure S64. ^{1}H-^{1}H COSY spectrum of compound **7d** in $CDCl_3$ (500 MHz) S92

Supplementary Data S93

Data 1. Crystal data and structure refinement for **3** MicroED structure. S93

Data 2. Cartesian Coordinates and Energies S94

11'...S95

7' S97

IM1 ....S99

IM2 ....S102

IM3 ....S104

IM4 ....S106

TS1..S108

TS1_endo1 ....S110

TS1_endo2 ....S112

TS1_trans...S115

TS2..S117

Trans_int S119

References S122

Experimental procedures

1. Strains and culture conditions

Trichoderma afroharzianum T22 (ThT22) was obtained from the American Type Culture Collection (ATCC 20487) and grown in liquid PDB (potato dextrose broth) at 250 rpm, 28 °C for 3 days for cell proliferation for isolation of genomic DNA (gDNA) using Quick-DNA. *Aspergillus nidulans* (*AN*) A1145 ΔSTΔEM was grown at 28 °C on CD agar (1 L: 10 g glucose, 50 mL of 20X nitrate salts, 1 mL of trace elements, 20 g agar) or CDST agar (if use *glaA* promoter, 20 g starch, 20 g casamino acids (acidic digest), 50 mL 20X nitrate salts, 1 mL trace elements, 20 g agar.) for heterologous expression of the gene cluster. The *A. nidulans* transformants were grown in PDB medium at 250 rpm, 28 °C for 3 days for mRNA extraction. The 20X nitrate salts are prepared as: 120 g of $NaNO_3$, 10.4 g of KCl, 10.4 g of $MgSO_4•7H_2O$, 30.4 g of KH_2PO_4 dissolved in 1 L distilled water. The trace element solution was prepared as: 2.20 g of $ZnSO_4•7H_2O$, 1.10 g of H_3BO_3, 0.50 g of $MnCl_2•4H_2O$, 0.16 g of $FeSO_4•7H_2O$, 0.16 g of $CoCl_2•5H_2O$, 0.16 g of $CuSO_4•5H_2O$, and 0.11 g of $(NH_4)_6Mo_7O_{24}•4H_2O$ dissolved in 100 mL of distilled water, and the pH was adjusted to 6.5. All *Escherichia coli* strains were culture in LB media with carbenicillin or kanamycin antibiotic. Yeast strain were culture in YPD media (yeast extract 1%, peptone 2%, glucose 2%) at 220 rpm, 28 °C.

2. General DNA manipulation techniques

E. coli TOP10 were used for cloning, following standard recombinant DNA techniques. DNA restriction enzymes were used as recommended from the manufacturer (New England Biolabs, NEB). Q5 High Fidelity DNA polymerase (NEB) is used for all PCR reactions. The primers to construct the plasmids are listed in Table S1. The plasmids were sequenced by Laragen and Primordium Lab. *E. coli* BL21 (DE3) (Novagen) and *Saccharomyces cerevisiae* RC01 were used for protein expression. The *S. cerevisiae strain* JHY651 was used for homologous recombination to construct the plasmids for *A. nidulans* expression. *S. cerevisiae* RC01 was used for yeast expression.

The *A. nidulans* transformant containing the *tre* cluster was grown in PDB medium at 250 rpm, 28 °C for 4 days. The mRNA was extracted using RiboPure™ Yeast RNA Isolation Kit (Ambion) following the manufacturer's instructions. The cell pellet was lysed by nitrogen frozen followed with manually smashing. The extraction procedure was performed as instructed. The residual genomic DNA in the extracts was digested with DNase I (2U/μL) (Invitrogen) at 37 °C for 4 hours. SuperScript III First-Strand Synthesis System (Invitrogen) was used for cDNA synthesis with Oligo-dT primers following the instructions from the user manual.

3. Heterologous expression of the *tre* gene cluster in *A. nidulans*

Three plasmid vectors, pYTU, pYTP, and pYTR containing auxotrophic markers for uracil (*pyrG*), pyridoxine (*pyroA*), and riboflavin (*riboB*) respectively were

used to construct plasmids for *A. nidulans* heterologous expression. Genes in the *tre* cluster were amplified with PCR using gDNA from ThT22 as template. *gpdA* promoters from *Penicillium oxalicum (*constitutive *POgpdA), A. niger* (constitutive *gpdA*, *glaA* induced by starch) and *Penicillium expansum (*constitutive *PEgpdA)* were amplified by PCR. pYTP and pYTR were digested with *PacI*/*NotI*. pYTU was digested with *PacI*/*NotI* (keep *glaA* on vector) or *PShAI*/*NotI* (abolish glaA from vector). The amplified gene fragments and the corresponding vectors were co-transformed into *S. cerevisiae* JHY651 for homologous recombination. The yeast plasmids were extracted using Zymoprep™ Yeast Plasmid Miniprep I (Zymo Inc. USA), and then electrically transformed into *E. coli* TOP10 to isolate single plasmids. The plasmids were extracted from *E. coli* using the Zyppy™ Plasmid Miniprep Kit (Zymo Research) and confirmed with sequencing by Largen and Primordium Lab.

To prepare protoplasts, *A. nidulans* A1145 ΔSTΔEM was grown Oatmeal agar plates supplemented with 10 mM of uridine, 5 mM of uracil, 0.5 μg/mL of pyridoxine HCl and 2.5 μg/mL of riboflavin at 37 °C for 4 days. Fresh spores of *A. nidulans* A1145 ΔSTΔEM were inoculated into 25 mL of liquid CD media supplemented with 10 mM of uridine, 5 mM of uracil, 0.5 μg/mL of pyridoxine HCl and 2.5 μg/ml of riboflavin in a 125 mL flask and germinated at 28 °C 250 rpm for 16 h. Mycelia were harvested by centrifugation at 4000 rpm for 20 min and washed with 10 mL of osmotic buffer (1.2 M of $MgSO_4$, 10 mM of sodium phosphate, pH 5.8). The mycelia were transferred into 10 mL of osmotic buffer containing 30 mg of lysing enzymes from *Trichoderma* and 20 mg of Yatalase in a 125 mL flask. The cells were digested for 5 hours at 37 °C, 80 rpm. Cells were harvested in a 50 mL falcon tube and gently overlaid with 10 mL of trapping buffer (0.6 M of sorbitol, 0.1 M of Tris-HCl, pH 7.0). The cells were then centrifuged at 4300 rpm for 30 min at 4°C, the protoplasts were collected at the interface of the two buffers. The protoplasts were transferred to a sterile 15 mL falcon tube and washed with 3 volumes of STC buffer (1.2 M of sorbitol, 10 mM of $CaCl_2$, 10 mM of Tris-HCl, pH 7.5). After centrifugation at 4300 rpm, 20 min at 4°C, the supernatant was discarded, and the protoplast pellet was resuspended in 1 mL of STC buffer.

For each transformation, 3 μL of each plasmid (>100 ng/μL) was added to 60 μL of the *A. nidulans* A1145 ΔSTΔEM protoplast suspension prepared as above, and the mixture was incubated for 1 h on ice. 600 μL PEG solution (60% PEG, 50 mM of $CaCl_2$, and 50 mM of Tris-HCl, pH 7.5) was added to the protoplast mixture, followed by additional incubation at room temperature for 20 min. The mixture was spread on the CD sorbitol plate (CD solid medium with 1.2 M sorbitol and the appropriate supplements: 10 mM of uridine, 5 mM of uracil, 0.5 μg/mL of pyridoxine HCl, and/or 2.5 μg/mL of riboflavin according to the markers in the transformed plasmids) and incubated at 37 °C for 3-4 days.

4. Heterologous biotransformation in *S. cerevisiae*

To construct plasmids for heterologous expression in *S. cerevisiae*, two plasmid vectors, XW55 and XW06 with auxotrophic markers for uracil and tryptophan respectively. Both vectors are digested with *NdeI* and *XhoI*. The genes were amplified

using cDNA from the *A. nidulans* transformant with overhang primers. (Table S1) *ADH2* was used as promotors. The amplified gene fragments and the corresponding vectors were co-transformed into *S. cerevisiae* JHY651 for homologous recombination. The yeast plasmids were extracted using Zymoprep^{TM} Yeast Plasmid Miniprep I (Zymo Inc. USA), and then electrically transformed into *E. coli* TOP10 to isolate single plasmid. The plasmids were extracted from *E. coli* using the Zyppy^{TM} Plasmid Miniprep Kit (Zymo Research) and confirmed with sequencing by Largen and Primordium Lab. The plasmids (Table S2) were then transformed into *S. cerevisiae* RC01.

For small scale metabolite analysis of feeding experiments in *S. cerevisiae* RC01, 1 mL of the starter culture was cultivated overnight and then inoculated into 4 mL dropout media. Then all 4 mL culture was inoculated into 20 mL YPD. After incubated at 28 °C, 220 rpm for 48 h, the cells were collected by centrifugation at 4,000 rpm, 4 °C for 5 min, and the cell pellet was resuspended with 1 mL YPD medium. Then the substrate was added to the cultures in a final concentration of 200 µM and incubated for additional 24 h at 220 rpm and 28 °C. After centrifugation at 17,000 rpm for 5 min, the cell pellet was extracted with acetone and the supernatant was extracted with ethyl acetate. The combined extraction solvents were dried and then redissolved in methanol for LC-MS analysis.

5. Chemical analysis settings and procedures

For small scale metabolite analysis in *A. nidulans*, transformants were grown on CD agar or CDST agar for 4-5 days at 28 °C and then extracted with acetone. All the extracts were dried, and the residues were dissolved in MeOH for analysis. LC-MS analyses were performed on a Shimadzu 2020 EV LC-MS with a reverse phase column (Phenomenex Kinetex 1.7 µm C18 100 Å, LC Column 100 × 2.1 mm) using positive- and negative-mode electrospray ionization with a linear gradient of 5–95% CH_3CN-H_2O with 0.1% formic acid (v/v) in 15 min followed by 95% CH_3CN for 3 min with a flow rate of 0.3 ml/min. HERSIMS data and LC/MS data were also recorded on Agilent 1260 Infinity II LC equipped with an Infinity Lab Poroshell 120 EC-C18 column (2.7 µm, 3.0 × 50 mm) and a 6545 QTOF high resolution mass spectrometer (UCLA Molecular Instrumentation Center) using the solvent program (1% CH_3CN-H_2O 2 min, then 1−95% CH_3CN-H_2O (both with 0.1% formic acid, v/v) in 9 min followed by 95% CH_3CN-H_2O for 6 min at a flow rate of 0.8 mL/min). Semi-preparative HPLC was performed on an S7 UltiMateTM 3000 Semi-Preparative HPLC (ThermoFisher) using a COSMOSIL PBr column (5 µm, 250 ×10 mm), COMOSIL 5C18-AR-II (5 µm, 4.6 × 250 mm) or COSMOSIL 5C18-MS-II column (5 µm, 250 ×10 mm). Flow rate for HPLC was set at 3 mL/min.

6. Expression and purification of TreE and TreC from *E. coli*

The *treE* and *treC* genes were amplified with overhang primers from the cDNA of *A. nidulans* transformant. The PCR product and plasmid vector pET-28a (+) digested with *NdeI/XhoI* were assembled by NEBuilder® HiFi DNA Assembly Master Mix

(NEB). The mixture was incubated at 50°C for 60 min. The assembly product was transformed into *E. coli* TOP10 to isolate single plasmid. The plasmids were extracted from *E. coli* using the Zyppy™ Plasmid Miniprep Kit (Zymo Research) and confirmed with sequencing by Largen or Primordium Lab. The plasmid with correct sequence was transformed into *E. coli* BL21(DE3) cells for His6-tagged protein induction and purification. The *E. coli* cells harboring the corresponding plasmid were grown overnight in 10 mL LB medium with 50 µg/mL kanamycin at 37 °C, 220 rpm. Then the 10 mL of the starter culture was inoculated into 1 L of fresh LB medium with 50 µg/mL kanamycin and incubated at 37 °C, 220 rpm until the optical density OD_{600} reached around 0.6. Then the flask with the culture was submerged into ice water for 30 minutes. To induce the protein expression, 250 µL of 0.25 M isopropyl-β-D-thiogalactopyranoside (IPTG) was added into the 1 L culture. The culture was incubated at 16 °C, 220 rpm for 20 h. The cells were harvested by centrifugation at 5,000 rpm, 4 °C for 20 min, washed with 25 mL DI water and then resuspended in 30 mL lysis buffer (50 mM Tris-HCl, pH 7.5, 10 mM imidazole, 500 mM NaCl and 10% glycerol for TreB, and 50 mM PBS, 500 mM NaCl, 10 mM imidazole, 5% glycerol, pH 8.0 for TreC), and lysed by sonication on ice (1s on, 2s off, on time 5 min, amp 50% for 3 rounds). The lysate was centrifuged at 14,000 rpm, 4 °C for 30 min to remove the cellular debris. Then the supernatant was subjected to Ni-NTA affinity chromatography at 4 °C for protein purification according to the manufacturer's protocols (GE Healthcare). The purified protein was concentrated and exchanged into storage buffer (50 mM Tris-HCl, 50 mM NaCl, 10% glycerol, pH 7.5 for TreE, 50 mM PBS, 300 mM NaCl, 10% glycerol, pH 8.0 for TreC) using Amicon® Ultra-50K/10K Centrifugal Filters. The purity of protein was checked by SDS-PAGE, and the concentration was determined by Bradford method. The purified proteins were flash-frozen in liquid nitrogen and then stored at -80 °C.

7. Expression and purification of TreA from *S. cerevisiae*

The *treA* gene was amplified with overhang primers from cDNA of *A. nidulans* transformants. The plasmid vector XW55 with C-His (8H) was digested with *NdeI/NheI*. The amplified gene fragments and the corresponding vectors were co-transformed into *S. cerevisiae* JHY651 for homologous recombination. The plasmids were extracted using Zymoprep™ Yeast Plasmid Miniprep I (Zymo Inc. USA), and then electrically transformed into *E. coli* TOP10. The plasmids were extracted from *E. coli* using the Zyppy™ Plasmid Miniprep Kit (Zymo Research) and confirmed with sequencing by Primordium Lab. The plasmid with correct sequence was transformed into *S. cerevisiae* RC01. The transformant was grown on dropout plate for two days.

The *S. cerevisiae* RC01 transformant expressing *treA* was inoculated in 3 mL dropout media at 28 °C, 220 rpm, after growth of 20 hours, it was transferred into 50 ml dropout media at 28 °C, 220 rpm. After growth of 48 hours, the 50 ml culture was inoculated into 2 L YPD media and cultured at 28 °C, 220 rpm for 3 days before harvest. The cell pellet was collected by centrifuging at 5300 rpm and washed twice with 30 mL

DI water. The pellet was lysed by smashing in liquid nitrogen and resuspended in lysis buffer (50 mM PBS, 500 mM NaCl, 10 mM imidazole, 5% glycerol, pH 8.0), and further lysed with sonication (3s on, 6s off, on time 10 min, amp 55% for 3 rounds). The lysate was centrifuged at 15000 rpm, 4 °C for 60 min, and the supernatant was filtered with 0.45 nm filter to remove debris. Then the supernatant was subjected to Ni-NTA affinity chromatography at 4 °C for protein purification following the manufacturer's protocols (GE Healthcare). The purified protein was concentrated and exchanged into storage buffer (50 mM PBS, 300 mM NaCl, 10% glycerol, pH 8.0) by using Amicon® Ultra-100K Centrifugal Filters. The purity of protein was checked by SDS-PAGE, and the concentration was determined by Bradford method. The purified proteins were flash-frozen in liquid N_2 and stored at -80 °C.

8. *In vitro* assay of TreE

Enzymatic assays of TreE were performed in 50 mM sodium phosphate buffer (NaPB, pH 8.0) with a final volume of 100 μL containing 5 μM TreE, 10 mM Ca^{2+} or Mg^{2+}, 1mM substrate **7**. The reactions were incubated at 37 °C for 3h and extracted with 100 μL EtOAc for three times. The EtOAc extracts were dried and then redissolved in methanol for LC-MS analysis.

9. *In vitro* assay of TreA and TreC

Enzymatic assays of TreA and TreC were performed in 50 mM sodium phosphate buffer (NaPB, pH 8.0, 300 mM NaCl, 10% glycerol) with a final volume of 55 μL containing 8 μM TreA, 12 μM TreC, 2mM malonyl-CoA, 2 mM NADPH, 2mM SAM, 2 mM D-trehalose. The reactions were incubated at room temperature for 12h and extracted with 50 μL EtOAc for three times. The EtOAc extracts were dried and then redissolved in methanol for LC-MS analysis. For the base hydrolysis assay, 100 μl 0.5 M NaOH was added to the reaction mixture without TreC after 12h. After another 12h at room temperature, the mixture was extracted with 100 μL ethyl acetate for three times. The EtOAc extracts were dried and then redissolved in methanol for QTOF analysis.

10. Isolation and structural elucidation

Compound 1. *A. nidulans* transformant expressing *treAC* was cultured in 4 L CD-ST medium at 28 °C, 220 rpm for 6 days. The cell pellet was collected and extracted by acetone and the supernatant was extracted by EtOAc. The extracts were dried and then redissolved in 15 mL methanol. The insoluble impurities were discarded. 10 g of celite was added to the mixture and methanol was evaporated. The dried crude was purified with the CombiFlash system (Teledyne) using reverse phase gradient elution with water (A) and acetonitrile (B) (0-10 min 10-30% B; 10-15 min 30-50% B; 15-20 min 50% B; 20-30 min 100% B). Fractions containing **1** were combined and used for further purification by HPLC with a semi-preparative reverse-phase column

COSMOSIL C18-MSII 5 μm, 250 ×10 mm (CH_3CN:H_2O 50:50, v/v) to get **1** (~120 mg).

Compound 2. *A. nidulans* transformant expressing *treABCDE* was cultured in 5 L CD-ST medium at 28 °C, 220 rpm for 6 days. The cell pellet was collected and extracted by acetone and methanol (9:1) mixture and the supernatant was extracted by EtOAc. The extracts were dried and then redissolved in 15 mL methanol. The insoluble impurities were discarded. 10 g of celite was added to the mixture and methanol was evaporated. The dried crude was purified with the CombiFlash system (Teledyne) using reverse phase gradient elution with water (A) and acetonitrile (B) (0-10 min 10-30% B; 10-15 min 30-50% B; 15-20 min 50% B, 20-30 min 100% B). Fractions containing **2** were combined and used for further purification by HPLC with a semi-preparative reverse-phase column COSMOSIL C18-MSII 5 μm, 250 ×10 mm (CH_3CN:H_2O 18:82, v/v) to get **2** (~15 mg).

Compound 3. *A. nidulans* transformant expressing *treABCD* was cultured in 4 L CD-ST medium at 28 °C, 220 rpm for 6 days. The cell pellet was collected and extracted by acetone and the supernatant was extracted by EtOAc. The extracts were dried and then redissolved in 15 mL methanol. The insoluble impurities were discarded. 10 g of celite was added to the mixture and methanol was evaporated. The dried crude was purified with the CombiFlash system (Teledyne) using reverse phase gradient elution with water (A) and acetonitrile (B) (0-10 min 10-30% B; 10-15 min 30-50% B; 15-20 min 50% B, 20-30 min 100% B). Fractions containing **3** were combined and used for further purification by HPLC with a semi-preparative reverse-phase column COSMOSIL C18-MSII 5 μm, 250 ×10 mm (CH_3CN:H_2O 50:50, v/v) to get **3** (~20 mg).

Compound 4. *A. nidulans* transformant expressing *treABCDEG* was cultured in 5 L CD-ST medium at 28 °C, 220 rpm for 6 days. The cell pellet was collected and extracted by acetone and methanol (9:1) mixture and the supernatant was extracted by EtOAc. The extracts were dried and then redissolved in 15 mL methanol. The insoluble impurities were discarded. 10 g of celite was added to the mixture and methanol was evaporated. The dried crude was purified with the CombiFlash system (Teledyne) using reverse phase gradient elution with water (A) and acetonitrile (B) (0-10 min 10-30% B; 10-15 min 30-50% B; 15-20 min 50% B, 20-30 min 100% B). Fractions containing **4** were combined and used for further purification by HPLC with a semi-preparative reverse-phase column COSMOSIL C18-PBr 5 μm, 250 ×10 mm (CH_3CN:H_2O 18:82, v/v) to get **4** (~10 mg).

Compound 5. *A. nidulans* transformant expressing *treABCDEFG* was cultured in 5 L CD-ST medium at 28 °C, 220 rpm for 6 days. The cell pellet was collected and extracted by acetone and methanol (9:1) mixture and the supernatant was extracted by EtOAc. The extracts were dried and then redissolved in 15 mL methanol. The insoluble impurities were discarded. 10 g of celite was added to the mixture and methanol was evaporated. The dried crude was purified with the CombiFlash system (Teledyne) using

reverse phase gradient elution with water (A) and acetonitrile (B) (0-10 min 10-30% B; 10-15 min 30-50% B; 15-20 min 50% B, 20-30 min 100% B). Fractions containing **5** were combined and used for further purification by HPLC with a semi-preparative reverse-phase column COSMOSIL C18-MSII 5 μm, 250 ×10 mm (CH_3CN:H_2O 15:85, v/v) to get **5** (~5 mg).

Compound 7. *A. nidulans* transformant expressing *treABCD* was cultured in 4 L CD-ST medium at 28 °C, 220 rpm for 6 days. The cell pellet was collected and extracted by acetone and methanol (9:1) mixture and the supernatant was extracted by EtOAc. The extracts were dried and then redissolved in 15 mL methanol. The insoluble impurities were discarded. 10 g of celite was added to the mixture and methanol was evaporated. The dried crude was purified with the CombiFlash system (Teledyne) using reverse phase gradient elution with water (A) and acetonitrile (B) (0-10 min 10-30% B; 10-15 min 30-50% B; 15-20 min 50% B, 20-30 min 100% B). Fractions containing **7** were combined and used for further purification by HPLC with a semi-preparative reverse-phase column COSMOSIL C18-PBr 5 μm, 250 ×10 mm (CH_3CN: H_2O 17:83, v/v) to get **7** (~60 mg).

NMR spectra were obtained with a Bruker AV500 spectrometer with a 5 mm dual cryoprobe at the UCLA Molecular Instrumentation Center (^{1}H NMR 500 MHz, ^{13}C NMR 125 MHz). High resolution mass spectra were obtained from an Agilent LC-Q-TOF 6545 at the UCLA Molecular Instrumentation Center. Optical rotations were measured on a Rudolph Research Analytical Autopol III Automatic Polarimeter. ECD spectra were recorded on a Chirascan V100 spectropolarimeter in MeOH.

11. DFT calculation to investigate the (4+3) cycloaddition

All the calculations were carried out using Gaussian 16 program.[1] The geometries were optimized using ωB97X-D functional with def2-SVP basis set with IEEPCM solvent model to describe the water environment.[2-4] Conformational search using xTB and CREST was conducted for all the structures except water to ensure that the conformers we reported were in their lowest energy conformation.[5,6] The 20 conformers with the lowest energy, obtained from CREST, were subsequently re-optimized by DFT to determine the most stable conformer. For transition states, constraints are applied to the conformational search to preserve the TS vibration mode. Single point energies were calculated using ωB97X-D functional, def2-QZVPP basis set and SMD solvent model.[3,7] Quasiharmonic and concentration corrections to enthalpy and entropy were made using Paton's GoodVibes software.[8,9] For the hydronium ion involved in the reaction, proton solvation energy that reported by Truhlar *et. al.* was used, while the thermodynamic correction of free proton in gas phase was calculated using Fermi-Dirac distribution.[10,11]

12. Mosher method to determine the absolute stereochemistry of C-15 [12]

A mixture of 20 mg of **7** and 15 mg of NaOH in 1 mL MeOH was stirred at room temperature for 5 hours. The mixture was dried and the residue was purified by HPLC with a semi-preparative reverse-phase column COSMOSIL C18-MSII 5 μm, 250 ×10 mm (CH_3CN:H_2O 50:50, v/v). The fraction containing **7a** (10mg) was collected and dried. A mixture of 7 mg of **7a**,15 mg of *p*-toluenesulfonic acid (PTSA) and 300ul of 2,2-dimethoxypropane (slowly added while stirring) in 1mL of CH_2Cl_2 was stirred at 35 °C for 5 hours to produce **7b**. The mixture was dried and purified by HPLC with a semi-preparative reverse-phase column COSMOSIL C18-MSII 5 μm, 250 ×10 mm (0-10min: 40-100% MeOH, 10-20min: 100% MeOH). The fractions containing **7b** (6 mg) was collected and dried. To prepare the (*S*)- and (*R*)-MTPA esters (**7c** and **7d**), a mixture of **7b** (3 mg), (*R*)-MTPACl (10ul), 4-(dimethylamino) pyridine (10 mg) in 1 mL of pyridine was stirred at 35 °C for 5 h to produce **7c** (2mg). The mixture was then dried and purified by HPLC with a semi-preparative reverse-phase column COSMOSIL C18-MSII 5 μm, 250 ×10 mm (MeOH: H_2O 90:10, v/v). Similarly, the (*R*)-MTPA esters (**7d**, 2mg) was prepared (Table S9, Figure S7, S73-88).

7b: White power; ^{1}H NMR (500 MHz, $CDCl_3$) δ_H 6.86 (1H, ddd, 9.1, 5.7, 1.4 Hz, H-3), 5.46 (1H, ddd, 10.8, 8.7, 0.9 Hz, H-14), 5.19 (1H, td, 10.6, 1.1 Hz, H-13), 4.72 (1H, dtd, 9.2, 6.9, 5.7 Hz, H-15), 4.25 (1H, t, 7.6 Hz, H-8), 3.98 (1H, dd, 7.6, 5.8 Hz, H-7), 3.72 (3H, s, H-20), 3.39 (1H, m, H-12), 2.34 (1H, ddd, 14.0, 9.1, 1.8 Hz, H-4a), 2.26 (1H, m, H-10), 2.26 (1H, m, H-11), 2.12 (1H, ddd, 13.9, 11.4, 5.8 Hz, H-4b), 1.84 (1H, ddd, 11.9, 7.6, 3.9 Hz, H-9), 1.61 (1H, dt, 11.1, 6.2 Hz, H-6), 1.50 (3H, s, H-22), 1.29 (3H, s, H-23), 1.23 (3H, d, H-16), 1.07 (3H, m, H-17), 1.07 (1H, m, H-5), 0.82 (3H, d, 7.0 Hz, H-18), 0.76 (3H, d, 6.4 Hz, H-19); HRESIMS *m/z* 415.2454 $[M+Na]^+$ (calcd for $C_{23}H_{36}NaO_5$, 415.2455).

(*S*)-MTPA ester (**7c**): White power, ^{1}H NMR (500 MHz, $CDCl_3$) δ_H 6.09 (1H, m, H-15), 5.51 (1H, ddd, 10.4, 9.4, 0.8 Hz, H-14), 5.36 (1H, m, H-13), 1.39 (3H, d, H-16); HRESIMS *m/z* 631.2846 $[M+Na]^+$ (calcd for $C_{33}H_{43}F_3NaO_7$, 631.2853).

(*R*)-MTPA ester (**7d**): White power, ^{1}H NMR (500 MHz, $CDCl_3$) δ_H 6.02 (1H, m, H-15), 5.31 (1H, m, H-14), 5.31 (1H, m, H-13), 1.32 (3H, d, 6.5 Hz, H-16); HRESIMS *m/z* 631.2853 $[M+Na]^+$ (calcd for $C_{33}H_{43}F_3NaO_7$, 631.2853).

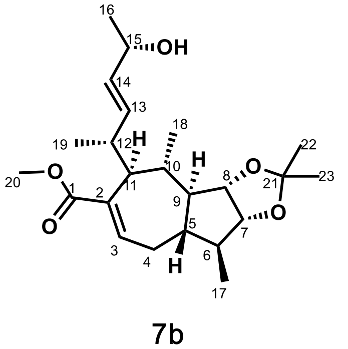

7b

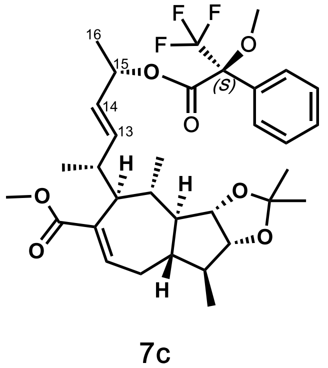

7c

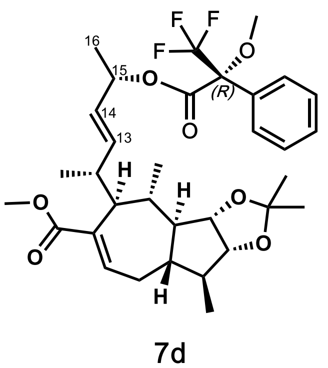

7d

13. Microcrystal electron diffraction experiment for 3

Compound **3** was dissolved in 500 μL of solvent (90% acetonitrile and 10% H_2O). The cap of the vial was loosened and the vial was left at room temperature for 3 days. Crystal was formed on the wall of the vial.

Samples for MicroED was prepared by dropping a 200-mesh continuous carbon on copper grid into a vial and shaking it, in accordance to previously disclosed procedures outlined in Jones et. al.[13] Data was collected on a Thermo Fisher Talos Arctica F200C transmission electron microscope operating at 80 K with an accelerating voltage of 200 keV, corresponding to an electron wavelength of 0.0251 Å. Electron diffraction data was collected using a Thermo Fisher CetaD camera operating in rolling shutter mode. Particles were visually selected for data collection, isolated by a selected area aperture, and adjusted to eucentric height to stay within the aperture while tilting the sample. Data was collected by taking 3 second images of the diffraction patterns generated by a continuously rotating crystal. This rotation was performed at a rate of 0.3° per second with a minimum and maximum tilt range of −70° to +70°. All diffraction data was processed using the XDS suite of programs as controlled by a custom Python automation script.[14-16] The structure was solved ab initio by direct methods in SHELXD. After this, the structure was refined with SHELXL using ShelXle and incorporating electron scattering factors.[17-19] Thermal parameters were refined anisotropically for all non-hydrogen atoms. Hydrogen atoms were assigned using the riding model.

Refinement of F^2 against ALL reflections. The weighted R-factor (wR) and goodness of fit (S) are based on F-^{2}, conventional R-factors (R) are based on F, with F set to zero for negative F^2. The threshold expression of F^2 > 2s(F^2) is used only for calculating R-factors(gt) etc. and is not relevant to the choice of reflections for refinement. R-factors based on F^2 are statistically about twice as large as those based on F, and R-factors based on ALL data will be even larger.

All esds (except the esd in the dihedral angle between two l.s. planes) are estimated using the full covariance matrix. The cell esds are taken into account individually in the estimation of esds in distances, angles and torsion angles; correlations between esds in cell parameters are only used when they are defined by crystal symmetry. An approximate (isotropic) treatment of cell esds is used for estimating esds involving l.s. planes.

14. Sugar moiety determination[20]

To obtain the sugar moiety from compounds **1** and **4,** 0.1 mg of each compound was dissolved in 550 μL water and methanol mixture (100 μL MeOH, 450 μL water) with 2 M HCl. The mixture was stirred at 60 °C for 4 h. The mixture was then extracted

with chloroform and the aqueous layer containing sugar was collected and dried. To derivatize the sugar, the dried sugar or sugar standards (2 mg) were heated with 1 mg of L-cysteine methyl ester in 400 μL of pyridine and stirred at 60 °C for 1 h. 200 μl *o*-tolyl isothiocyanate was added to the mixture and stirring was continued at 60 °C for 1 h. The solution was directly analyzed on HPLC [Thermo Fisher COMOSIL 5C18-AR-II (4.6 × 250 mm), CH_3CN:H_2O. (25:75), flow rate 0.8 mL/min, UV detection 250 nm, column temperature 35 °C]. The retention time of L-glucose derivative was about 16.3 min, and the retention time of D-glucose derivative was ~ 17.9 min (Figure S12).

15. ECD calculations for possible enantiomers (3a and 3b) of 3

Conformational searches and DFT calculations were carried out on Spartan 20 program (Wavefunction Inc., Irvine, CA, USA) and Gaussian 16 program,[1] respectively. Conformational searches for possible enantiomers {(2*S*,5*S*,6*S*,7*R*,8*S*,9*R*,10*R*,11*R*,12*R*)-**3a** and (2*R*,5*R*,6*R*,7*S*,8*R*,9*S*,10*S*,11*S*,12*S*)-**3b**} of **3** were conducted at Molecular Mechanics with MMFF to give initial conformers, which were optimized by the DFT method at the B3LYP/6-31G(d) level in the presence of MeOH with a universal solvation model (SMD). Rotatory strengths of the stable conformers with Boltzmann distributions over 1% were calculated by the TDDFT method at the B3LYP/6-31G(d) level with SMD (MeOH). The resultant rotatory strengths of the lowest 30 excited states for each conformer were converted into Gaussian-type curves with half bands (0.3 ev) by SpecDis v1.71 program.[21] The ECD spectra were composed after corrections based on the Boltzmann distributions of the stable conformers. The calculated ECD spectra were shifted by –10 nm. The ECD spectrum of **3** was correlated with the calculated spectrum of **3a**, indicating the (2*S*,5*S*,6*S*,7*R*,8*S*,9*R*,10*R*,11*R*,12*R*) configuration of **3**. This configuration matches reported data of citrinovirin.[22]

16. Expression and purification of microsomes containing TreB and TreD from *S. cerevisiae* and *in vitro* assays

The plasmid XW55-129.1-B and XW06-129.1-D were introduced into *S. cerevisiae* RC01 both independently and together. The *S. cerevisiae* RC01 was cultivated in 5 mL of selective dropout medium at 28 °C for 20 h. 4 mL of the starter culture was inoculated into 50 mL YPD medium for an additional 48 h at 28 °C, 220 rpm. The cells were harvested by centrifugation at 4,300 rpm, 4 °C for 5 min, and the cell pellet was washed with 5 mL TEGM buffer (50 mM Tris-HCl, pH 7.5, 1 mM EDTA, 1.5 mM *β*-mercaptoethanol, 20% glycerol (v/v), Pierce™ protease inhibitor) and then resuspended in 4 mL TEGM buffer. Glass beads (~ 400 μL, 0.5 mm in diameter) were added, and the cell walls were disrupted by vortex with 1 min separated by 1 min intervals on ice for a total of 12 min. The mixture was centrifuged at 5000 rpm, 4 °C for 5 min to remove the beads and cell debris. Then the supernatant was collected and centrifuged again at 17,000 rpm 4 °C for 1 h to further remove the cell debris. The supernatant was collected and centrifuged at 80,000 and 4 °C for 40 min. Transparent

microsome residue was collected and resuspended in 50 mM Tris-HCl (pH 7.5, 50 mM NaCl, 10% glycerol, v/v). To characterize the function of TreB and TreD, the reaction was performed in 50 mM NaPB (pH 7.6) with a final volume of 500 μL, containing 50 μL of microsomal fractions, 200 μM **1**, and 250 μM NADPH. The reactions were incubated at 28 °C for 12 h and extracted two times with 500 μL of EtOAc. The EtOAc extracts were evaporated to dryness and then redissolved in methanol for LC-MS analysis.

Supplementary Tables

Table S1. Primers used in this study.

Primer name	Sequence (5'→3')
129.1-U-A-1-F	catccccagcatcattacacctcagcattaattaaatggggagcatatcaaatcatggg
129.1-U-A-1-R	aatcgaggagcgttcacgtc
129.1-U-A-2-F	gctccagtacccgactgggacgtgaacgctcctcgattccgcaactttctgcgaatctcc
129.1-U-A-2-R	tttggcgactgtcgattgatcca
129.1-U-A-3-F	gcacacgtatggtcgtttggatcaatcgacagtcgccaaagtccccgatggactcagtct
129.1-U-A-3-R	cctgcagcccgggggatccactagttctagagcggccgcccttgcagttcactgggga cg
129.1-A-gpd-F	cattaccccgccacatagacacatctaaacaatggggagcatatcaaatcatgg
129.1-A-1-R	tcagcgctgctattcacgtc
129.1-A-2-F	attcctcccggcactgaaac
129.1-A-2-R	tcaactggaagagtgcgcgagaac
129.1-A-3-F	acttggacgattccaacctggtc
129.1-A-PO-R	tcagtaagctcacatgtattcctggagcaaacacaagcatctactttgttagtcttgcag
PO-F	tttgctccaggaatacatgtgagcttactg
PO-R	ttttgcgattgtttgaagtgttctgtatgc
129.1-C-PO-F	gcatacagaacacttcaaacaatcgcaaaaatgaagatcctctgccttcacg
129.1-C-pyr-R	acacagtggaggacatacccgtaattttctgttgtggaaggaagcgttacgc
129.1-R-gpdA-F	ctcgcgggtgttcttgacgatggcatcctgcggccgcactccggtgaattgatttgggtg
129.1-R-B-R	atcgaattcctgcagcccgggggatccttaattaagcaaaactactgtcgttgctcaagg
129.1-B-gpd-F	attaccccgccacatagacacatctaaacaatggccaccatgagcgac
129.1-B-PO-R	cagtaagctcacatgtattcctggagcaaagtcgaccatgcaaaactactgtcg
129.1-E-PO-F	gcatacagaacacttcaaacaatcgcaaaaatgaccagagacgggtcctg
129.1-R-E-R	gatatcgaattcctgcagcccgggggatccttaattaaccaacgctgtacttcactctgg
129.1-R-PO-F	cgcgggtgttcttgacgatggcatcctgcggccgctttgctccaggaatacatgtgagct
PE-F	gattcgtccagggcttccca
PE-R	gattgcggtttactagaagttgttagatcatgtataa
129.1-BEF-B-R	ccaatattccaaccttgggaagccctggacgaatcgcaaaactactgtcgttgctcaagg
129.1-P-DFG-F-F	ctcttatacatgatctaacaacttctagtaaaccgcaatcatgtctcaagacggcgagcc
129.1-P-DFG-F-R	gtagaatcagtaagctcacatgtattcctggagcaaacaacacgtgacccttacatgtgt
129.1-D-gpd-F	attaccccgccacatagacacatctaaacaatggatgccctcacgctg
129.1-D-pyro-R	gatgagacccaacaaccatgataccaggggtagatctcccatgccatcgttgtttgag
129.1-P-DFG-D-R	gtccccaatattccaaccttgggaagccctggacgaatccccaatggcatccttccacca
P-129.1-DF-F-F	gcttacaagtgcatacagaacacttcaaacaatcgcaaaaatgtctcaagacggcgagc
129.1-P-DFG-F-R	gtagaatcagtaagctcacatgtattcctggagcaaacaacacgtgacccttacatgtgt
129.1-P-DFG-G-F	cttacaagtgcatacagaacacttcaaacaatcgcaaaaatgttctcttccgacagcacc
129.1-P-DFG-G-R	cttgatatcgaattcctgcagcccgggggatccttaattaactactaccccgcaccgtcc

129.1-P-DG-D-R	aaagtagaatcagtaagctcacatgtattcctggagcaaacccaatggcatccttccacc
XW55-129.1-B-F	atcaactatcaactattaactatatcgtaataccatgctagcatggccaccatgagcgac
XW55-129.1-B-R	aaaaaaaaaaaaatctttgactattcaatcattgcgctcataattctctccgcttcaggcg
XW55-1a-CPS1t-R	acgacggccagtgaattcgagctcggtaccctcgagatttgacacttgatttgacacttc
CPS1t-F	gcgcaatgattgaatagtcaaagatt
XW06-129.1-D-F	caatcaactatcaactattaactatatcgtaataccatcatatggatgccctcacgctgg
XW06-129.1-D-R	aaaaaatctttgactattcaatcattgcgcctagaaacccaaaacaaatgcatctctctc
XW06-2a-CPS1t-R	cactatagggcgaattgggtaccctcgaatttgacacttgatttgacacttctttttttt
pET28a-N-His-129.1-E-F	cagcggcctggtgccgcgcggcagccatatgatgaccagagacgggtcctg
pET28a-N-His-129.1-E-R	gatctcagtggtggtggtggtggtgctcgagctacacatcagttcctttcctctggc
pET28a-hydrolase-CHis-F	gaaataattttgtttaactttaagaaggagatataccatgaagatcctctgccttcacgg
pET28a-hydrolase-CHis-R	ccggatctcagtggtggtggtggtggtgctcgagtagacttgttgtagcttcagcgacc
XW55-treA-1-F	ctatcaactattaactatatcgtaataccatatgatggggagcatatcaaatcatgggc
XW55-treA-1-R	attccacgattgcagcaaatcca
XW55-treA-2-F	cttcaagttgcgcttgtggatttgctgcaatcgtggaatatcaagcccagtcgtgtcgtg
XW55-treA-2-R	gctggccaaatgatagcccg
XW55-treA-3-F	cgggctatcatttggccagctgccagggtggtggaatggcactgaatccatgcgccaatg
XW55-treA-3-R	tttgtcatttaaattagtgatggtgatggtgatggtgatggctagctgccgccggcaact

Table S2. Plasmids used in this study.

Plasmid name	Vector	Genes
129.1-P-D	pYTP	*treD*
129.1-P-DFG	pYTP	*treDFG*
129.1-R-B	pYTR	*treB*
129.1-R-E	pYTR	*treE*
129.1-R-BE	pYTR	*treBE*
129.1-R-BEF	pYTR	*treBEF*
129.1-U-A	pYTU	*treA*
129.1-U-AC	pYTU	*treAC*
XW55-129.1-B	XW55	*treB*
XW06-129.1-D	XW06	*treD*
pET28a-NHis-129.1-E	pET28a(+)	*treE*
pET28a-CHis-129.1-C	pET28a(+)	*treC*
XW55-TreA	XW55	*treA*

Table S3. ^{1}H (500 MHz) and ^{13}C NMR (125 MHz) for **1** in DMSO-*d6*

yellow gum. $[\alpha]^{22}_{D}$ + 30 (*c* 0.1, MeOH). HRESIMS *m/z* 635.3039 $[M+Na]^+$ (calcd for $C_{31}H_{48}NaO_{12}$, 635.3038).

selected 2D NMR correlation

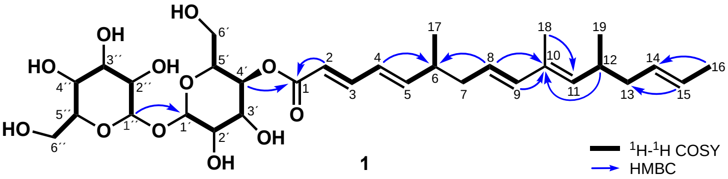

	**1**	
position	^{13}C	^{1}H (*J* in Hz)
1	165.6	–
2	119.5	5.91 (1H, d, 15.3)
3	145.3	7.19 (1H, m)
4	126.5	6.22 (1H, m)
5	149.9	6.22 (1H, m)
6	39.3	2.35 (1H, h, 6.8)
7	40.0	2.16 (1H, m)
		2.06 (1H, dt, 14.2, 7.3)
8	124.9	5.46 (1H, dt, 15.0, 7.2)
9	136.4	6.01 (1H, d, 15.5)
10	131.6	–
11	136.7	5.17 (1H, d, 9.3)
12	36.9	2.44 (1H, m)
13	40.0	1.92 (2H, t, 6.2)
14	129.4	5.34 (1H, m)
15	125.8	5.34 (1H, m)
16	12.6	1.58 (3H, dd, 5.6, 1.2)
17	20.5	0.98 (3H, d, 6.7)
18	17.8	1.64 (3H, d, 1.2)
19	32.3	0.88 (3H, d, 6.6)
1′	93.0	4.89 (1H, m)
2′	72.0	3.34 (1H, m)
3′	70.2	3.75 (1H, td, 9.2, 4.8)
4′	71.3	4.68 (1H, m)
5′	70.4	3.89 (1H, ddd, 10.3, 5.4,2.4)
6′	60.7	3.34 (1H, m)
		3.25 (1H, m)
1′′	93.3	4.89 (1H, m)
2′′	71.6	3.13 (1H, td, 8.7, 3.0)
3′′	72.8	3.55 (1H, dd, 11.0, 7.0)
4′′	70.0	3.25 (1H, ddt, 9.2, 5.8, 3.6)
5′′	72.6	3.65 (1H, ddd, 10.1, 4.9, 2.3)
6′′	60.7	3.47 (1H, m)

Table S4. ^{1}H (500 MHz) and ^{13}C NMR (125 MHz) for **2** in DMSO-d_6

white powder; $[\alpha]^{22}_{D}$ + 4 (*c* 0.1, MeOH). HRESIMS *m/z* 523.2513 $[M+Na]^+$ (calcd for $C_{25}H_{40}NaO_{10}$, 523.2514).

selected 2D NMR correlation

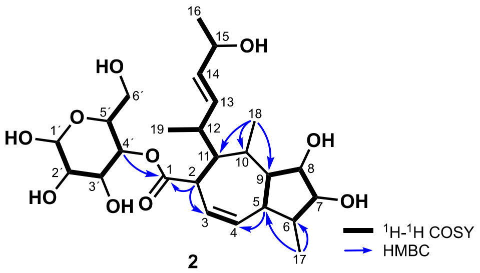

2

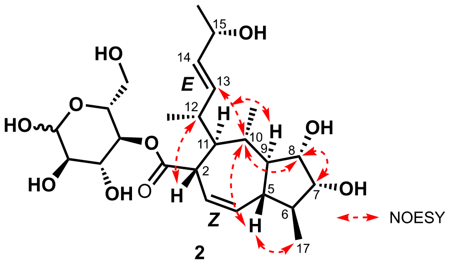

2

	2a			2b	
position	^{13}C	^{1}H (*J* in Hz)		^{13}C	^{1}H (*J* in Hz)
1	173.9	–		173.9	–
2	49.5^a	3.19 (1H, m)		49.4^a	3.19 (1H, m)
3	131.8^b	5.46 (1H, ddt, 12.0, 10.3, 2.5)		131.7^b	5.46 (1H, ddt, 12.0, 10.3, 2.5)
4	132.2	5.59 (1H, dt, 12.0, 1.8)		132.2	5.59 (1H, dt, 12.0, 1.8)
5	41.4	1.48 (1H, m)		41.4	1.48 (1H, m)
6	45.6	1.35 (1H, m)		45.6	1.35 (1H, m)
7	76.4	3.18 (1H, m)		76.4	3.18 (1H, m)
8	72.2	3.39 (1H, m)		72.2	3.39 (1H, m)
9	55.6	1.48 (1H, m)		55.6	1.48 (1H, m)
10	30.4^c	2.25 (1H, qd, 7.0, 2.5)		30.2^c	2.25 (1H, qd, 7.0, 2.5)
11	50.4	1.84 (1H, ddt, 9.3, 4.7, 2.1)		50.4	1.84 (1H, ddt, 9.3, 4.7, 2.1)
12	35.1	2.55 (1H, dtd, 10.4, 7.0, 3.2)		35.1	2.55 (1H, dtd, 10.4, 7.0, 3.2)
13	132.6^d	5.07 (1H, d, 6.4)		132.5^d	5.07 (1H, d, 6.4)
14	134.3	5.21 (1H, m)		134.3	5.21 (1H, m)
15	62.4	4.43 (1H, m)		62.4	4.43 (1H, m)
16	17.0	1.04 (3H, m)		17.0	1.04 (3H, m)
17	24.7	0.99 (3H, m)		24.7	0.99 (3H, m)
18	8.9	0.71 (1H, dd, 7.0, 3.2)		8.9	0.71 (1H, dd, 7.0, 3.2)
19	21.0	0.87 (3H, d, 6.8)		21.0	0.87 (3H, d, 6.8)
1'	96.9	4.93 (1H, d, 3.6)		92.1	4.33 (1H, d, 7.7)
2'	75.1	3.18 (1H, m)		75.1	3.18 (1H, m)
3'	70.9	3.62 (1H, td, 9.5, 5.6)		70.9	3.62 (1H, td, 9.5, 5.6)
4'	74.5	4.59 (1H, m)		74.2	4.59 (1H, m)
5'	69.6	3.75 (1H, ddd, 10.2, 7.9, 6.1, 2.7)		69.6	3.75 (1H, ddd, 10.2, 7.9, 6.1, 2.7)
6'	60.8	3.18 (2H, m)		60.8	3.18 (2H, m)

[a–d] Interchangeable.

Compound **2** readily epimerizes at the anomeric C-1' to give an equilibrium mixture of epimers (**2a** and **2b**) in DMSO-d_6. Therefore, the structure elucidation was carried out using the spectroscopic data for the mixture of **2a** and **2b**.

Table S5. ^{1}H (500 MHz) and ^{13}C NMR (125 MHz) for citrinovirin (**3**) in DMSO-d_6

white powder; $[\alpha]^{22}_{D}$ + 14 (*c* 0.1, MeOH). HRESIMS *m/z* 345.2031 $[M+Na]^+$ (calcd for $C_{19}H_{30}NaO_4$, 345.2036).

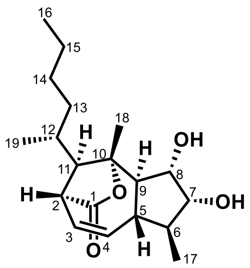

citrinovirin (3)

	citrivinorin (**3**) (exp.)			citrivinorin (**3**) [22]	
position	^{13}C	^{1}H (*J* in Hz)		^{13}C	^{1}H (*J* in Hz)
1	174.6	–		175.5	–
2	46.4	3.33 (1H, m)		47.4	3.36 (1H, m)
3	124.7	5.70 (1H, m)		125.6	5.74 (1H, ddd, 11.0, 7.6, 2.6)
4	133.2	5.81 (1H, dd, 11.1, 1.8)		134.1	5.85 (1H, dd, 11.1, 1.7)
5	42.9	2.05 (1H, brt, 12.9)		43.8	2.09 (1H, brt, 12.2)
6	45.4	1.35 (1H, m)		46.3	1.39 (1H, m)
7	74.9	3.27 (1H, m)		75.8	3.31 (1H, ddd, 7.1, 7.1, 7.1)
8	71.5	3.77 (1H, ddd, 8.7, 5.2)		72.4	3.81 (1H, ddd, 8.8, 8.2, 6.6)
9	61.5	2.14 (1H, m)		62.4	2.19 (1H, dd, 12.8, 9.4)
10	90.7	–		91.6	–
11	60.1	2.17 (1H, m)		61.0	2.21 (1H, dd, 10.6, 5.8)
12	30.9	1.94 (1H, m, 13.1, 8.6, 5.0)		31.8	1.98 (1H, m)
13	34.0	1.24 (1H, m)		34.9	1.28 (1H, m)
		1.43 (1H, m)			1.47 (1H, m)
14	28.4	1.15 (1H, m)		29.3	1.19 (1H, m)
		1.42 (1H, m)			1.46 (1H, m)
15	22.1	1.28 (2H, m)		23.0	1.32 (2H, m)
16	14.0	0.89 (3H, m)		14.9	0.92 (3H, t, 7.2)
17	16.4	1.03 (3H, d, 6.6)		17.3	1.07 (3H, d, 6.6)
18	25.8	1.70 (3H, s)		26.7	1.74 (3H, s)
19	19.3	0.85 (3H, m)		20.2	0.89 (3H, d, 6.3)
7-OH	-	4.80 (1H, m)		-	4.78 (1H, d, 6.4)
8-OH	-	4.56 (1H, m)		-	4.54 (1H, d, 6.4)

Isolated **3** has near identical NMR chemical shifts as reported for citrinovirin in SI reference 22. A similar ECD spectrum was also obtained, but we measured the opposite optical rotation as reported. Based on MicroED and ECD calculation, we determined compound **3** has the same absolute structure as reported for citrinovirin.

Table S6. ^{1}H (500 MHz) and ^{13}C NMR (125 MHz) for **4** in DMSO-*d*$_6$

yellow gum; $[\alpha]^{22}_{D}$ + 16 (*c* 0.1, MeOH). HRESIMS *m/z* 499.2540 [M-OH]$^+$ (calcd for $C_{25}H_{38}O_{10}$, 499.2538).

selected 2D NMR correlation

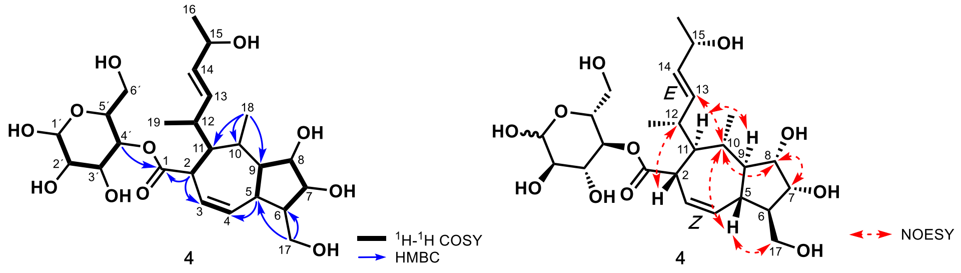

	**4a**			**4b**	
position	^{13}C	^{1}H (*J* in Hz)		^{13}C	^{1}H (*J* in Hz)
1	173.9	–		173.9	–
2	49.5^a	3.46 (1H, m)		49.4^a	3.46 (1H, m)
3	126.3^b	5.60 (1H, tt, 12.1, 3.2)		126.1^b	5.60 (1H, tt, 12.1, 3.2)
4	132.6	5.39 (1H, m)		132.6	5.39 (1H, m)
5	36.1	2.04 (1H, m)		36.1	2.04 (1H, m)
6	63.1	1.65 (1H, m)		63.1	1.65 (1H, m)
7	72.3	3.80 (1H, m)		72.3	3.80 (1H, m)
8	72.1	3.46 (1H, m)		72.1	3.46 (1H, m)
9	54.0	1.76 (1H, m)		54.0	1.76 (1H, m)
10	29.9^c	2.45 (1J, d, 7.0)		29.7^c	2.45 (1H, d, 7.0)
11	50.4	2.04 (1H, m)		50.4	2.04 (1H, m)
12	35.1	2.74 (1H, s)		35.1	2.74 (1H, s)
13	133.3	5.84 (1H, dt, 12.1, 2.6)		133.3	5.84 (1H, dt, 12.1, 2.6)
14	134.2	5.39 (1H, m)		134.2	5.39 (1H, m)
15	72.3	4.60 (1H, p, 6.4)		72.3	4.60 (1H, p, 6.4)
16	24.7	1.22 (1H, d, 6.2)		24.7	1.22 (1H, d, 6.2)
17	61.1^d	3.46 (1H, m)		60.8^d	3.46 (1H, m)
18	8.7	0.90 (3H, dd, 7.2, 3.0)		8.7	0.90 (3H, dd, 7.2, 3.0)
19	21.1	1.05 (3H, d, 6.7)		21.1	1.05 (3H, d, 6.7)
1'	96.9^e	5.12 (1H, d, 3.6)		92.1^e	4.52 (1H, m)
2'	75.1	3.17 (1H, m)		75.1	3.17 (1H, m)
3'	70.1	3.45 (1H, m)		70.1	3.45 (1H, m)
4'	74.3	4.76 (1H, m)		74.3	4.76 (1H, m)
5'	69.6	3.63 (1H, m)		69.6	3.63 (1H, m)
6'	60.8	3.46 (1H, m)		60.8	3.46 (1H, m)

$^{a–e}$ Interchangeable.

Compound **4** readily epimerizes at the anomeric C-1' to give an equilibrium mixture of epimers (**4a** and **4b**) in DMSO-*d*$_6$. Therefore, the structure elucidation was carried out using the spectroscopic data for the mixture of **4a** and **4b**.

Table S7. ^{1}H (500 MHz) and ^{13}C NMR (125 MHz) for **5** in DMSO-*d*$_6$

white powder; $[\alpha]^{22}_{D}$ + 18 (*c* 0.1, MeOH). HRESIMS *m/z* 567.2417 [M+Na]$^+$ (calcd for $C_{26}H_{40}NaO_{12}$, 567.2412).

selected 2D NMR correlation

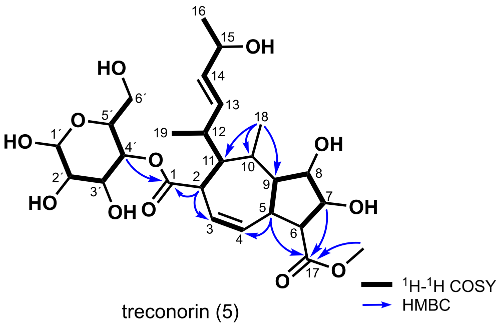

treconorin (5)

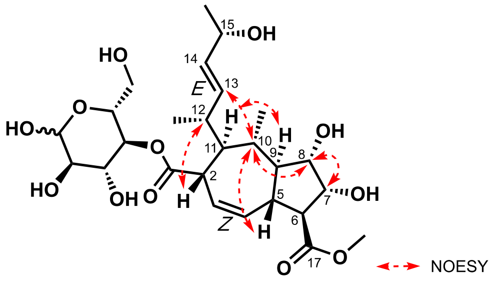

treconorin (5)

	5a			5b	
position	^{13}C	^{1}H (*J* in Hz)		^{13}C	^{1}H (*J* in Hz)
1	173.6	–		173.6	–
2	49.5^a	3.3 (1H, m)		49.4^a	3.3 (1H, m)
3	127.6^b	5.52 (1H, m)		127.4^b	5.52 (1H, m)
4	130.9	5.52 (1H, m)		130.9	5.52 (1H, m)
5	38.1	2.17 (1H, m)		38.1	2.17 (1H, m)
6	56.1	2.31 (1H, m)		56.1	2.31 (1H, m)
7	72.4	3.92 (1H, dd, 6.7, 4.3)		72.4	3.92 (1H, dd, 6.7, 4.3)
8	72.3	3.41 (1H, m)		72.3	3.41 (1H, m)
9	54.0	1.62 (1H, m)		54.0	1.62 (1H, m)
10	29.9^c	2.31 (1H, m)		29.8^c	2.31 (1H, m)
11	50.1	1.88 (1H, ddd, 12.0, 5.9, 3.6)		50.1	1.88 (1H, ddd, 12.0, 5.9, 3.6)
12	35.1	2.57 (1H, dtd, 10.1, 6.7, 3.2)		35.1	2.57 (1H, dtd, 10.1, 6.7, 3.2)
13	132.5.	5.12 (1H, m)		132.5.	5.12 (1H, m)
14	134.3	5.23 (1H, m)		134.3	5.23 (1H, m)
15	62.4	4.44 (1H, p, 6.5)		62.4	4.44 (1H, p, 6.5)
16	24.7	1.06 (1H, d, 6.1)		24.7	1.06 (1H, d, 6.1)
17	174.4	–		174.4	–
18	8.5	0.75 (3H, dd, 7.2, 2.9)		8.5	0.75 (3H, dd, 7.2, 2.9)
19	20.9	0.88 (3H, d, 6.8)		20.9	0.88 (3H, d, 6.8)
20	51.7	3.62 (3H, s)		51.7	3.62 (3H, s)
1'	96.9^d	5.12 (1H, d, 3.6)		92.1^d	4.52 (1H, m)
2'	75.1	3.17 (1H, m)		75.1	3.17 (1H, m)
3'	71.0	3.45 (1H, m)		71.0	3.45 (1H, m)
4'	74.3	4.76 (1H, m)		74.3	4.76 (1H, m)
5'	69.5	3.63 (1H, m)		69.5	3.63 (1H, m)
6'	60.8	3.46 (1H, m)		60.8	3.46 (1H, m)

$^{a–d}$ Interchangeable.

Compound **5** readily epimerizes at the anomeric C-1’ to give an equilibrium mixture of epimers (**5a** and **5b**) in DMSO-*d*$_6$. Therefore, the structure elucidation was carried out using the spectroscopic data for the mixture of **5a** and **5b**.

Table S8. ^{1}H (500 MHz) and ^{13}C NMR (125 MHz) for **7** in DMSO-*d*$_6$.

colorless gum; $[\alpha]^{22}_{D}$ + 44 (*c* 0.1, MeOH). HRESIMS *m/z* 685.3045 $[M+Na]^+$ (calcd for $C_{31}H_{50}NaO_{15}$, 685.3042).

selected 2D NMR correlation

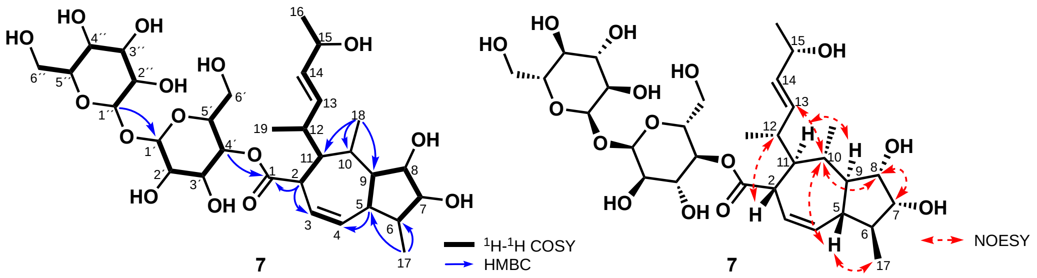

	**7**	
position	^{13}C	^{1}H (*J* in Hz)
1	173.8	–
2	49.6	3.19 (1H, m)
3	131.9	5.46 (1H, d, 12.3)
4	132.6	5.61 (1H, d, 12.3)
5	41.5	1.48 (1H, m)
6	45.7	1.37 (1H, m)
7	76.5	3.46 (1H, m)
8	71.9	3.18 (1H, m)
9	55.7	1.48 (1H, m)
10	30.3	2.27 (1H, d, 7.7)
11	50.2	1.86 (1H, t, 8.2)
12	35.2	2.61 (1H, q, 7.5)
13	132.7	5.22 (1H, m)
14	134.5	5.22 (1H, m)
15	62.4	4.43 (1H, p, 6.0)
16	17.1	1.04 (3H, m)
17	24.7	1.04 (3H, m)
18	9.1	0.73 (3H, dd, 7.1, 3.6)
19	21.0	0.88 (3H, d, 6.0)
1′	93.2	4.87 (1H, d, 3.9)
2′	71.6	3.46 (1H, m)
3′	70.1	3.76 (1H, dd, 10.9, 7.8)
4′	71.5	4.72 (1H, td,9.9, 9.9, 3.3)
5′	70.2	3.89 (1H, dd, 10.9, 4.9)
6′	60.3	3.46 (1H, m)
		3.18 (1H, m)
1′′	93.6	4.92 (1H, d, 4.2)
2′′	71.6	3.18 (1H, m)
3′′	72.9	3.46 (1H, m)
4′′	70.0	3.18 (1H, m)
5′′	72.7	3.66 (1H, m)
6′′	60.8	3.46 (1H, m)

Table S9. ^{1}H (500 MHz) and ^{13}C NMR (125 MHz) for **7a** in DMSO-d_6.

white power; $[\alpha]^{22}_{D}$ – 66 (*c* 0.1, MeOH). HRESIMS *m/z* 375.2146 $[M+Na]^+$ (calcd for $C_{20}H_{32}NaO_5$, 375.2142).

selected 2D NMR correlation

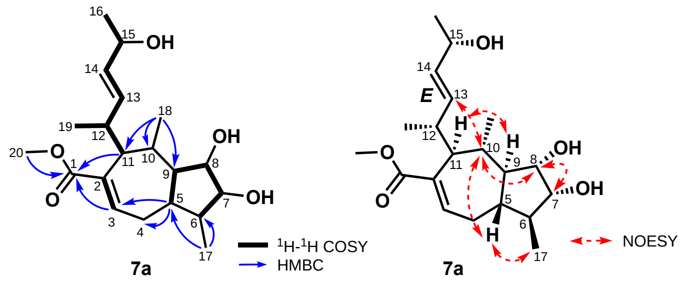

	7a	
position	^{13}C	^{1}H (*J* in Hz)
1	168.4	–
2	136.5	–
3	141.7	6.80 (1H, ddt,9.1, 4.7, 2.4, 2.4)
4	30.1	2.28 (1H, m)
		1.99 (1H, m)
5	35.6	0.63 (1H, m)
6	45.9	1.28 (1H, m)
7	75.5	3.21 (1H, s)
8	71.2	3.38 (1H, dd, 9.6,7.0)
9	59.9	1.61 (1H, tdd, 14.9, 8.7, 3.9)
10	30.2	2.16 (1H, qt, 6.9, 3.2)
11	50.6	2.28 (1H, m)
12	30.9	3.25 (1H, dtd, 9.6, 7.0)
13	133.9	5.02 (1H, td, 10.6, 1.3)
14	135.0	5.36 (1H, m)
15	63.0	4.60 (1H, m)
16	25.0	1.10 (3H, t, 5.9)
17	16.8	0.95 (3H, d, 6.8)
18	8.8	0.63 (3H, m)
19	20.6	0.63 (3H, m)
20	51.4	3.78 (3H, d, 4.4)

Table S10. Homology (% identity) of *tre* cluster and genes from the plant pathogens.

Biopolaris sorokiniana	ThT22	*Zymoseptoria tritici*	*Cercospora zeae-maydis*	*Fulvia fulva*
TreA	52.85%	58.73%	64.31%	60.50%
TreB	46.00%	51.26%	58.14%	55.56%
TreC	63.16%	67.66%	66.67%	67.42%
TreD	33.87%	45.94%	52.72%	46.90%
TreE	50.67%	52.87%	58.59%	61.57%

Supplementary Figures

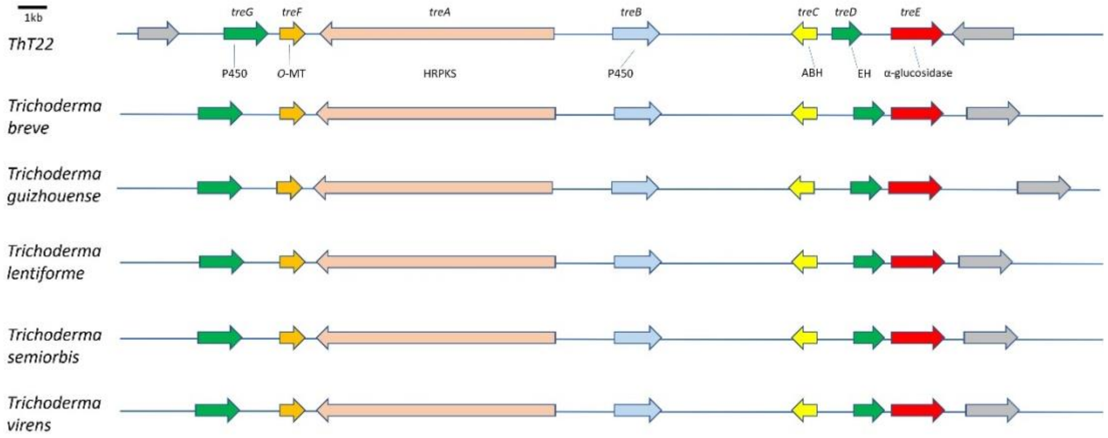

Figure S1. Homologous gene clusters in other *Trichoderma sp.*

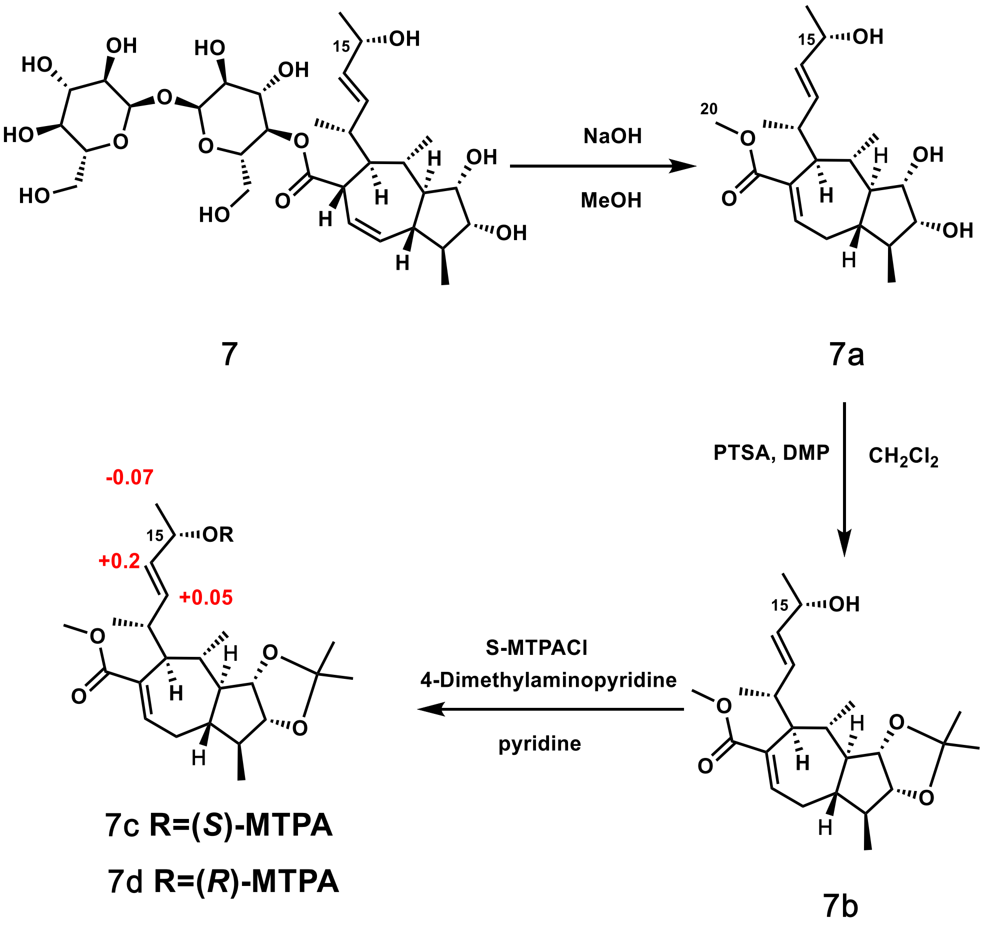

Figure S2. Mosher derivatization to determine the absolute configuration of C-15 in **7**. The absolute stereochemistry of C-15 was determined to be *S*.

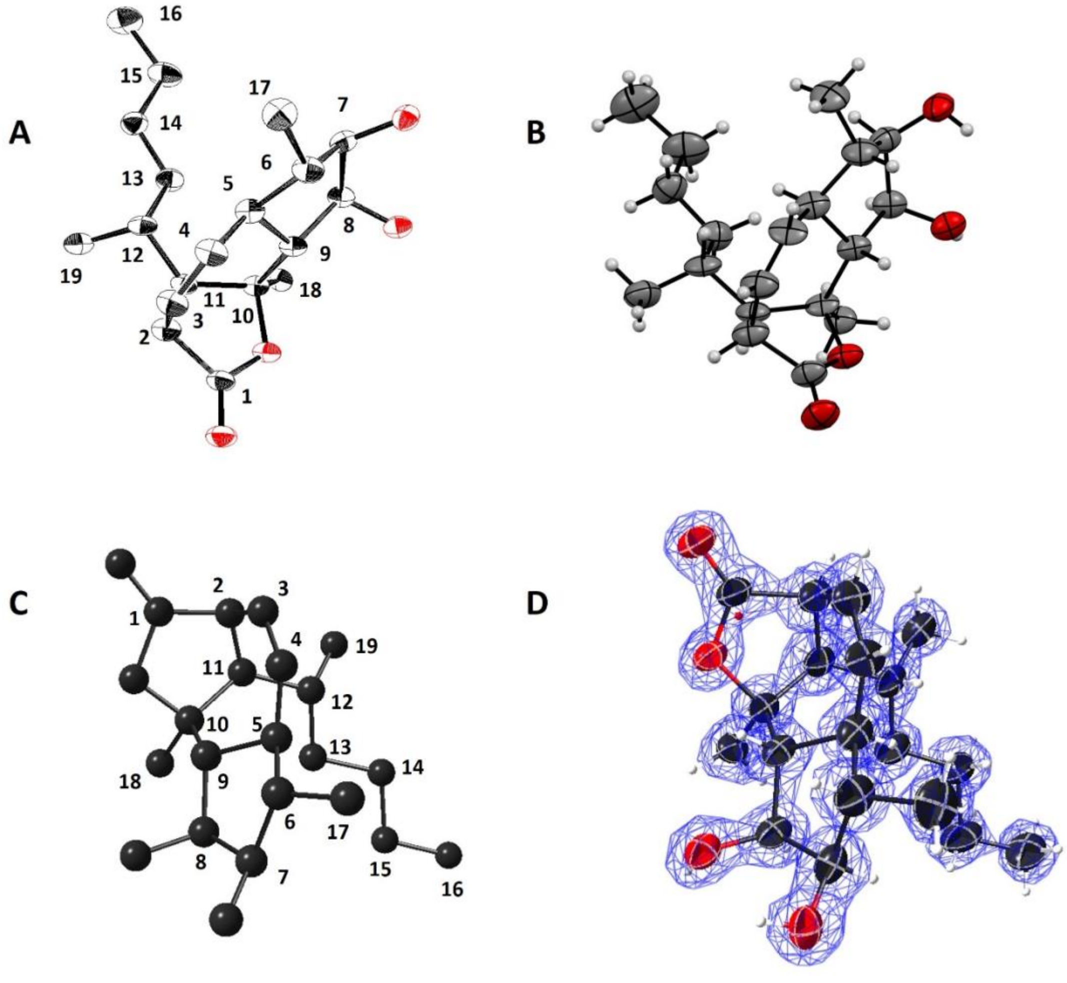

Figure S3. (A) and (B) ORTEP diagram of compound **3** structurally characterized from MicroED**. (**CDCC 2295175). (C) Initial direct methods solution of **3**. (D) Electron potential map of refined **3**.

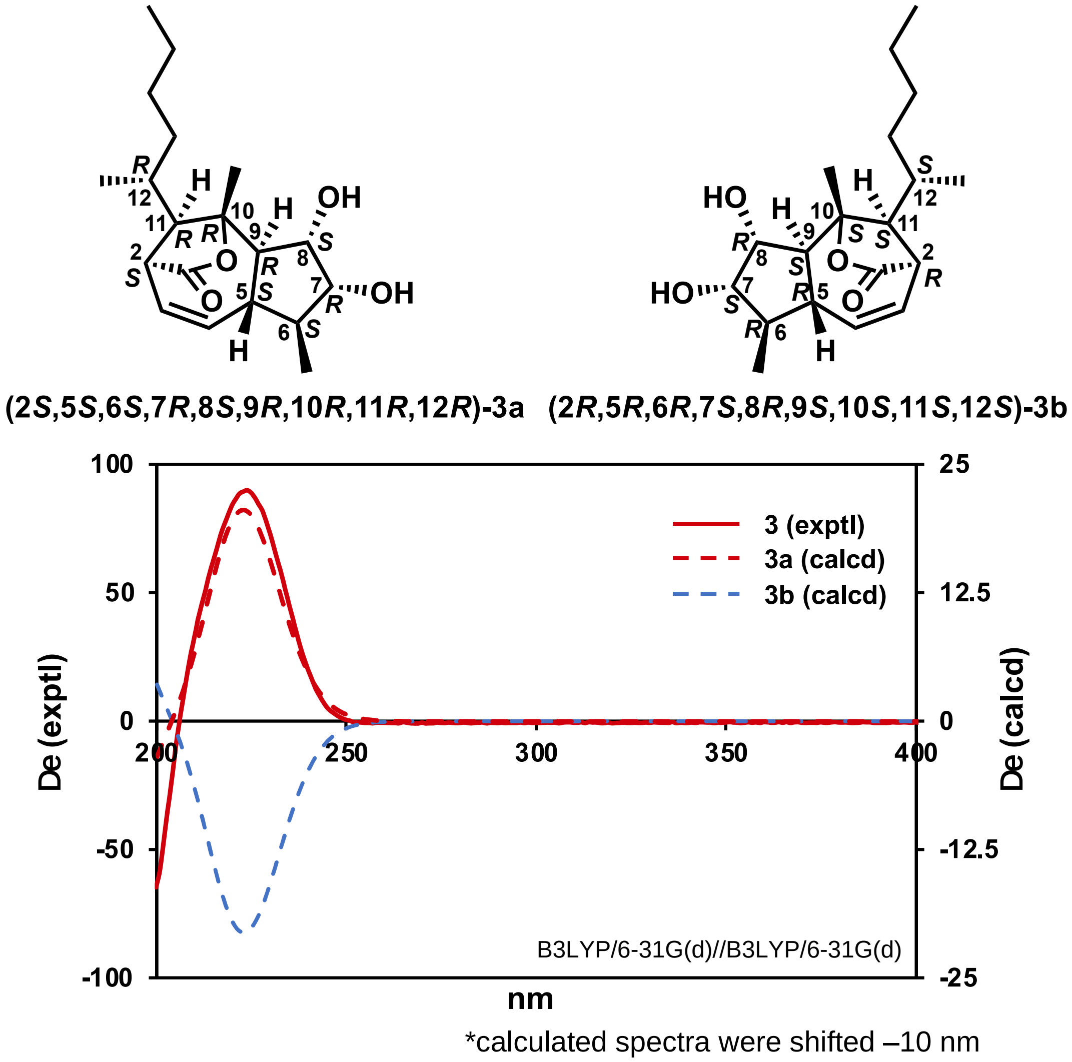

Figure S4. Experimental ECD spectrum of compound **3** and calculated spectra of (2*S*,5*S*,6*S*,7*R*,8*S*,9*R*,10*R*,11*R*,12*R*)-**3a** and (2*R*,5*R*,6*R*,7*S*,8*R*,9*S*,10*S*,11*S*,12*S*)-**3b**. The absolute stereochemistry of **3a** matches the configuration of citrinovirin reported in Ref 22.

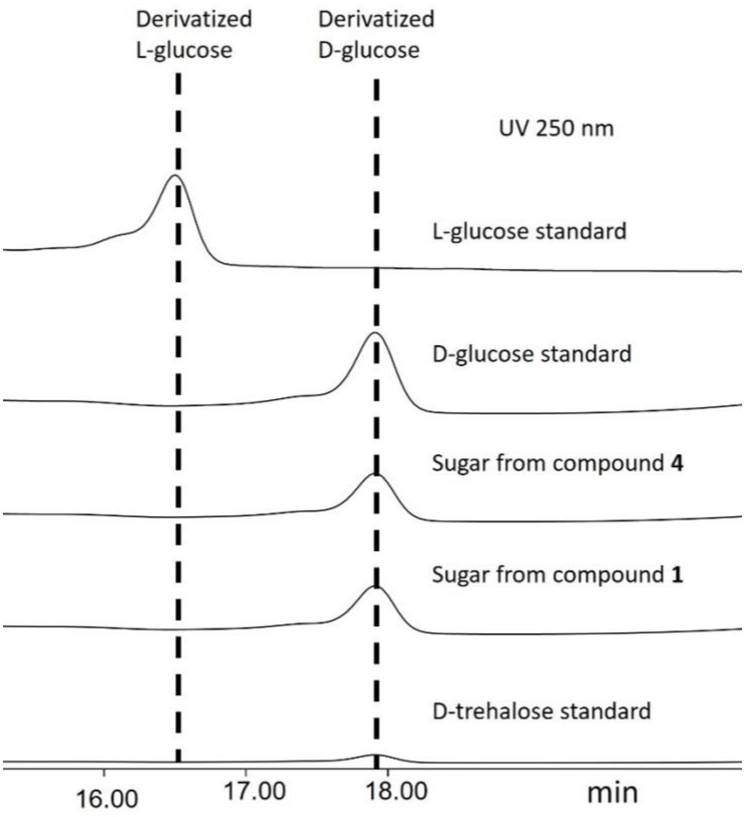

Figure S5. Determination of the identity of the sugar ester. The results showed that the sugar moieties from the compounds isolated are D-trehalose and D-glucose. D-trehalose can be further hydrolyzed into D-glucose.

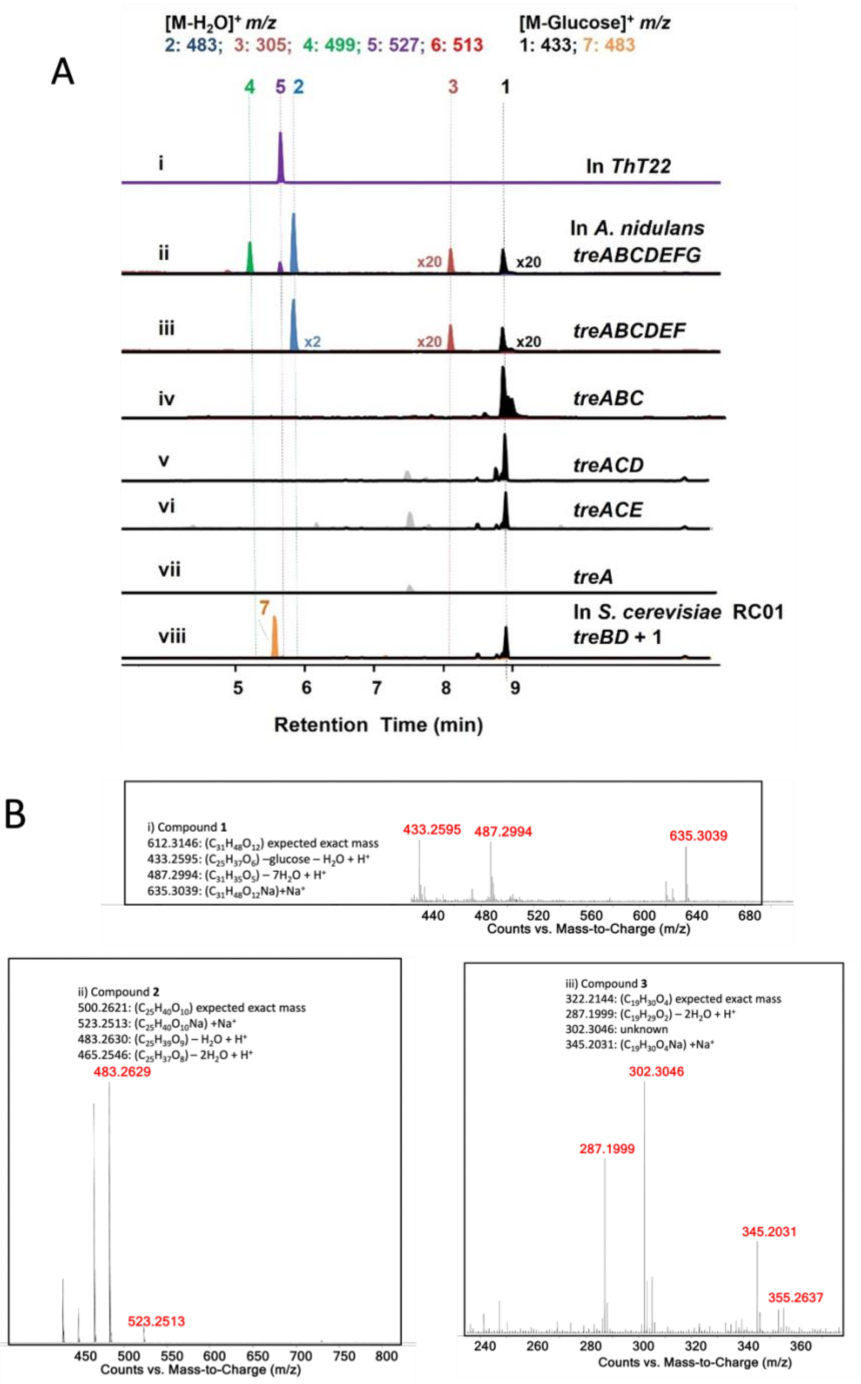
A
[M-H2O]+ m/z
2: 483; 3: 305; 4: 499; 5: 527; 6: 513
[M-Glucose]+ m/z
1: 433; 7: 483
i In ThT22
ii In A. nidulans treABCDEFG
iii treABCDEF
iv treABC
v treACD
vi treACE
vii treA
viii In S. cerevisiae RC01 treBD + 1
Retention Time (min)
B
i) Compound 1
612.3146: (C31H48O12) expected exact mass
433.2595: (C25H37O6) –glucose – H2O + H+
487.2994: (C31H35O5) – 7H2O + H+
635.3039: (C31H48O12Na)+Na+
ii) Compound 2
500.2621: (C25H40O10) expected exact mass
523.2513: (C25H40O10Na) +Na+
483.2630: (C25H39O9) – H2O + H+
465.2546: (C25H37O8) – 2H2O + H+
iii) Compound 3
322.2144: (C19H30O4) expected exact mass
287.1999: (C19H29O2) – 2H2O + H+
302.3046: unknown
345.2031: (C19H30O4Na) +Na+
Counts vs. Mass-to-Charge (m/z)

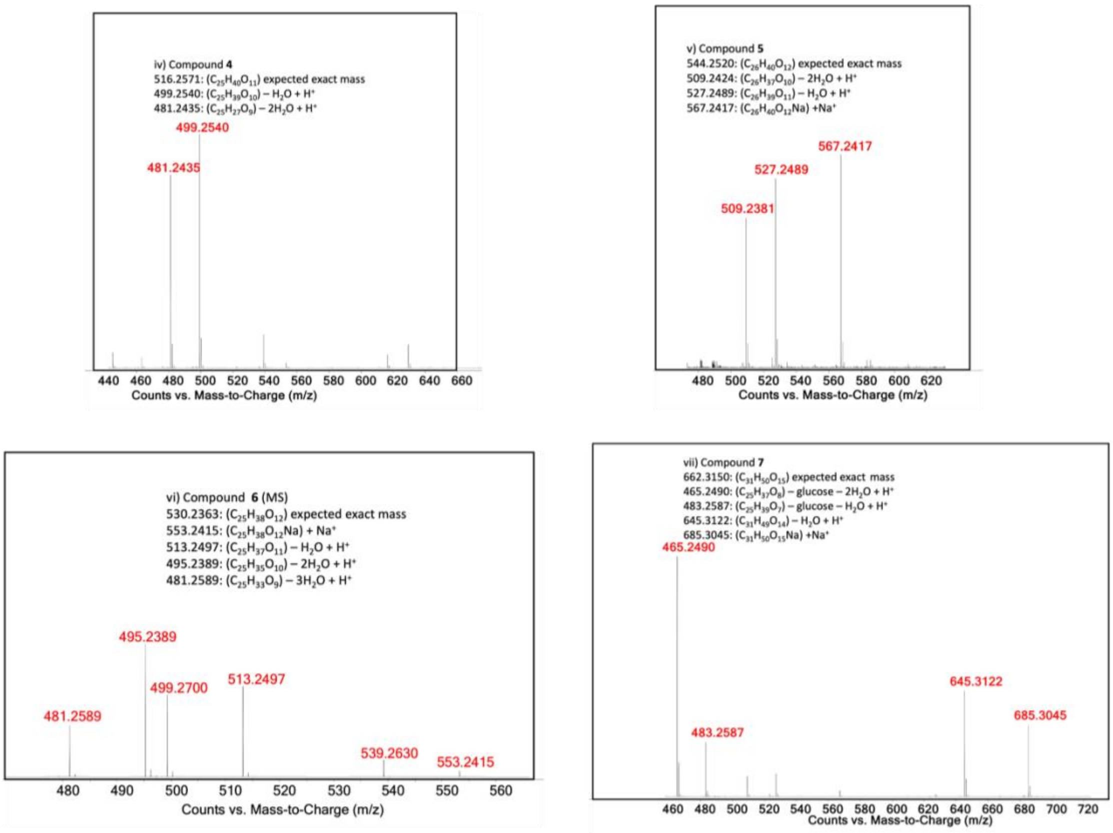

Figure S6. (A) Heterologous expression in *A. nidulans* and feeding assay in *S. cerevisiae*. Individually coexpression of TreB (iv), TreD (v), or TreE (vi) with TreAC did not lead to any new product. (B) MS data for compounds **1-7** . Compound **6** overlaps with compound **4** (Figure 2b iii) under LC conditions, results in two extra signals (499.2700, 539.2630) from compound **4**.

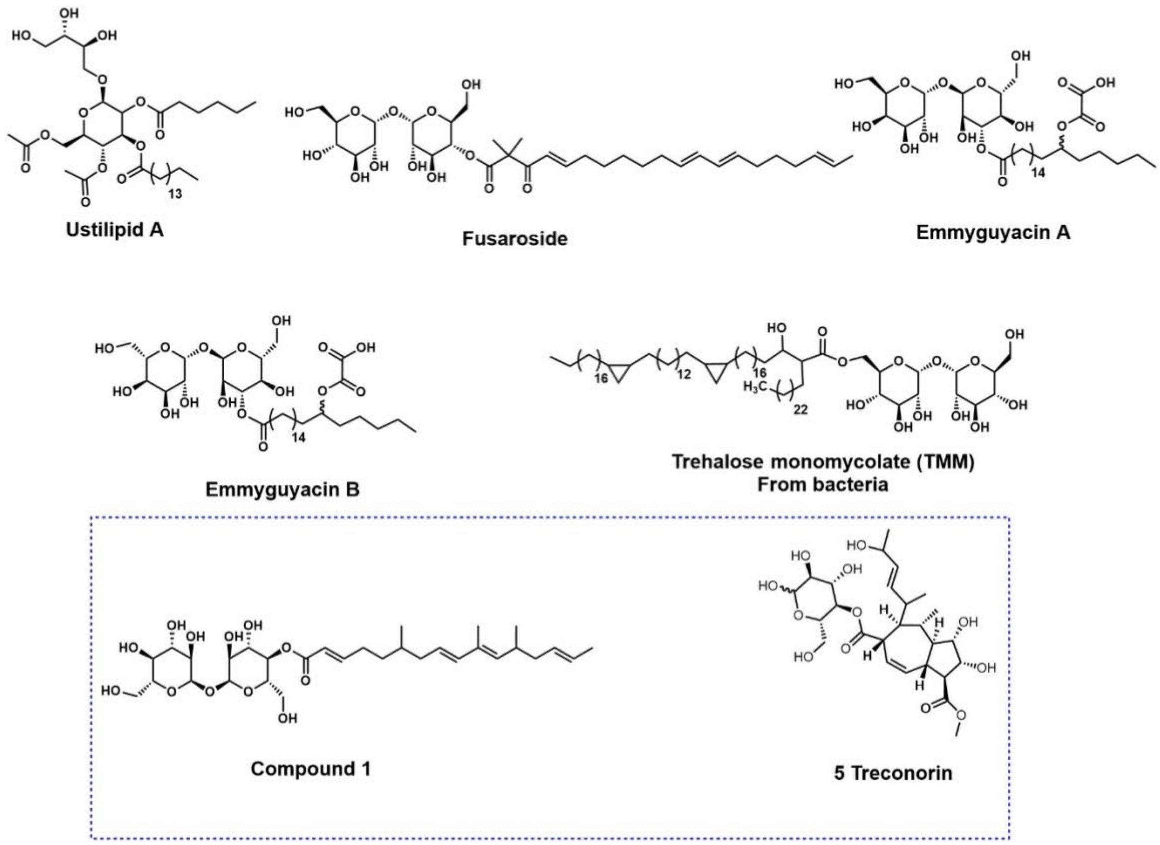

Figure S7. Examples of reported trehalose lipids and glycoesters.

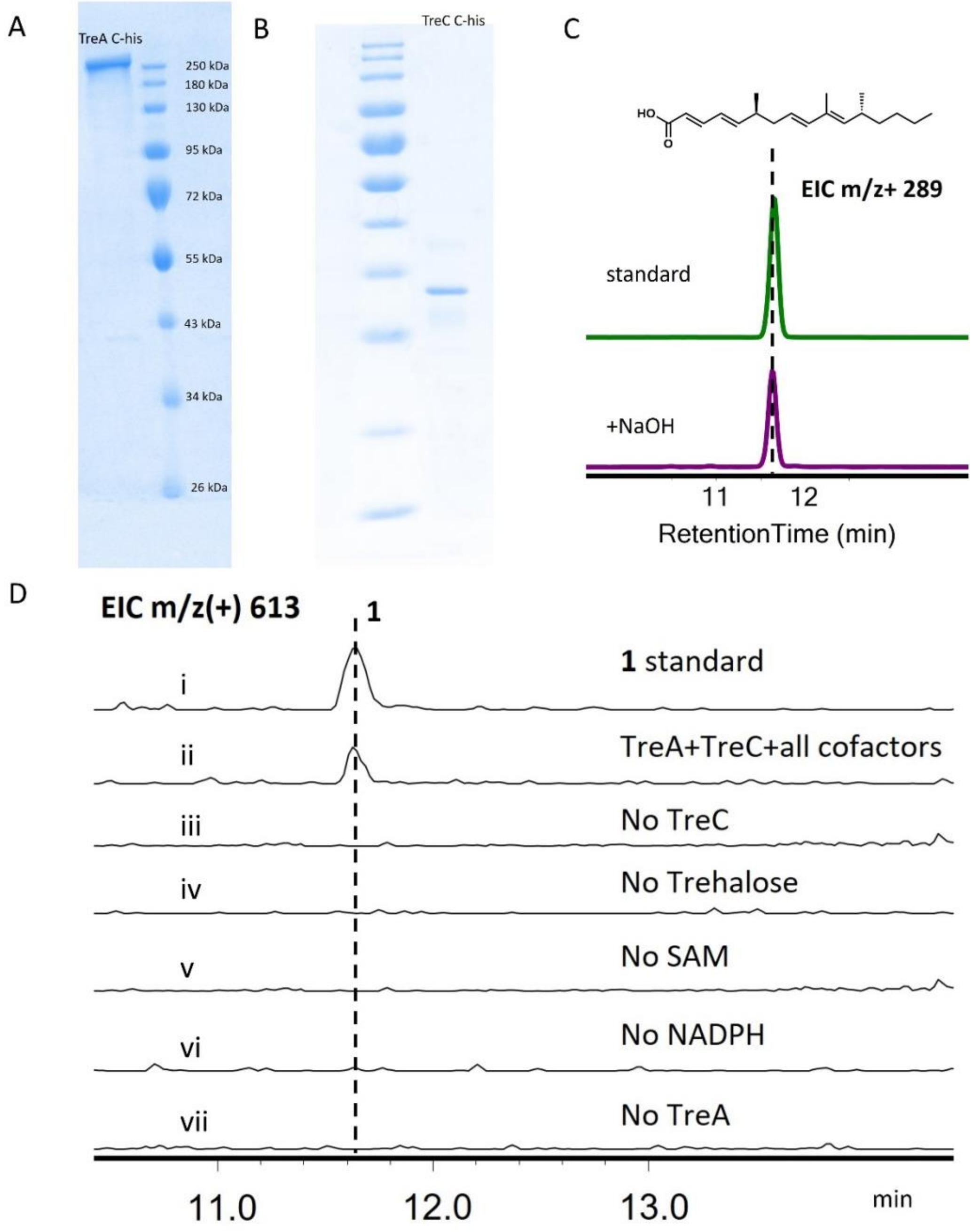

Figure S8. *In vitro* assays of TreA and TreC. (A) SDS-PAGE gel of purified TreA. (B) SDS-PAGE gel of purified TreC. (C) Top: standard of **1** purified from *A. nidulans*. Bottom: *in vitro* assay in which only TreA was mixed with all cofactors. After 12 h, the mixture was treated with NaOH to hydrolyze the polyketide product. (D) *In vitro* assays of TreA and TreC with different combinations of substrate or cofactors. The results confirmed TreC uses trehalose as nucleophile to release the polyketide product from TreA**.**

A

```
                         .      .                . ..
ThT22_TC    GRFPSVDMQLKGPSIFQLLSFENWTYPDATLAGFYFIGSFGVSWALIVLESLRSYNL--H    115
atma        TGLKAFDGILESIVIFFW-PISQGHHVGLSLTGLSFSGGMVGIWMIVVVHICRIR--SFT    111
penA        TGLDPLDKFLKACNVFFW-PIFHGTSPALSLYAIAFAGSMIPMWLILLMHTCVKS--SIV    110
paxA        TGIRPLDTFLTSCTVFFW-PTFQGEIPGLSLYGIAFASAMIPMWLIIVIDVHRRRQ-PFG    111
AscI        TGLEGLDRLLRTLLNFFW-PVANGHDWALSLHAFMFAGQGVPLLVLNMLEGARPGNKSL-    112
aurD        TGIASLDETLAAMFVFYW-PVLDGSFPGLSLMFCNYLGALPLCLVLMTLESLRKGNRSSF    113
ctvD        ITGTGLDELLGNLIVFYW-PVLDGNHPGLSLQAFHFLGAIVAVWVAIQVQSWRSPNRNS-    109
                 .*  *     *      .      :*    : .          :.

ThT22_TC    NFGSWTTIPGLLMFNHTPAIYLPLYLGVRLALMT-P--SNLTPSDLAIDPVQLELFPWAF    172
atma        RGMVITLIVGIAQQAVGPGIVIPCYFALTS----RARPPNKNLHLTGTYSTSNHGLVVSM    167
penA        EIVMINALTGLLVQGIGPGVMMCVLLAMRS----TSMEEFAVTSIPAVSILGPNDLPLSL    166
paxA        ALVELIAFAGPLIQCIGPGLVMPLLLARIH----TPSRDSKS-----ASQFDYRTFIPSM    162
AscI        -VVSYVTVFGILYMVVGLAIMAPLYLFLHLLTSRTATAPSKA--KVAVDPNTAKAVGFGV    169
aurD        SFFYSPTFWGMIAVMMTLAVSIPWYLTIHLLISTTASHPTIE--NMSIPLAELKALIVNI    171
ctvD        -LLRSPTLFAMLSQVVAIAVIVPLWCAISIWSSSSPRPITRA--VSASAAHSIRLIPISM    166
                   . .        .:                                 . .   .

ThT22_TC    AIGYVAPFIAMLLPDLRIRDISTKQTVAAWWQQWPAYVALSQLLLSLIWRPAVLP---SP    229
atma        IMSYIFPLVIMSLPAPAMISPHSKQQVIAAWQGWPVYFVIIMTTHHLFINRGHRK-----    222
penA        VVCYILPLALSSLPAPASISVPSKQLFIASWQGWPLYIALAVGIAHSL-RYGY-R-----    219
paxA        IIGYILPLLLASLPAPLILSYHNKQQFIAIWQGWPLYSSVLMWA-FRR-RSGH-V-----    214
AscI        FVGYVLPTIFMSLPHPSLLSTDTKVLSVVFWQAVPLWASVCAYFASTALGQSAT----SR    225
aurD        VVGLVLPSLLVALPETITQTLFTRQTAITLWQLWPFWSTAVHFIARKFISATERGADSRA    231
ctvD        VLGFGIPTIGMLLPESTHQNLFSKQIAIAVWQIWPIYVALWHWGLRVLFRSRLKE-----    221
             :    *     **        .:    . **  * :

ThT22_TC    LATEAWPQVQSVYMFLFVLAALSHWLSVFGAFAT-----------------GVTLTELFL    272
atma        -EASARRQVLSVYHFGFACSCLCHMAWL-----SAFVASKIQSLSQSSNFWYLCPYGVAF    276
penA        -RSRPQQLFRHAYAFALACSIISHVGLLLISFLSIYPKSPFLSLHSA----DLHPQSLLV    274
paxA        -HCSRHKGLKHACIFALACSSAGHLVLLSLTWLWSLSY------------------WGYI    255
AscI        SSSNLPSALGAVYAASLIIATATHVATFAISANLSDTWSG--------IFTFLIPPNPF-    276
aurD        QWTRVRSAFRSVYGLTFAAAAIAHIATWSISLTAAYALPDA---MSAETVSSLHPQTVFV    288
ctvD        -GISVRTACRTACSFAFVCAIIPHAVSWGLSLTL-------------------IPTNLLA    261
                       .    :  :   *

ThT22_TC    PAWPGKD-----KKARHGWEAAKWCVQWDVVLDTPAMILWAGVLYWQTGGVMDS------    321
atma        PLLNQPA-----QRLGALEAGLFTFLQWDYCVAAAATMVWSTDRYIQECHRAE--LEIDK    329
penA        PRLPWQE-----VKITSLESGVLRFLHWDYSISSTGALLWCYDVYWEDRMRGK--GWIAF    327
paxA        QSAPWNE-----PPLASLEAGVLRFLQWDYTLSASATLSWAIAFRHEVVQQKS--LRISL    308
AscI        ---------NTDMRISSFLEGATWFLQWDYTMMSLAYMVWAIGIRHGVEVPRSSHHFETL    327
aurD        NTWPWLP-----VTTDSVGEGTLWLLQWDKFVGVGAIYWWSLDLYRAAHTAQRK--KINW    341
ctvD        DVLPWQFAGGGTVQVQSMAQGGLWFLQWDHLIGMGSFLLWAMHMRWTV-EQQSS--FLQT    318
                                .    ::**  :   .   *.
```

B

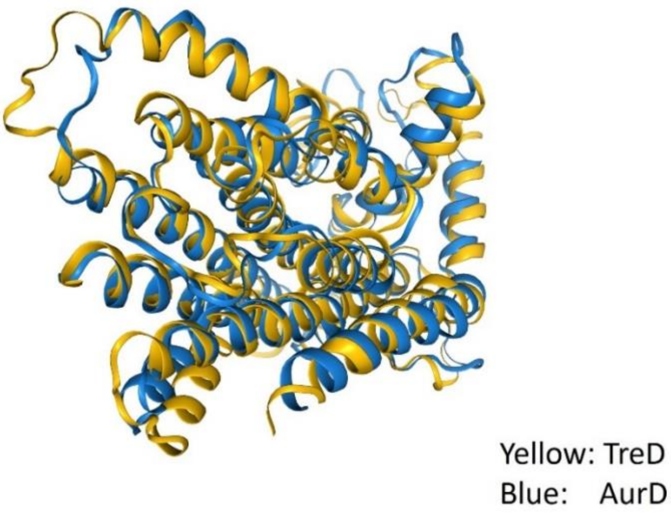

Figure S9. Structural and sequential alignment of TreD. (A) Clustal omega sequential alignment of TreD with other reported epoxide hydrolases. The highlighted residues are proposed active residues for catalytic function. (B) Structural alignment of alphafold2 predictions of TreD and AurD. Sequence identity between TreD and AurD: 20.7%

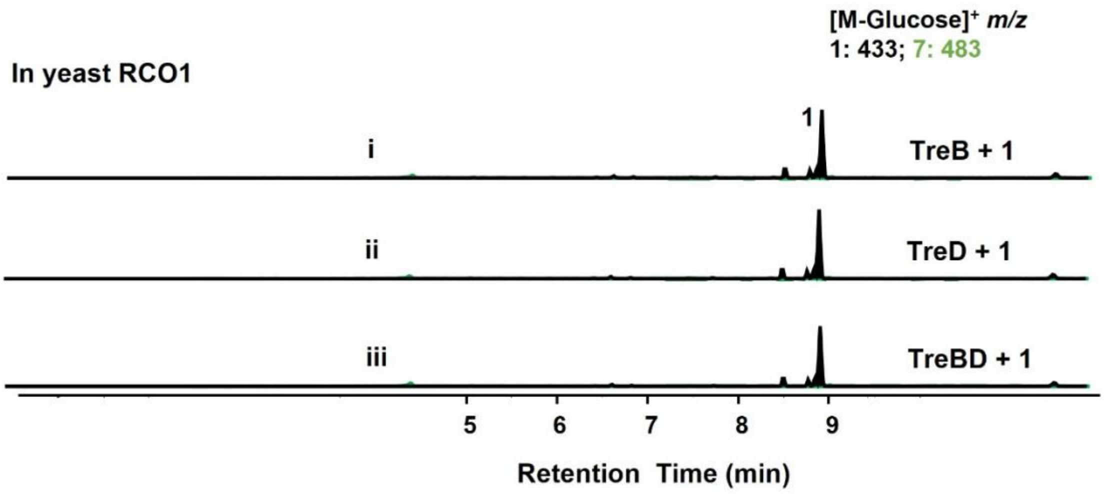

Figure S10. *In vtiro* assays using microsomal fractions containing TreB and TreD. Addition of **1** did not lead to any products.

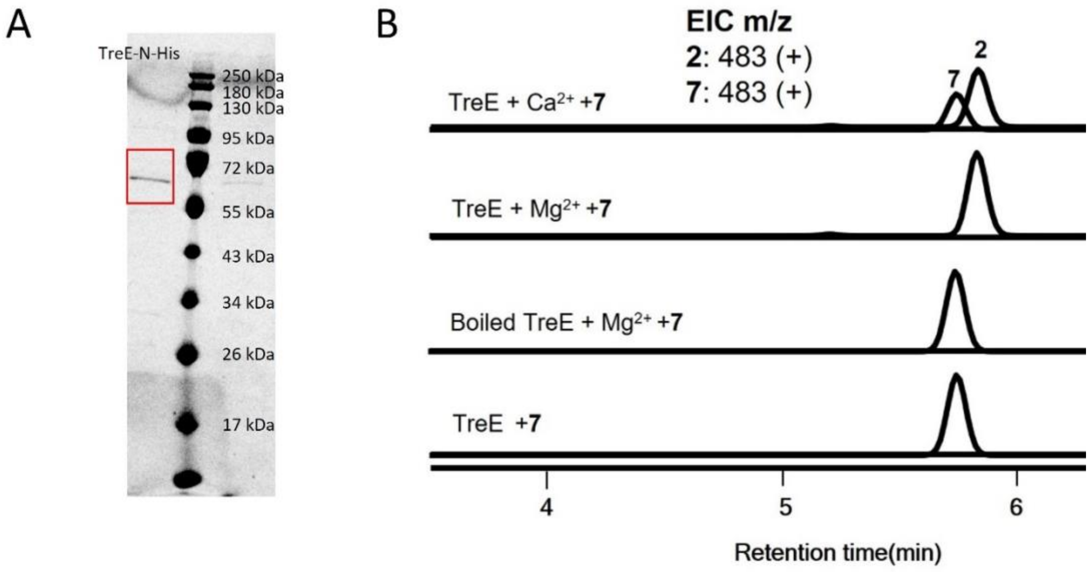

Figure S11. *In vitro* assays of recombinant TreE**.** (A) SDS-PAGE of the purified TreE expressed from *E. coli*. (B) *In vitro* assay with **7** as substrate. The results showed that TreE catalyzed the cleavage of disaccharide and converted **7** to **2**.

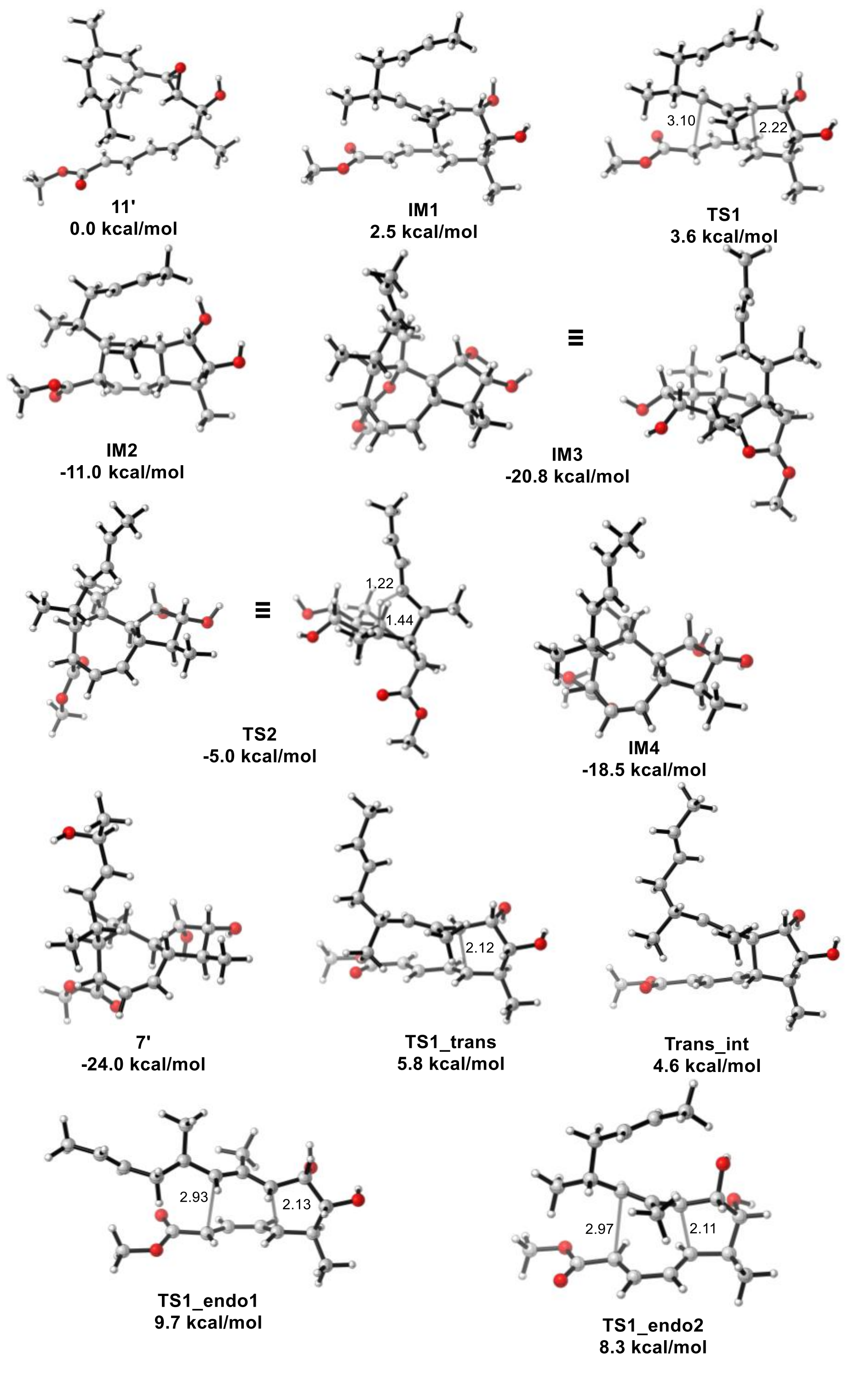

Figure S12. Geometries of compounds and transition states calculated by DFT. Bond distances are given in Å; Energies labeled are free energies calculated by ωB97X-D/def2-QZVPP/SMD(water)//ωB97X-D/def2-SVP/IEEPCM(water) with QRRHO correction**.**

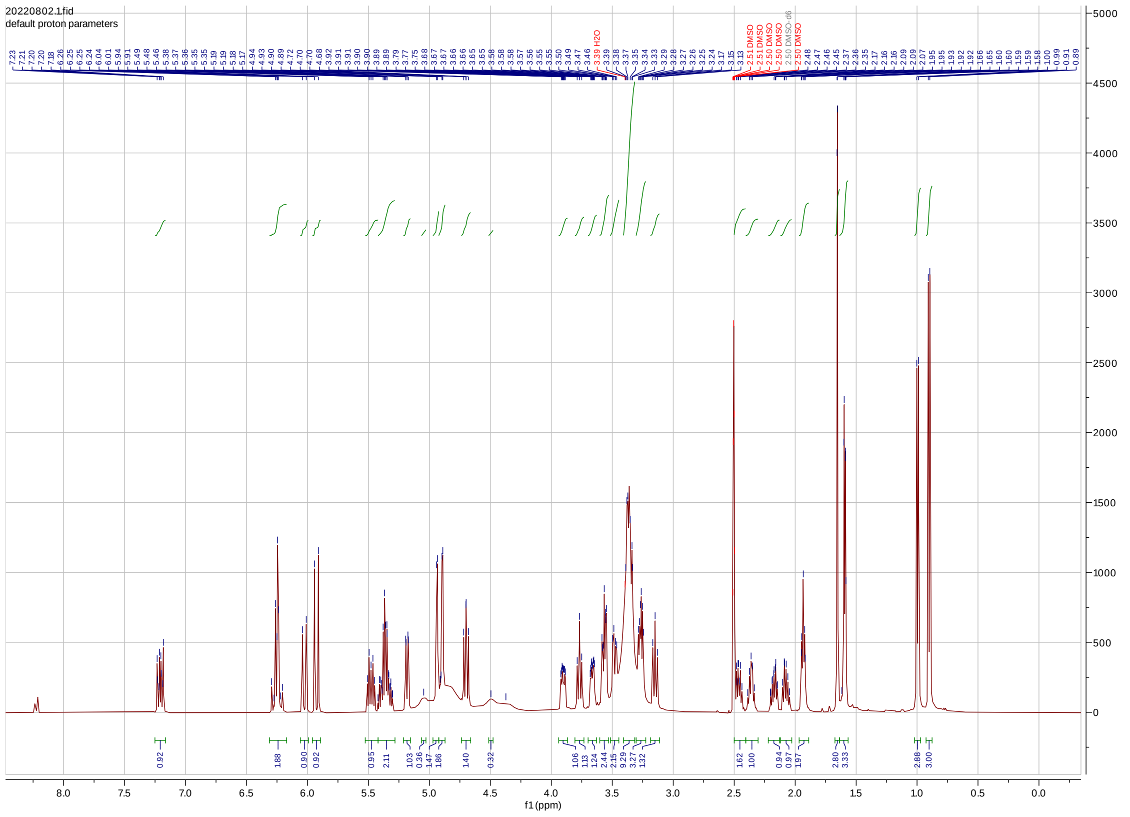

Figure S13. ^{1}H NMR spectrum of compound **1** in DMSO-d_6 (500 MHz)

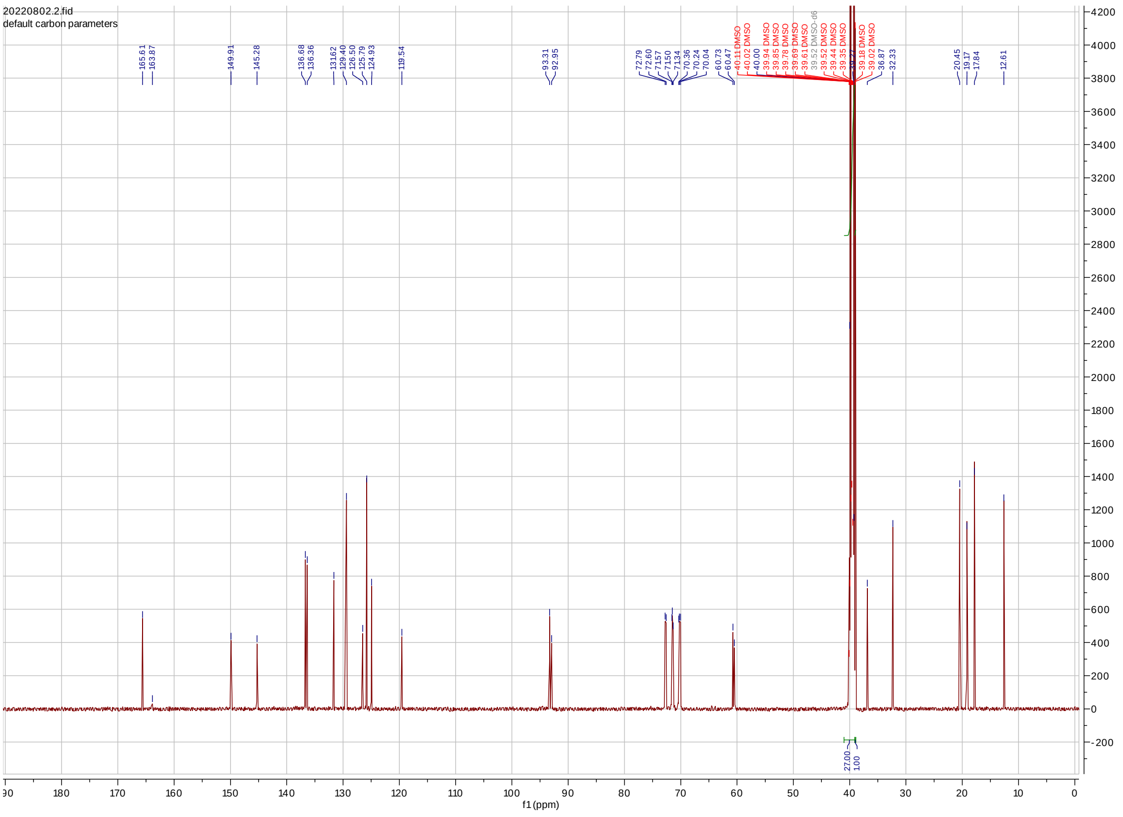

Figure S14. ^{13}C NMR spectrum of compound **1** in DMSO-d_6 (125 MHz)

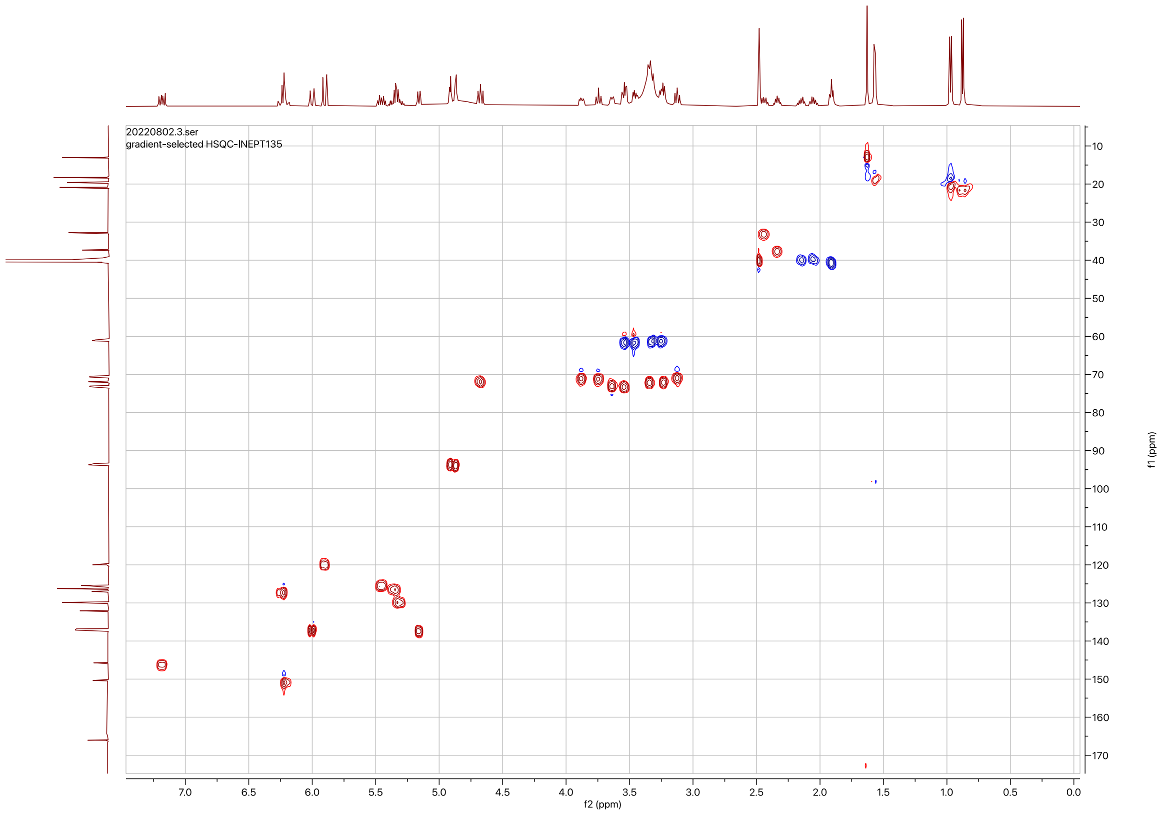

Figure S15. ^{1}H-^{13}C HSQC spectrum of compound **1** in DMSO-d_6 (500 MHz)

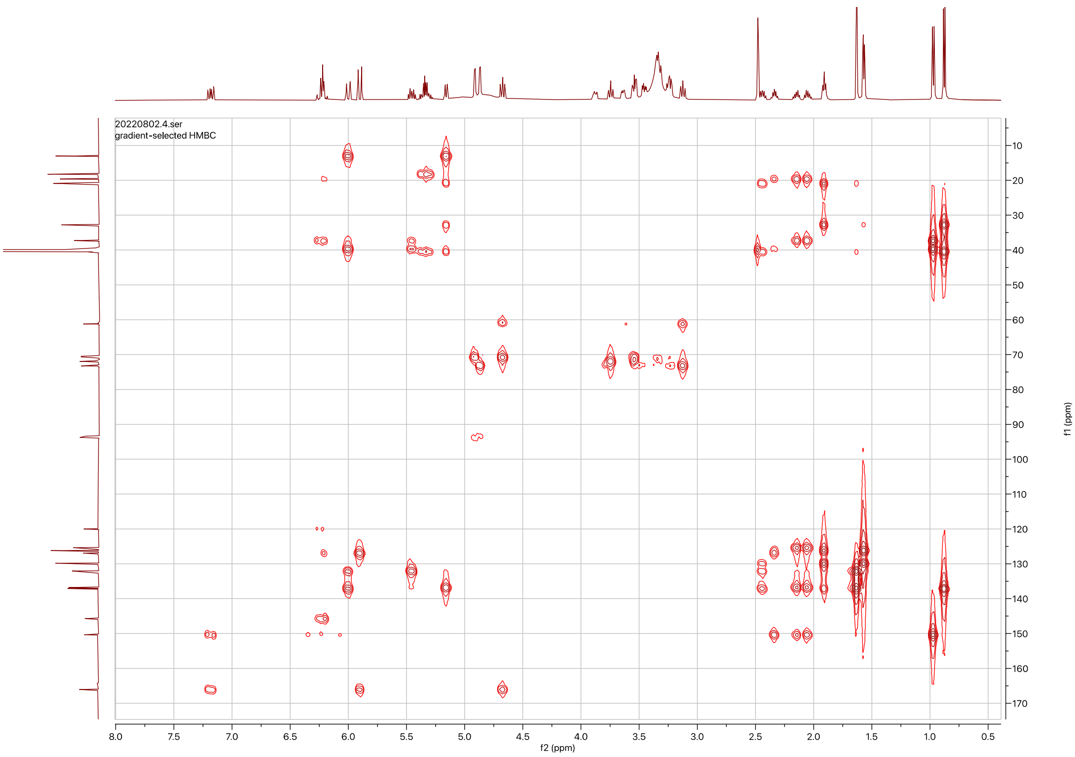

Figure S16. ^{1}H-^{13}C HMBC spectrum of compound **1** in DMSO-d_6 (500 MHz)

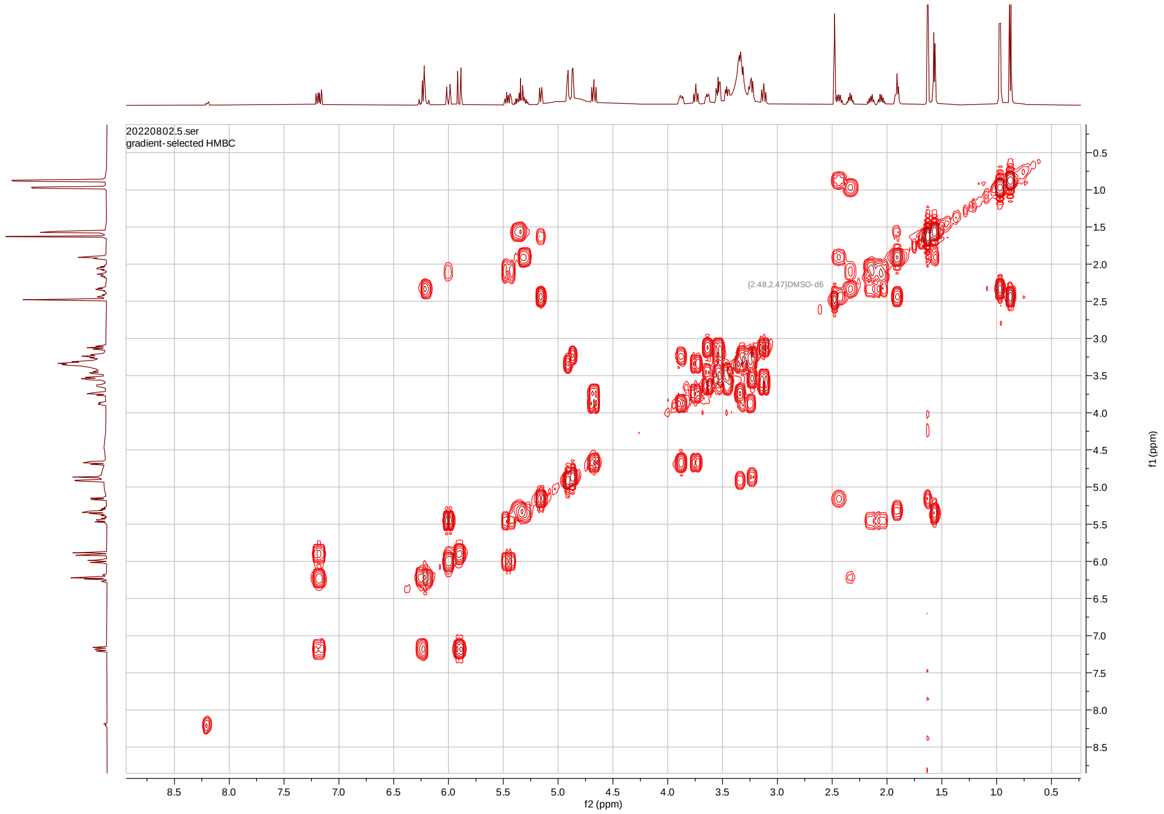

Figure S17. ^{1}H-^{1}H COSY spectrum of compound **1** in DMSO-d_6 (500 MHz)

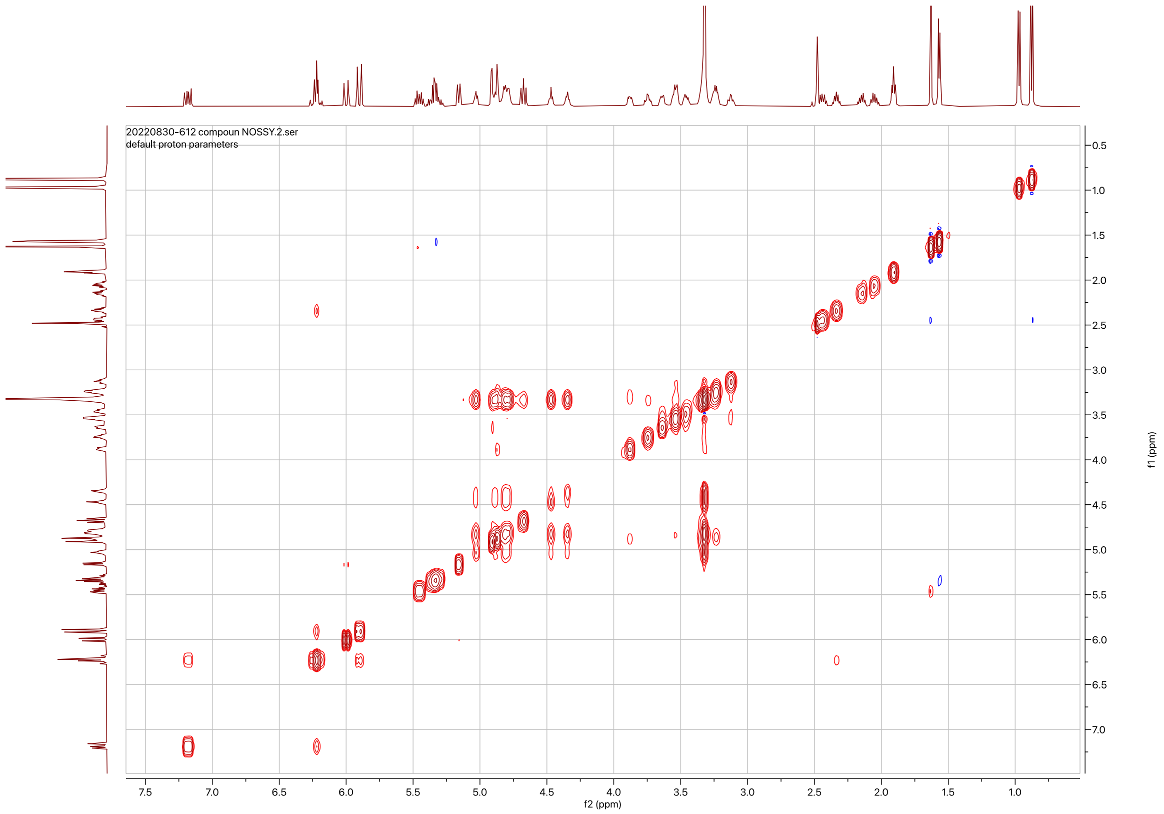

Figure S18. ^{1}H-^{1}H NOESY spectrum of compound **1** in DMSO-d_6 (500 MHz)

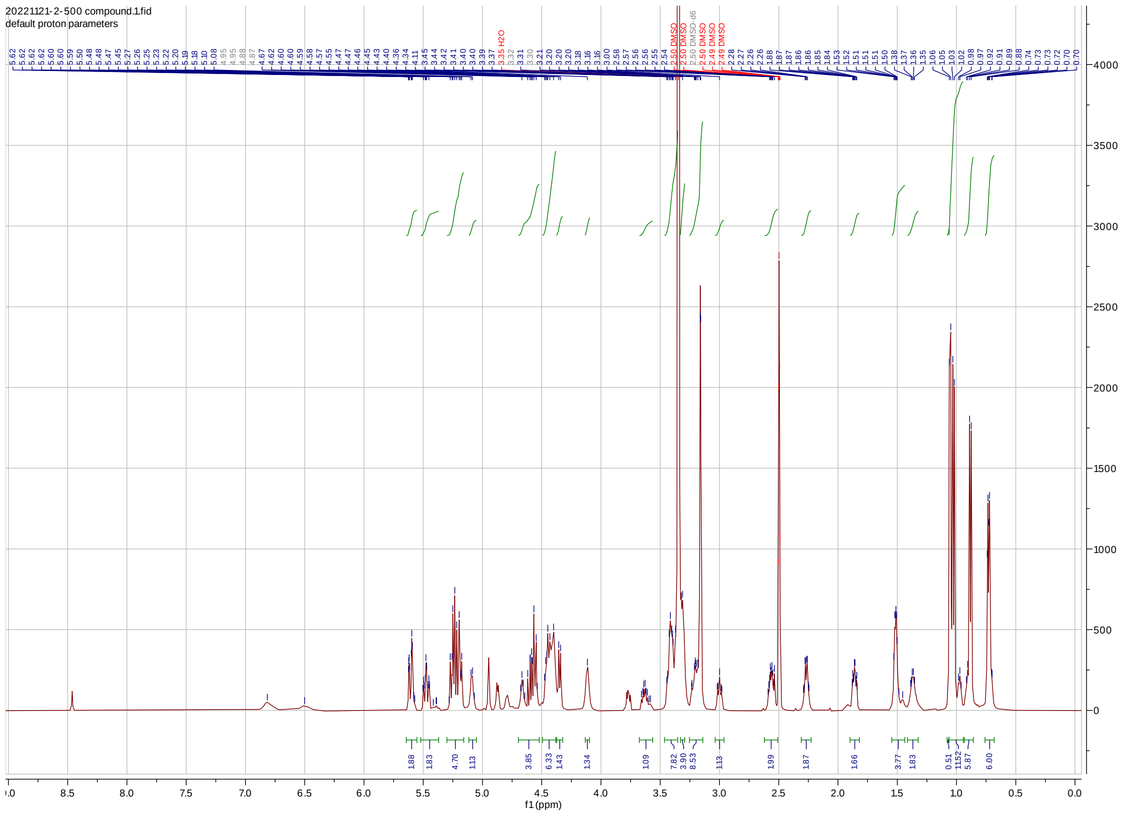

Figure S19. ^{1}H NMR spectrum of compound **2** in DMSO-d_6 (500 MHz)

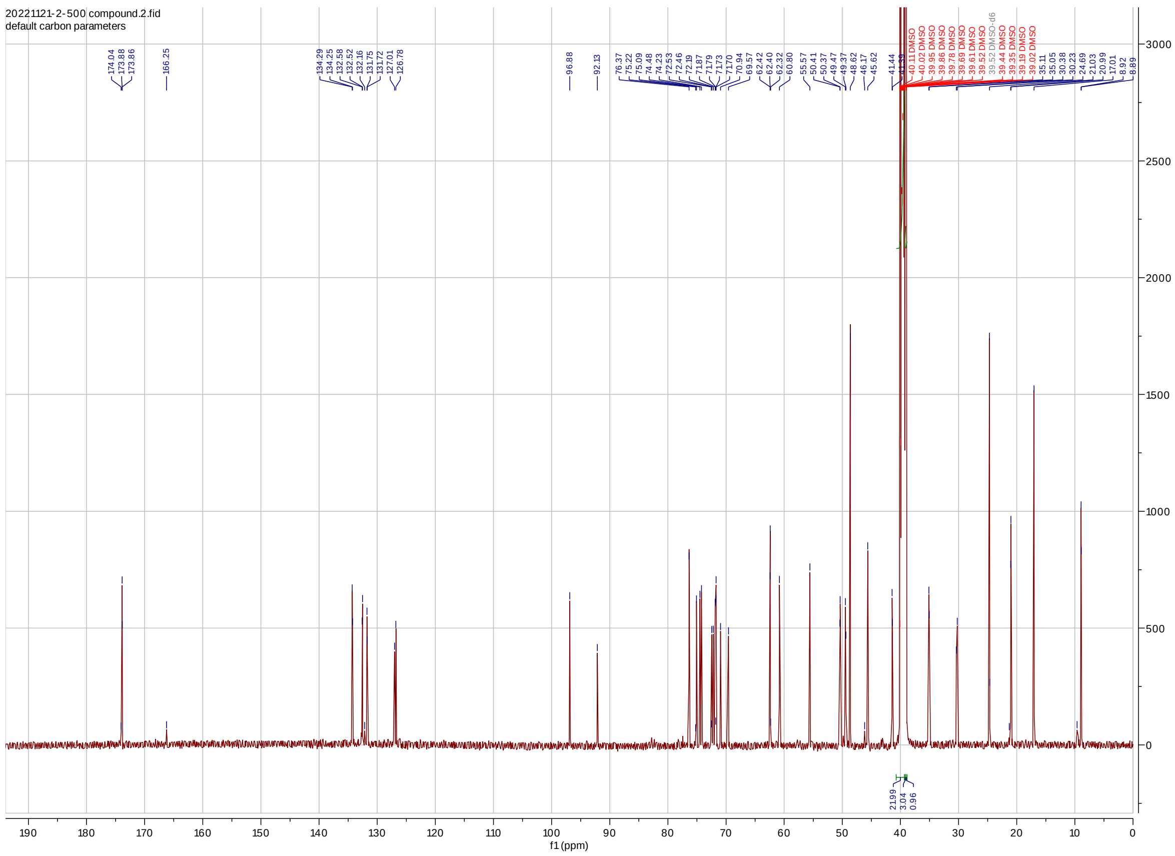

Figure S20. ^{13}C NMR spectrum of compound **2** in DMSO-d_6 (125 MHz)

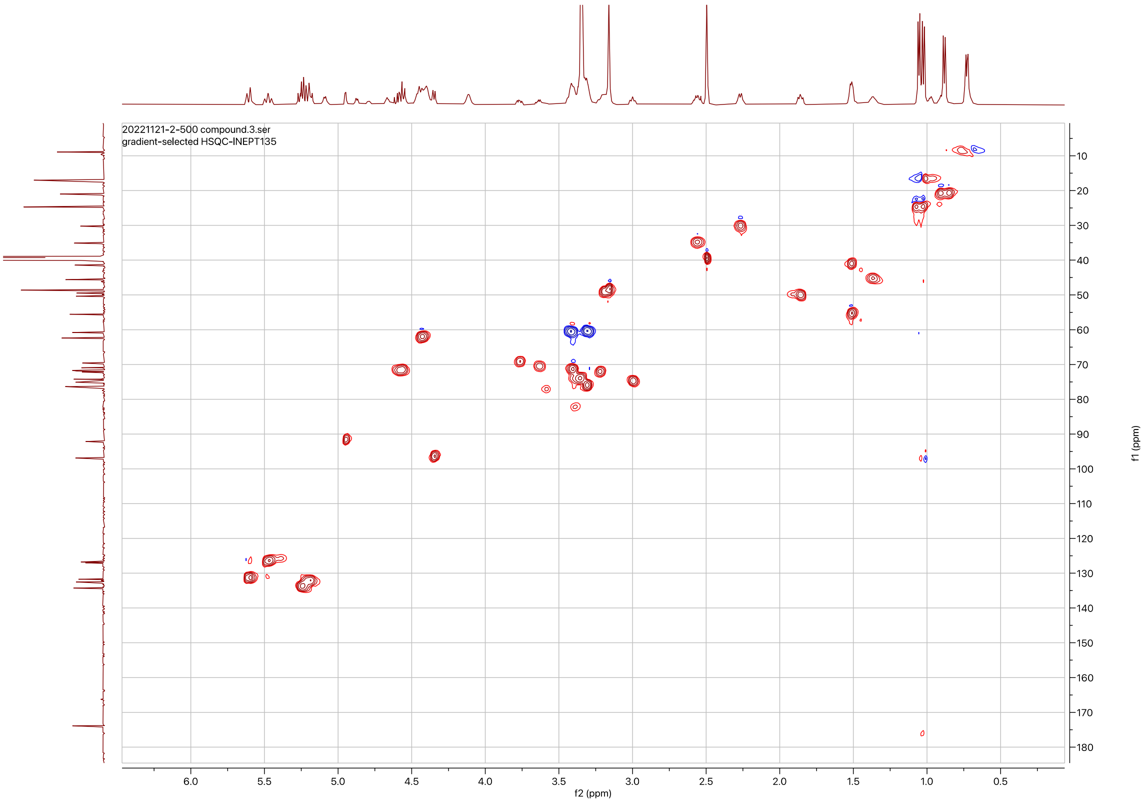

Figure S21. ^{1}H-^{13}C HSQC spectrum of compound **2** in DMSO-d_6 (500 MHz)

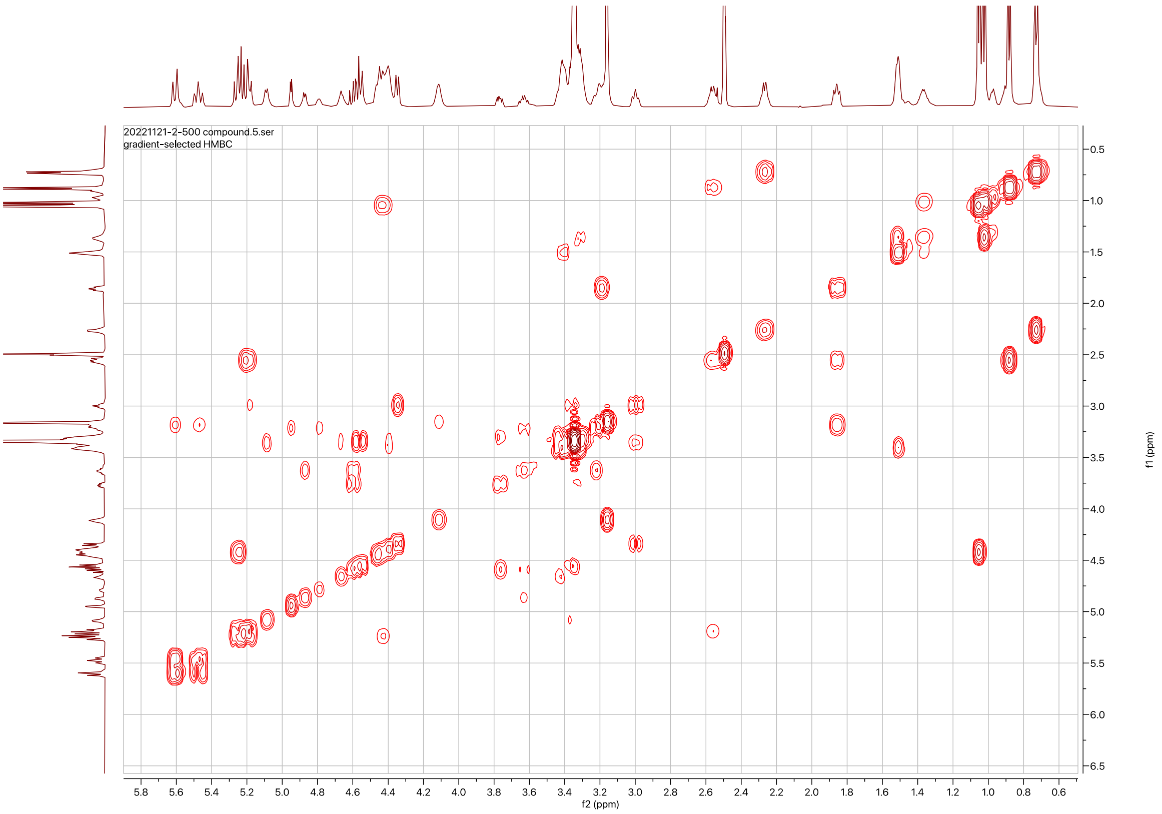

Figure S22. ^{1}H-^{1}H COSY spectrum of compound **2** in DMSO-d_6 (500 MHz)

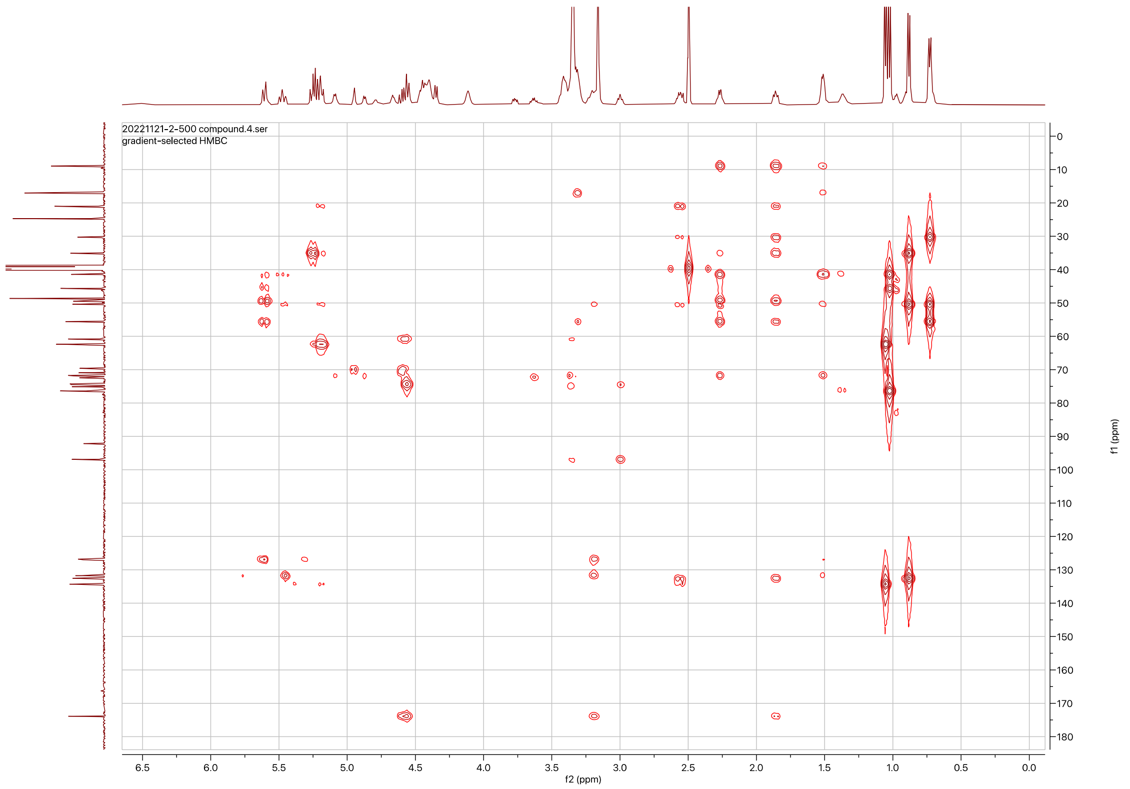

Figure S23. ^{1}H-^{13}C HMBC spectrum of compound **2** in DMSO-d_6 (500 MHz)

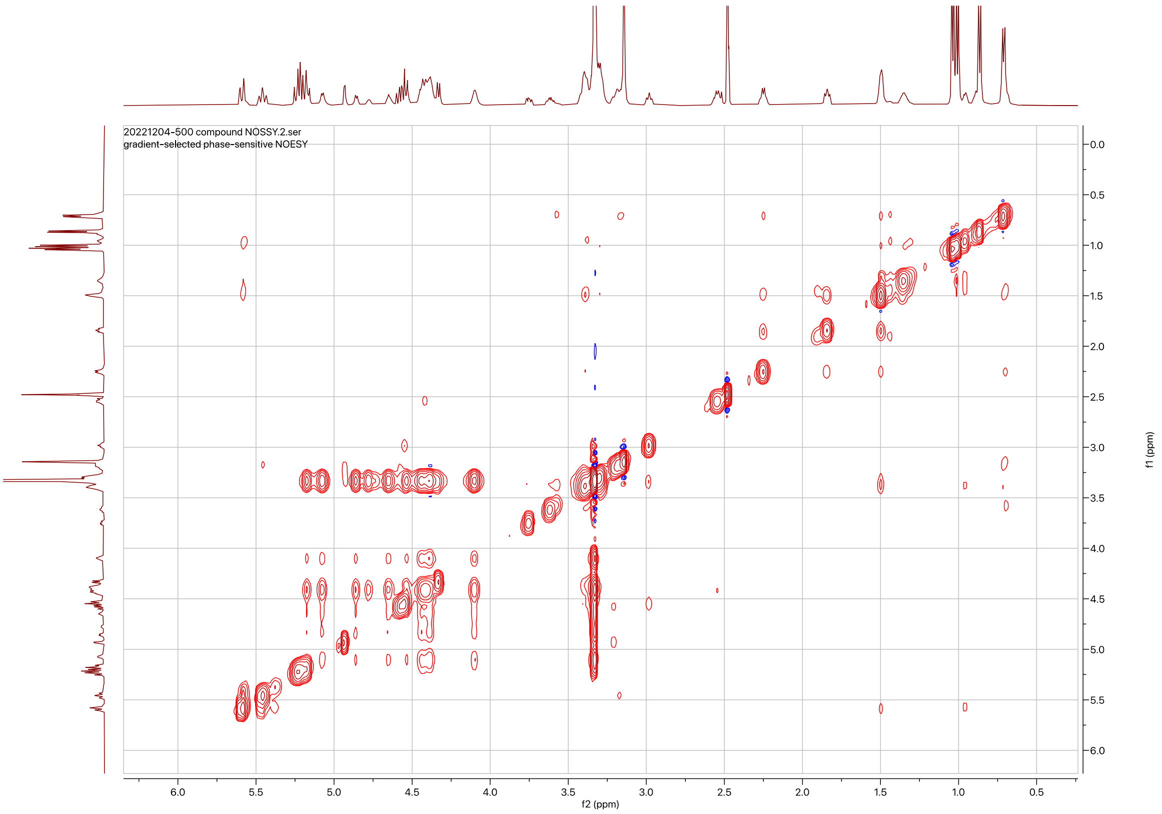

Figure S24. ^{1}H-^{1}H NOESY spectrum of compound **2** in DMSO-d_6 (500 MHz)

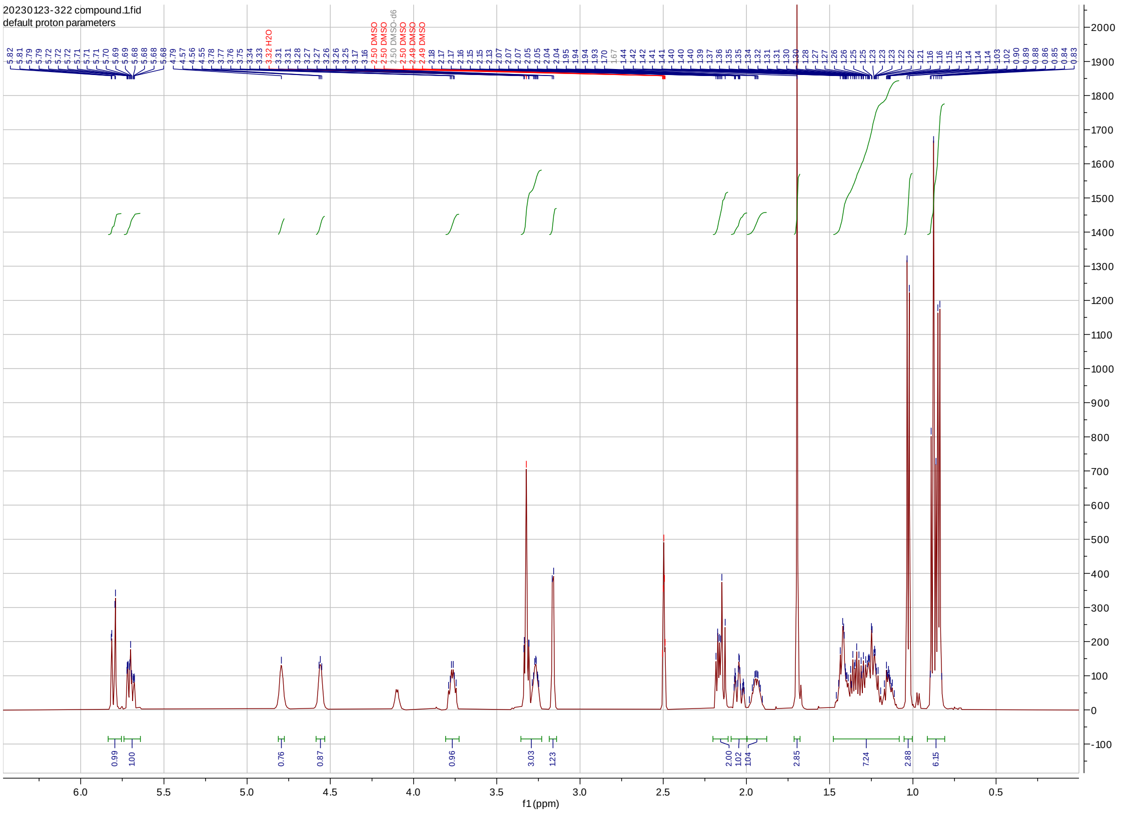

Figure S25. ^{1}H NMR spectrum of compound **3** in DMSO-d_6 (500 MHz)

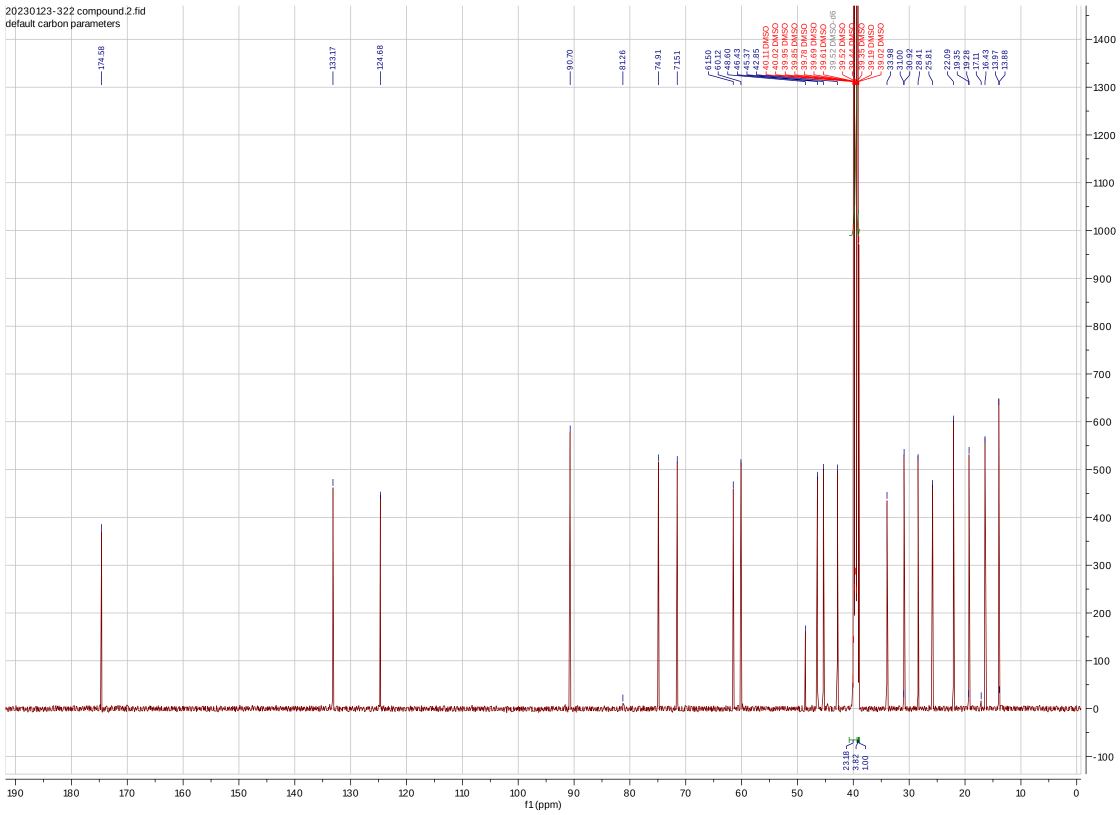

Figure S26. ^{13}C NMR spectrum of compound **3** in DMSO-d_6 (125 MHz)

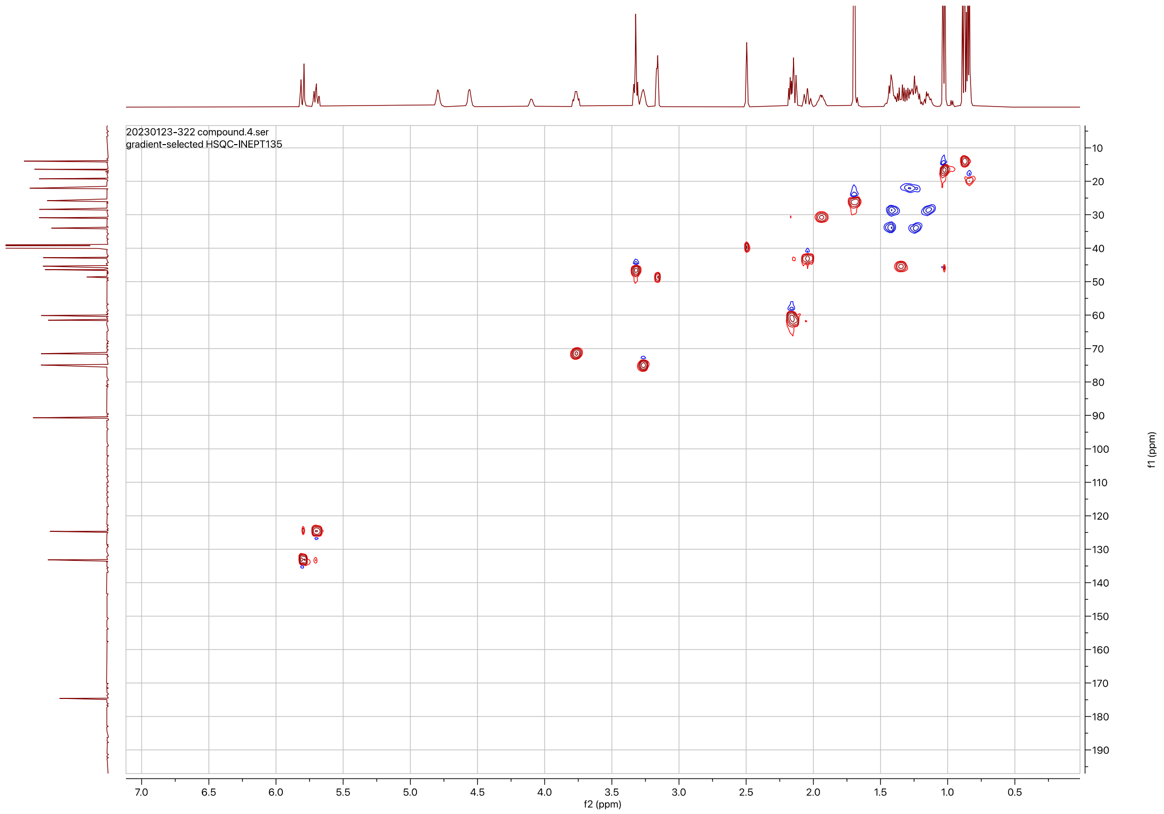

Figure S27. ^{1}H-^{13}C HSQC spectrum of compound **3** in DMSO-d_6 (500 MHz)

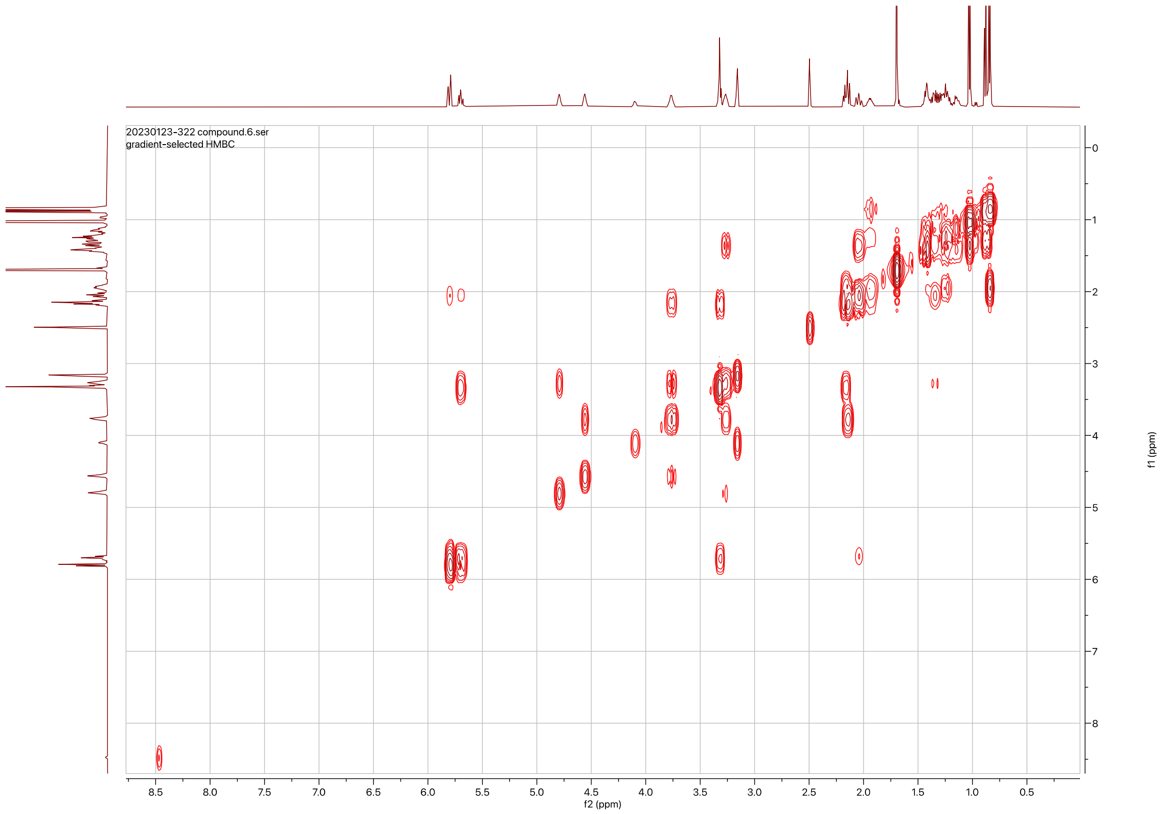

Figure S28. ^{1}H-^{1}H COSY spectrum of compound **3** in DMSO-d_6 (500 MHz)

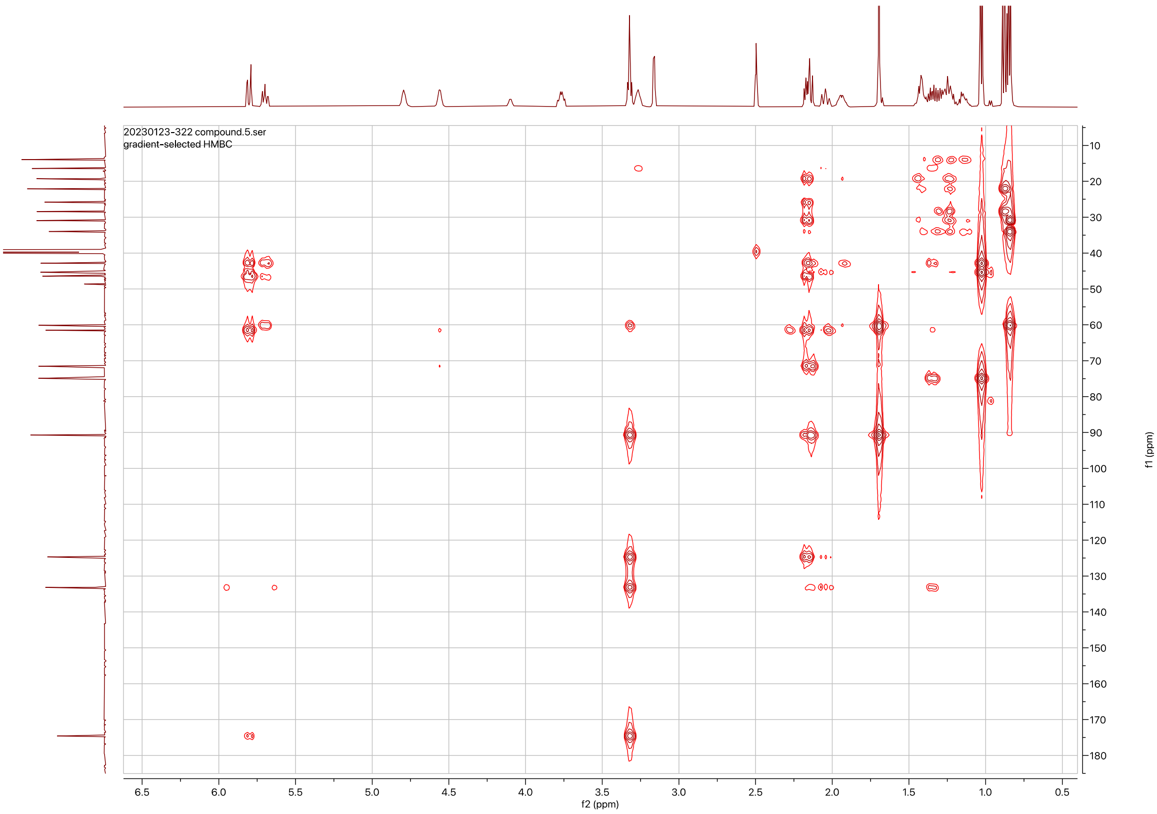

Figure S29. ^{1}H-^{13}C HMBC spectrum of compound **3** in DMSO-*d*6 (500 MHz)

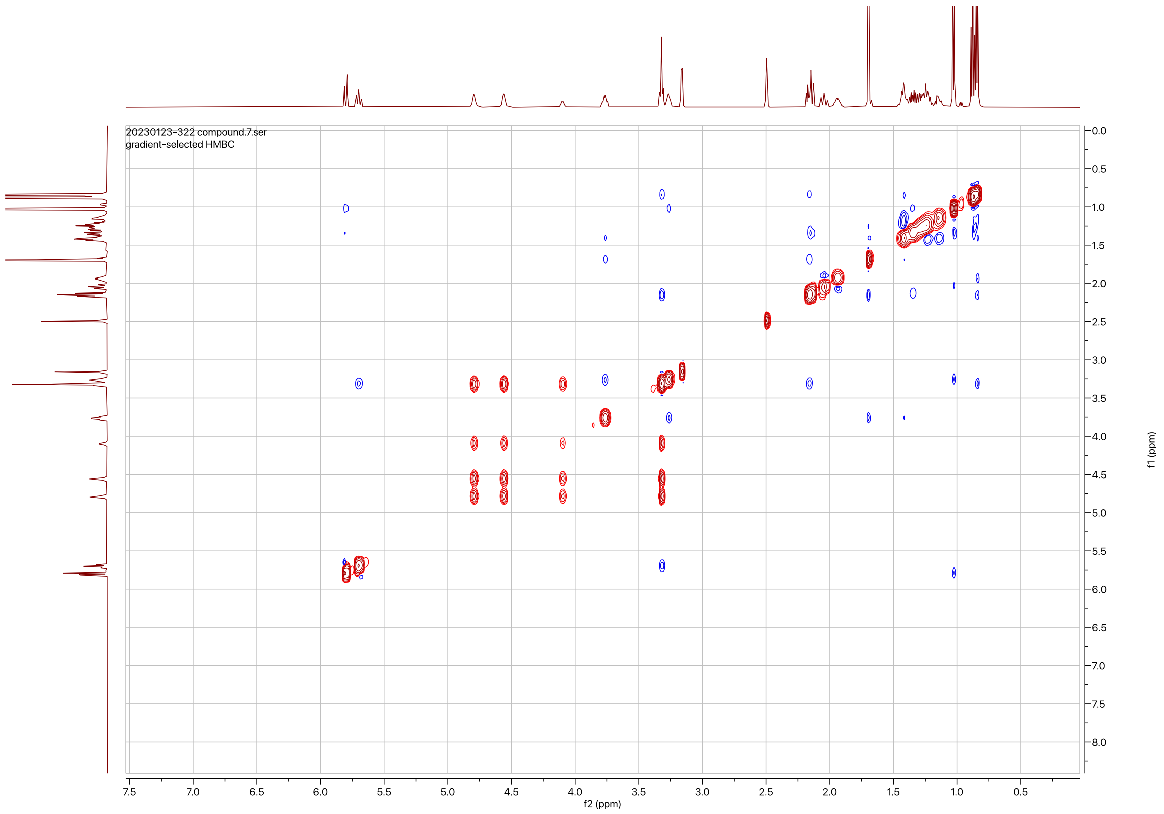

Figure S30. ^{1}H-^{1}H NOESY spectrum of compound **3** in DMSO-*d*$_6$ (500 MHz)

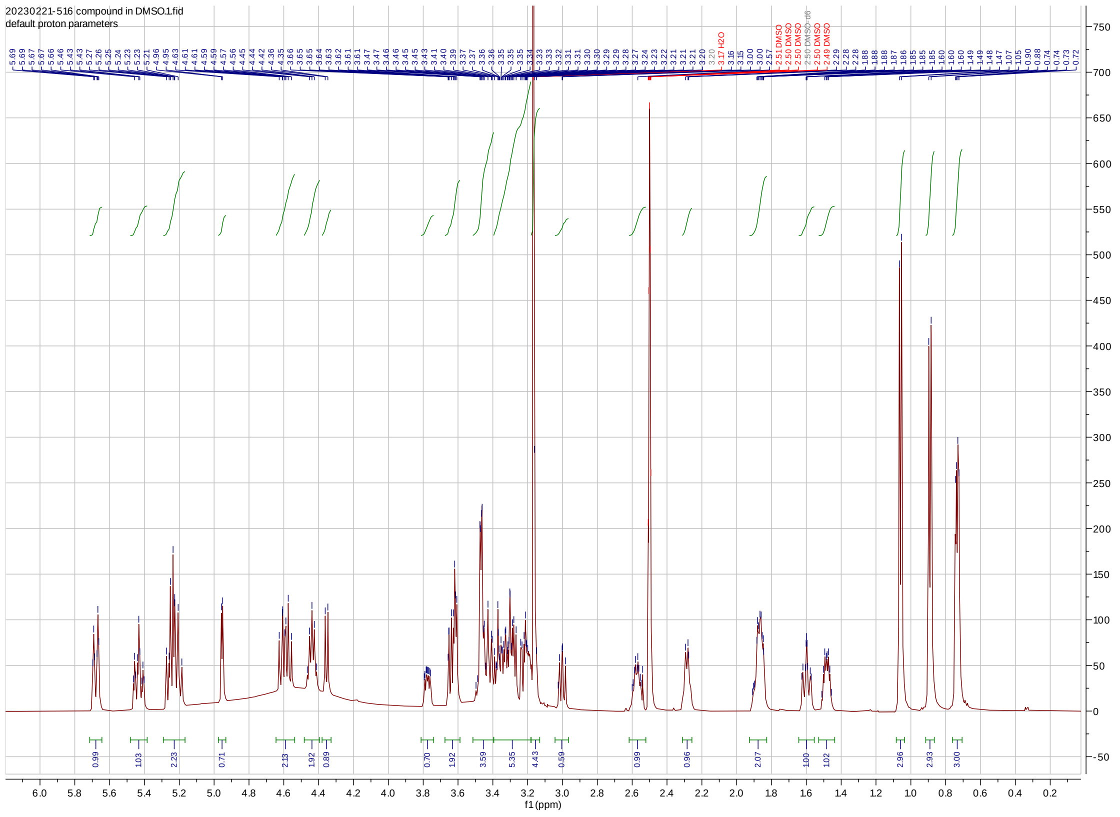

Figure S31. ^{1}H NMR spectrum of compound **4** in DMSO-d_6 (500 MHz)

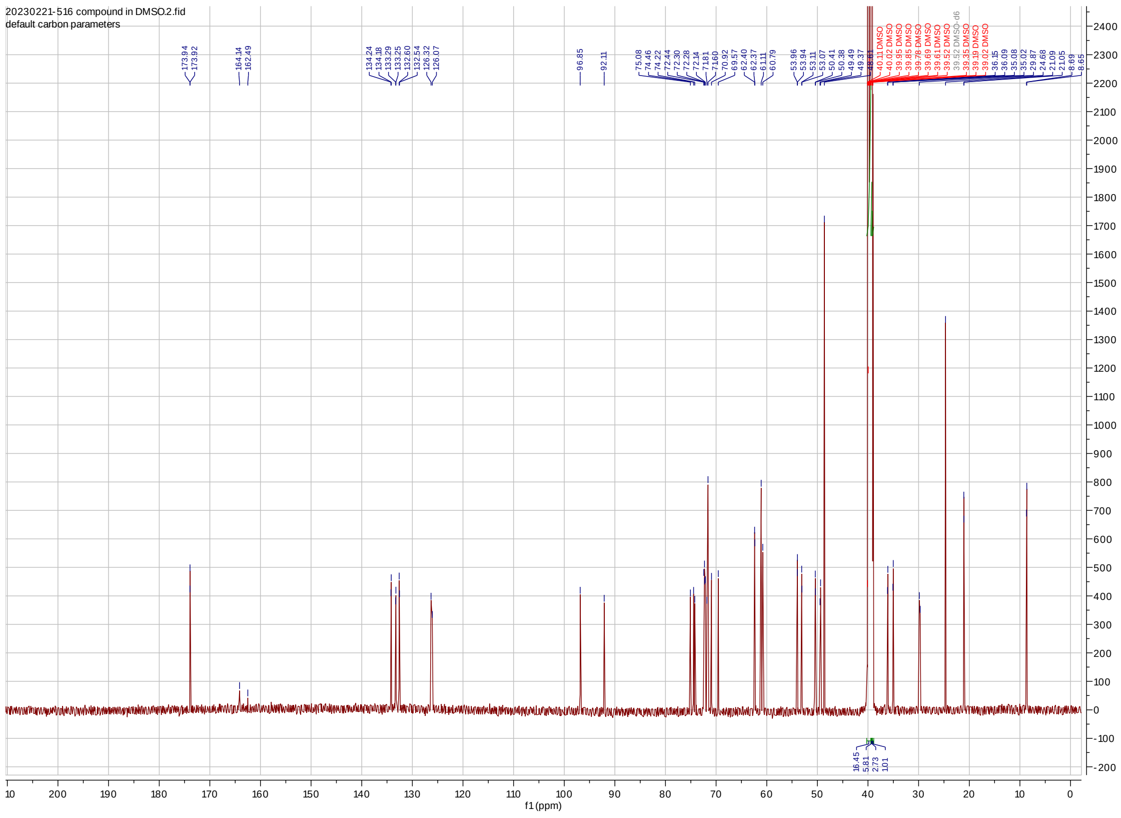

Figure S32. ^{13}C NMR spectrum of compound **4** in DMSO-d_6 (125 MHz)

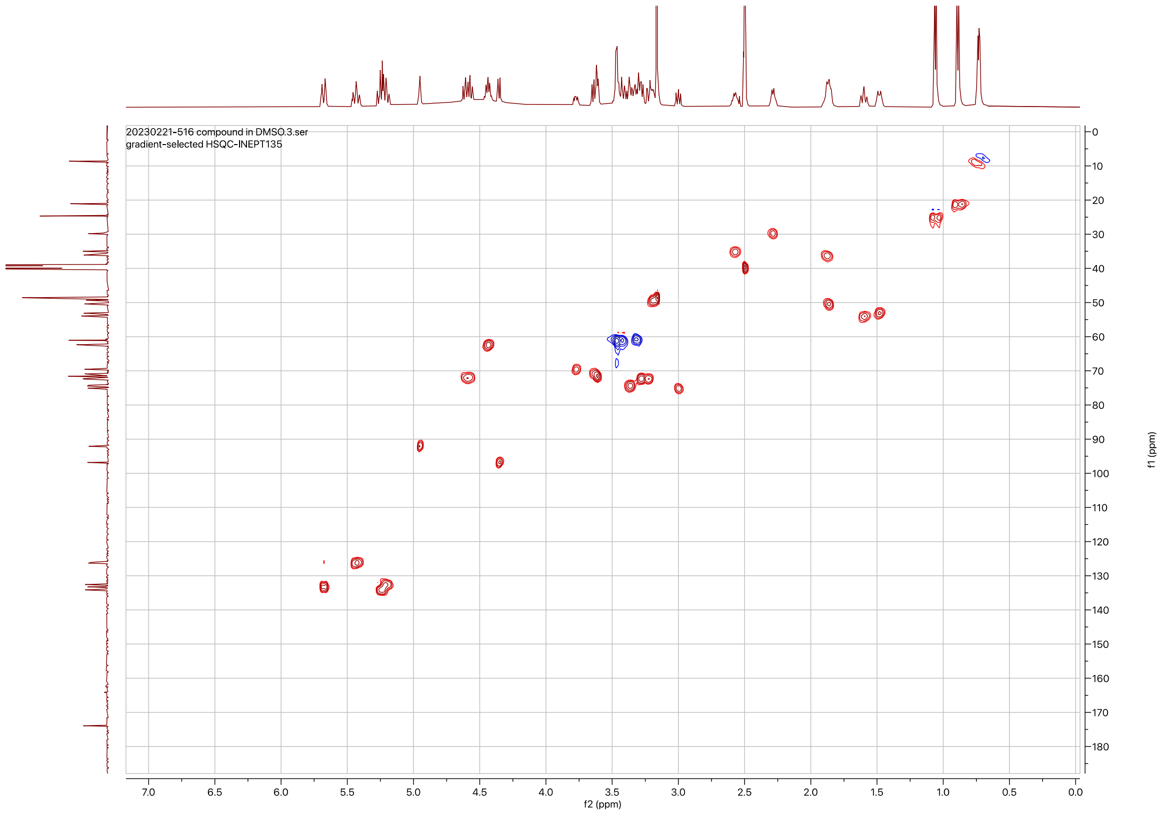

Figure S33. ^{1}H-^{13}C HSQC spectrum of compound **4** in DMSO-*d*$_6$ (500 MHz)

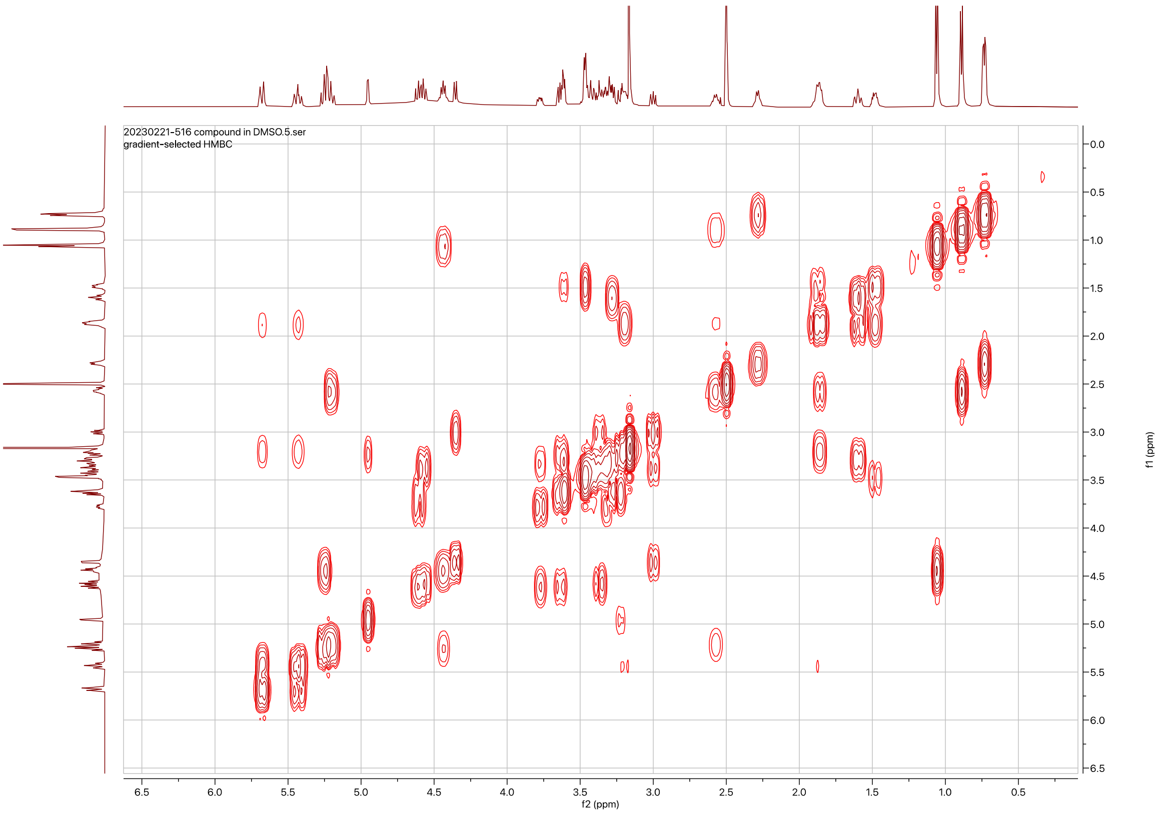

Figure S34. ^{1}H-^{1}H COSY spectrum of compound **4** in DMSO-d_6 (500 MHz)

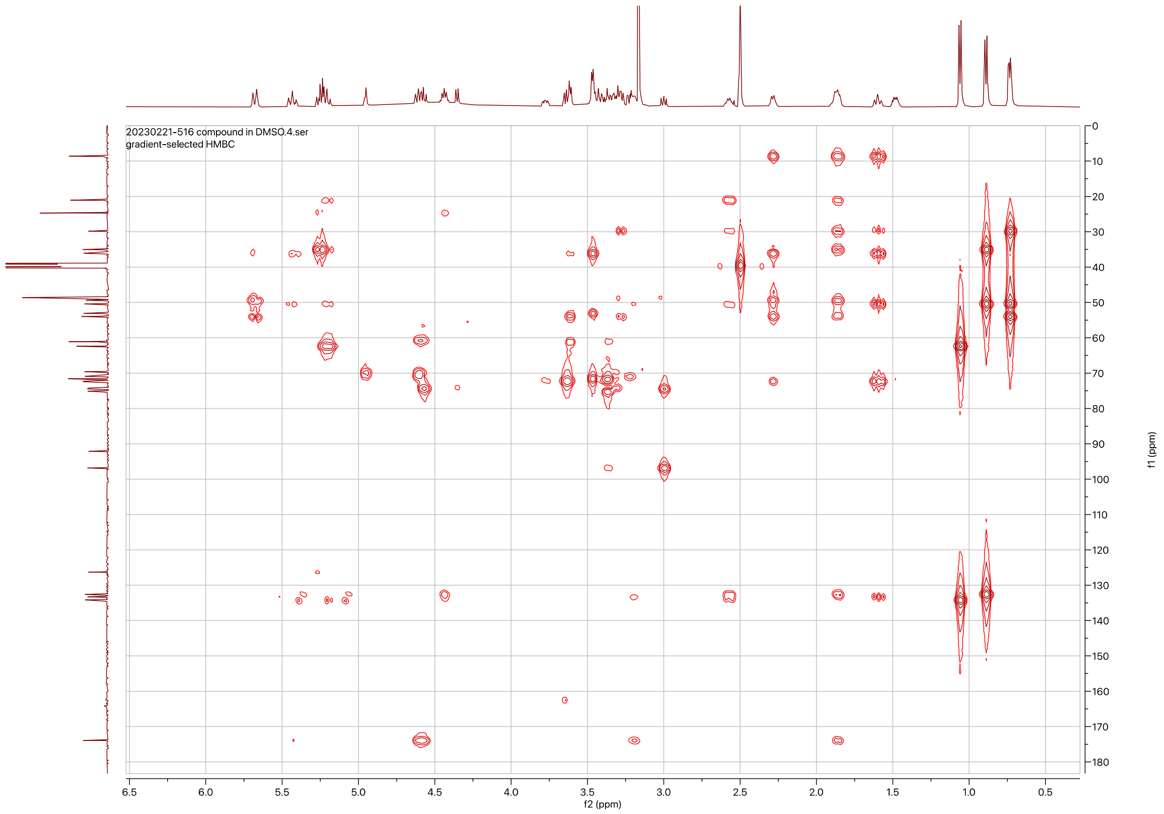

Figure S35. ^{1}H-^{13}C HMBC spectrum of compound **4** in DMSO-d_6 (500 MHz)

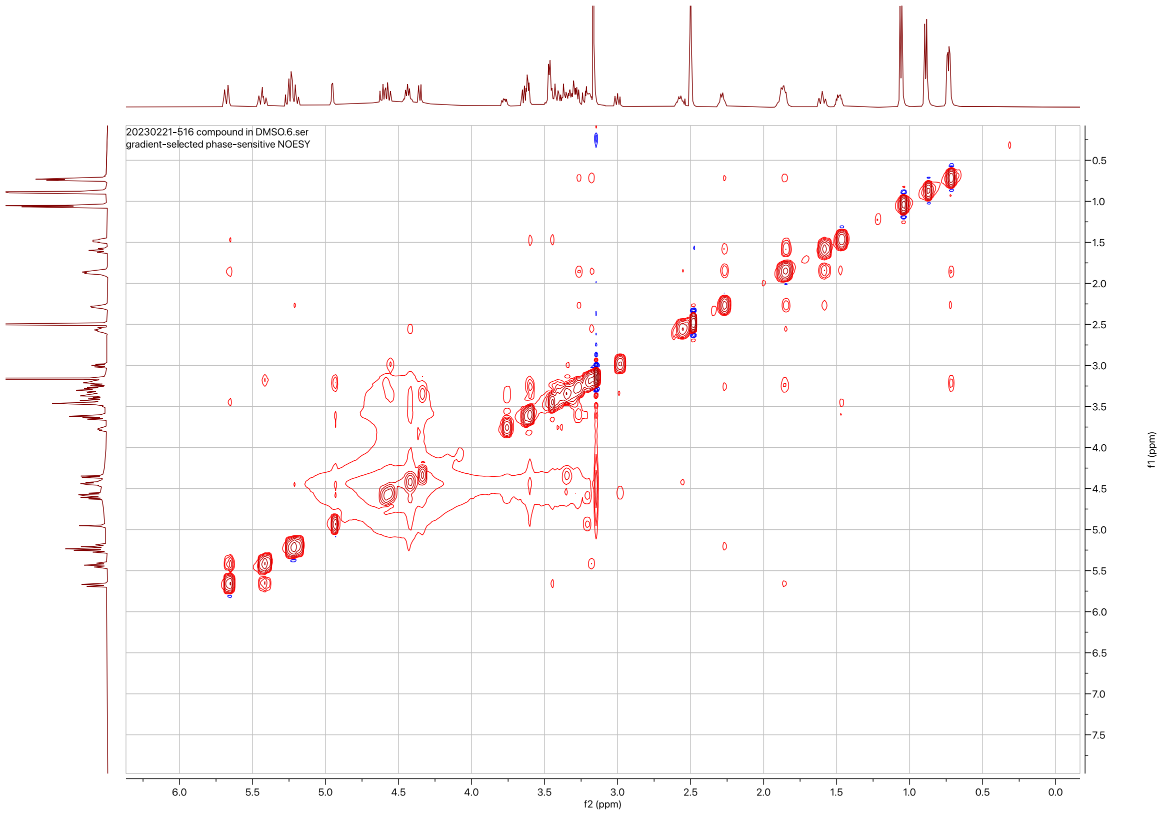

Figure S36. ^{1}H-^{1}H NOESY spectrum of compound **4** in DMSO-d_6 (500 MHz)

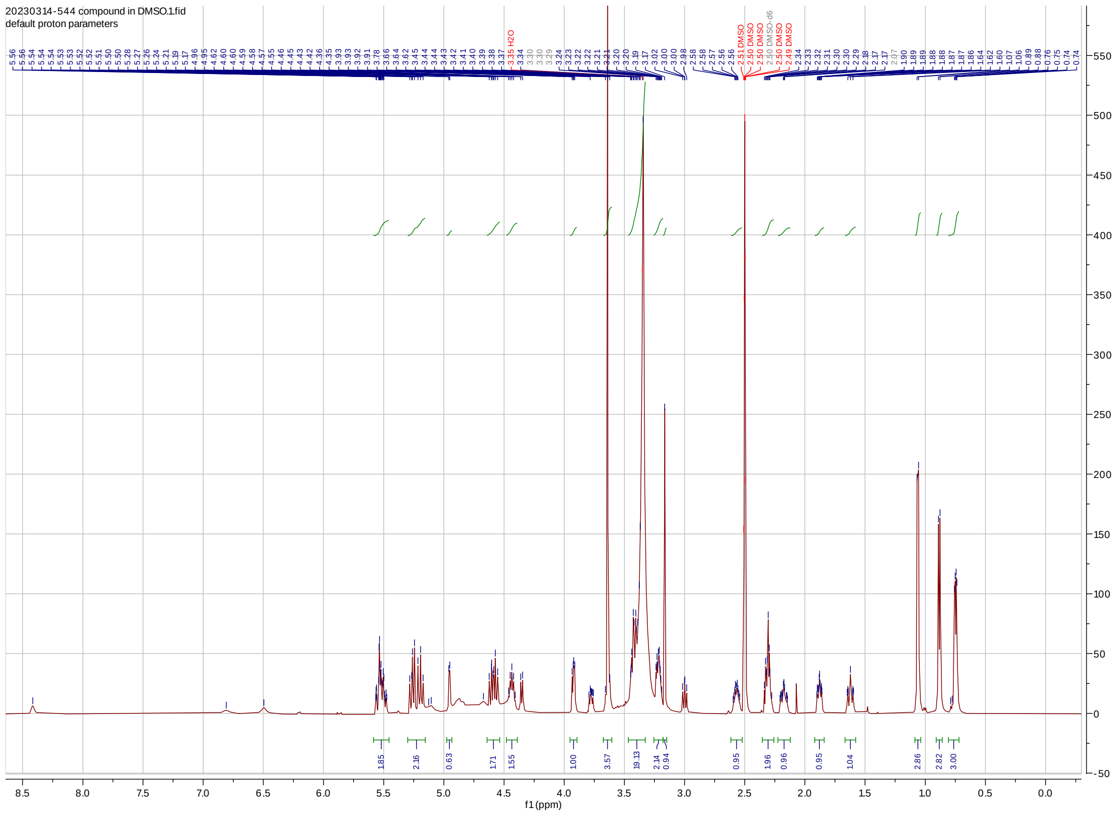

Figure S37. ^{1}H NMR spectrum of compound **5** in DMSO-d_6 (500 MHz)

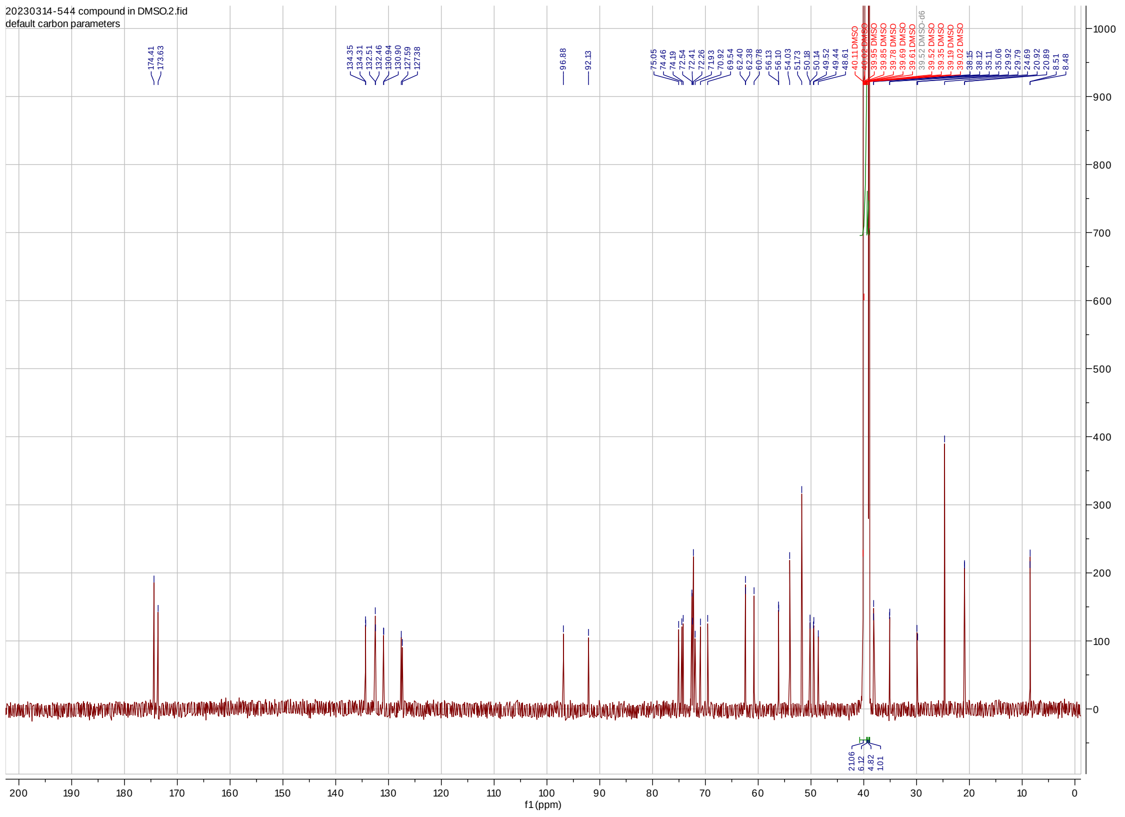

Figure S38. ^{13}C NMR spectrum of compound **5** in DMSO-d_6 (125 MHz)

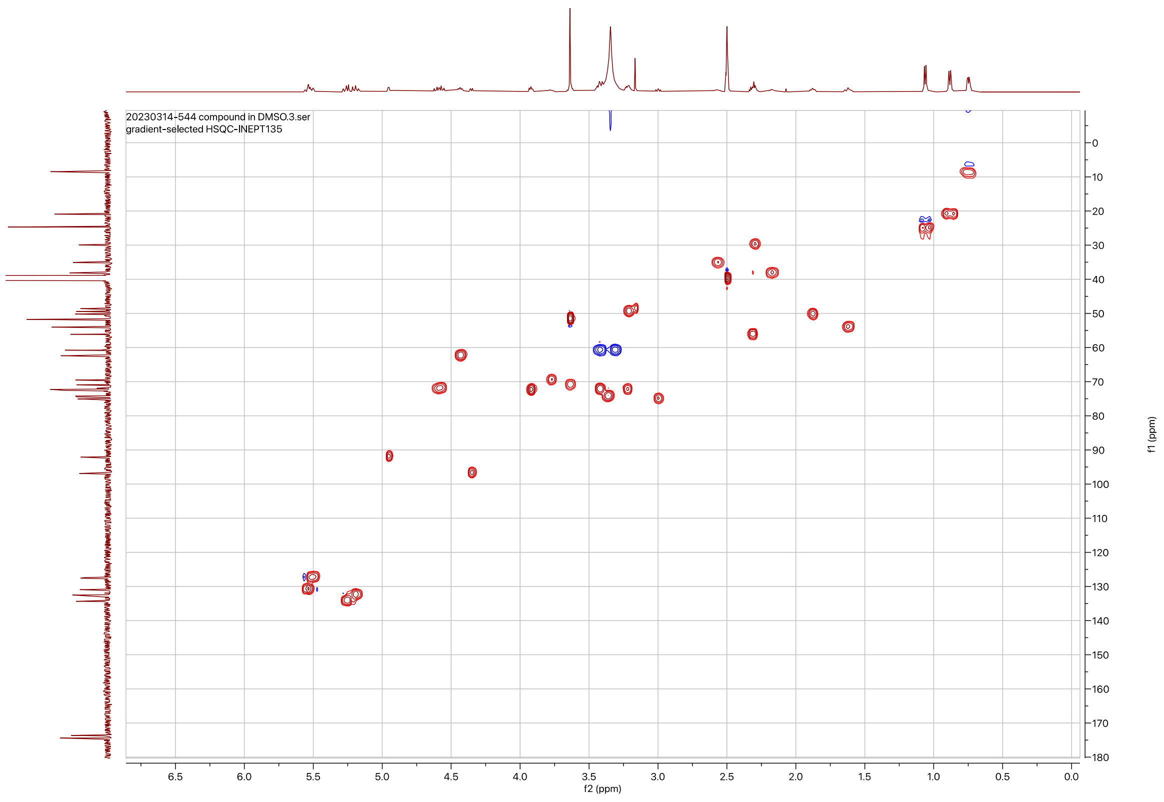

Figure S39. ^{1}H-^{13}C HSQC spectrum of compound **5** in DMSO-d_6 (500 MHz)

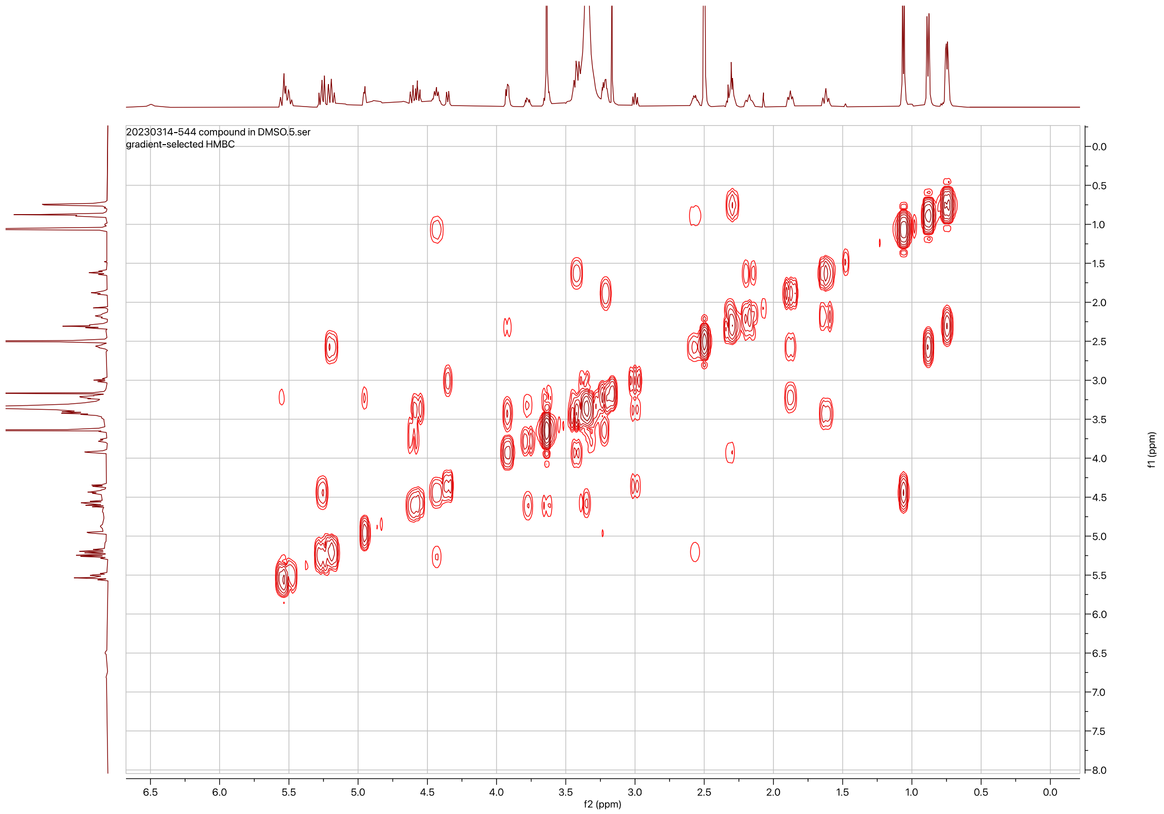

Figure S40. ^{1}H-^{1}H COSY spectrum of compound **5** in DMSO-d_6 (500 MHz)

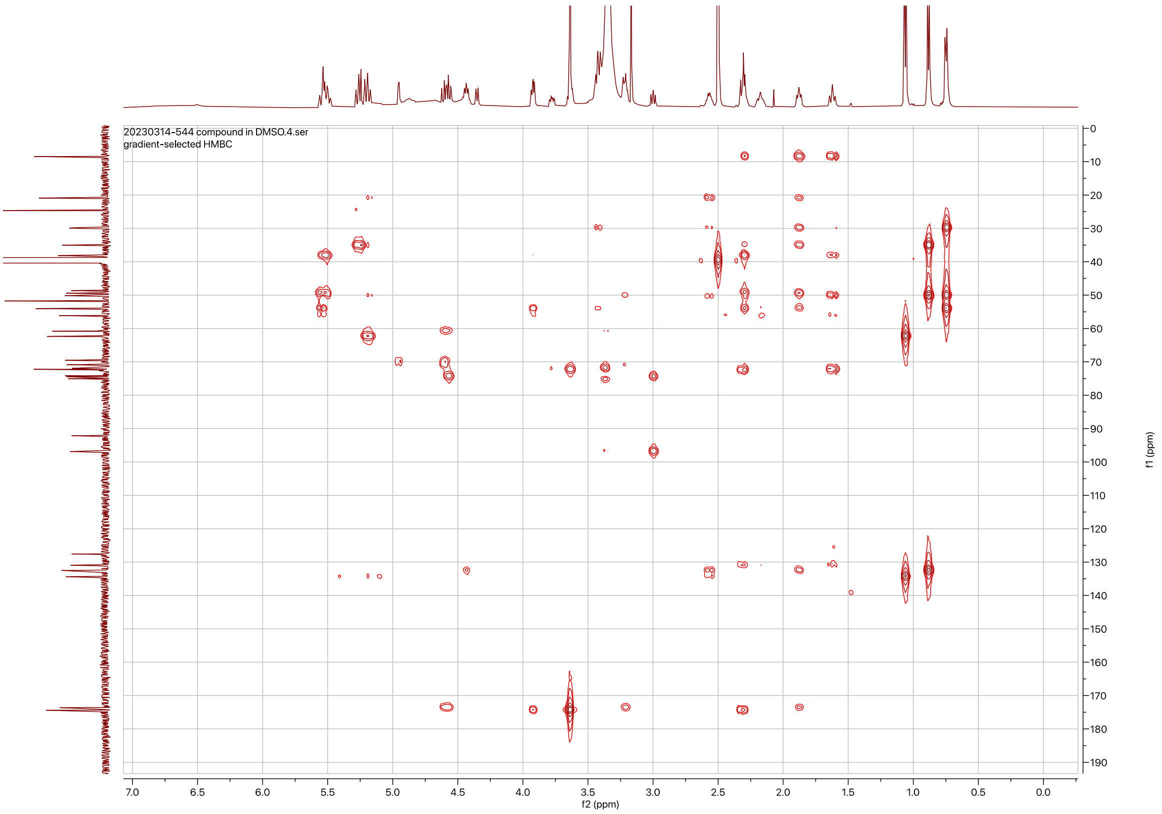

Figure S41. ^{1}H-^{13}C HMBC spectrum of compound **5** in DMSO-d_6 (500 MHz)

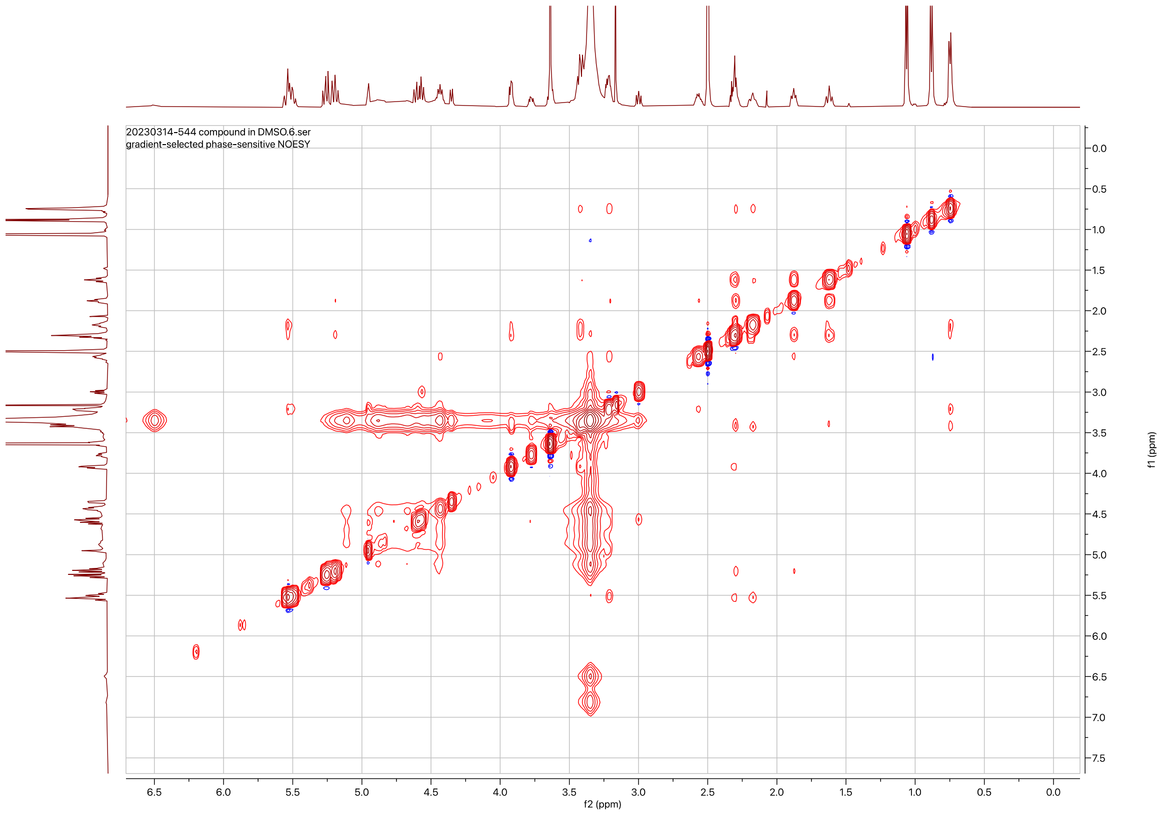

Figure S42. ^{1}H-^{1}H NOESY spectrum of compound **5** in DMSO-d_6 (500 MHz)

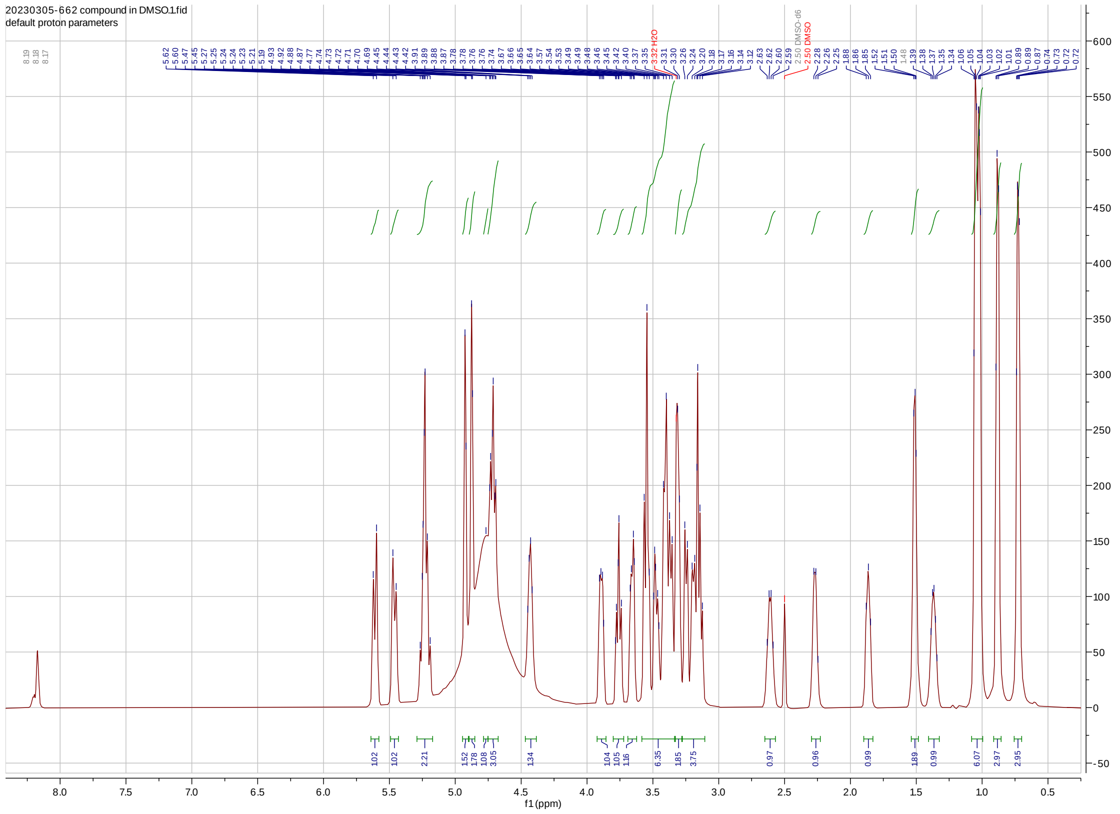

Figure S43. ^{1}H NMR spectrum of compound **7** in DMSO-d_6 (500 MHz)

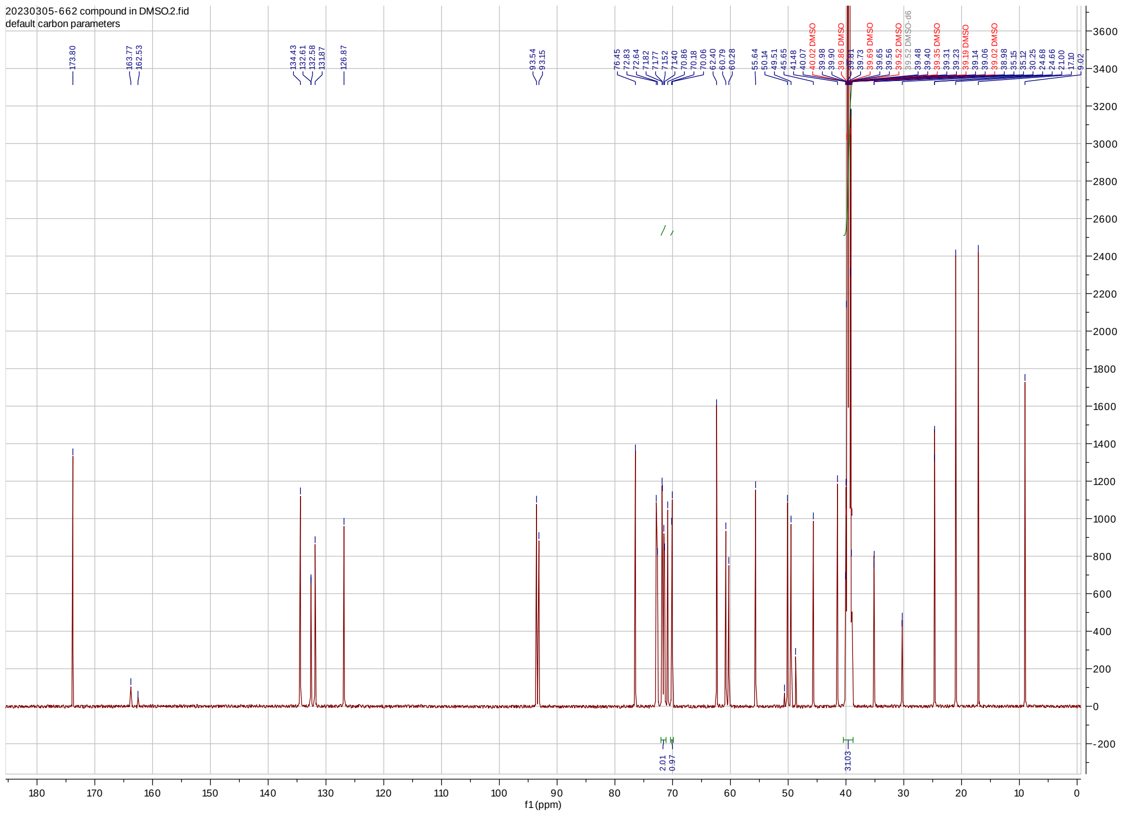

Figure S44. ^{13}C NMR spectrum of compound **7** in DMSO-d_6 (125 MHz)

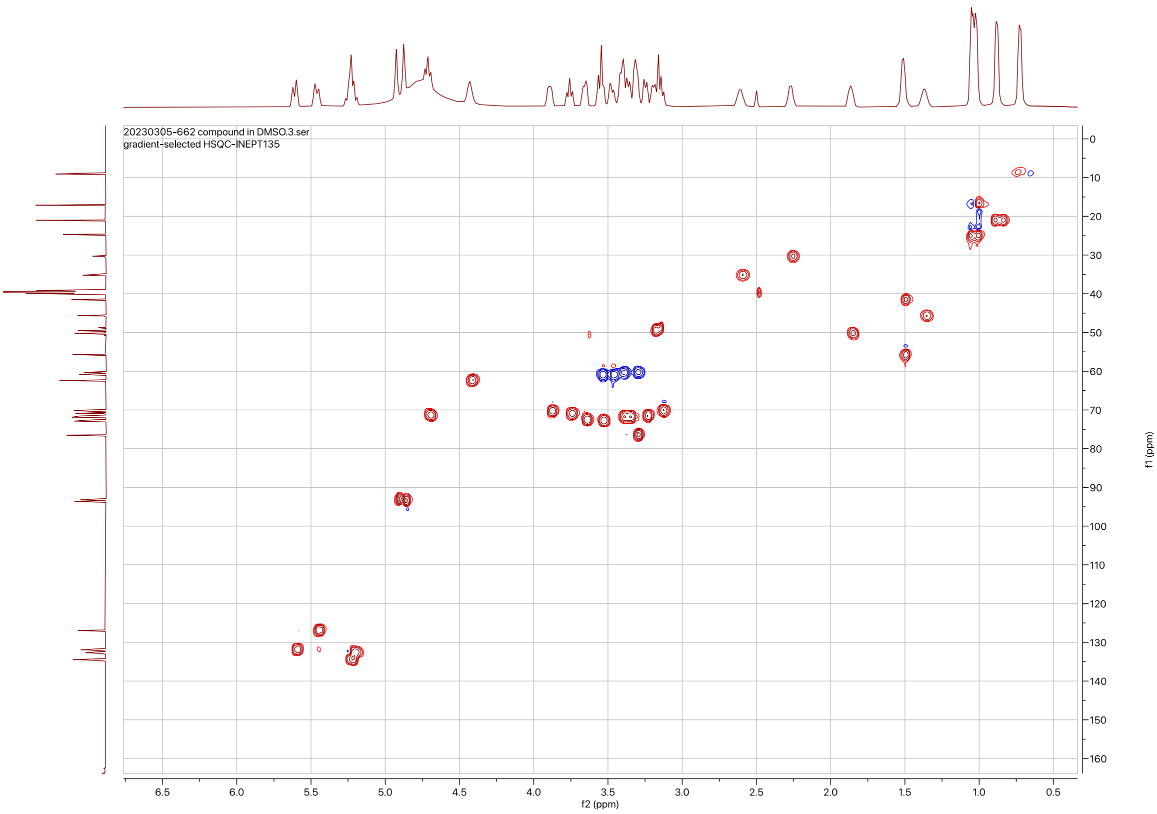

Figure S45. ^{1}H-^{13}C HSQC spectrum of compound **7** in DMSO-*d₆* (500 MHz)

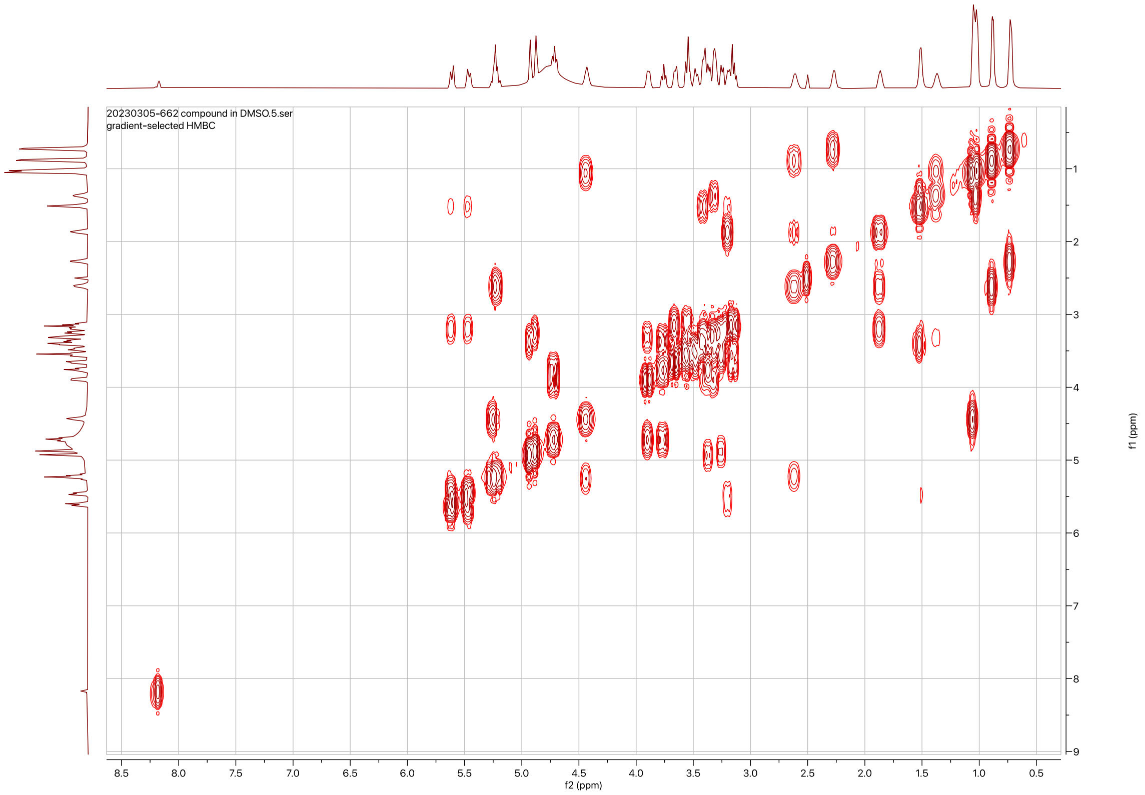

Figure S46. ^{1}H-^{1}H COSY spectrum of compound **7** in DMSO-d_6 (500 MHz)

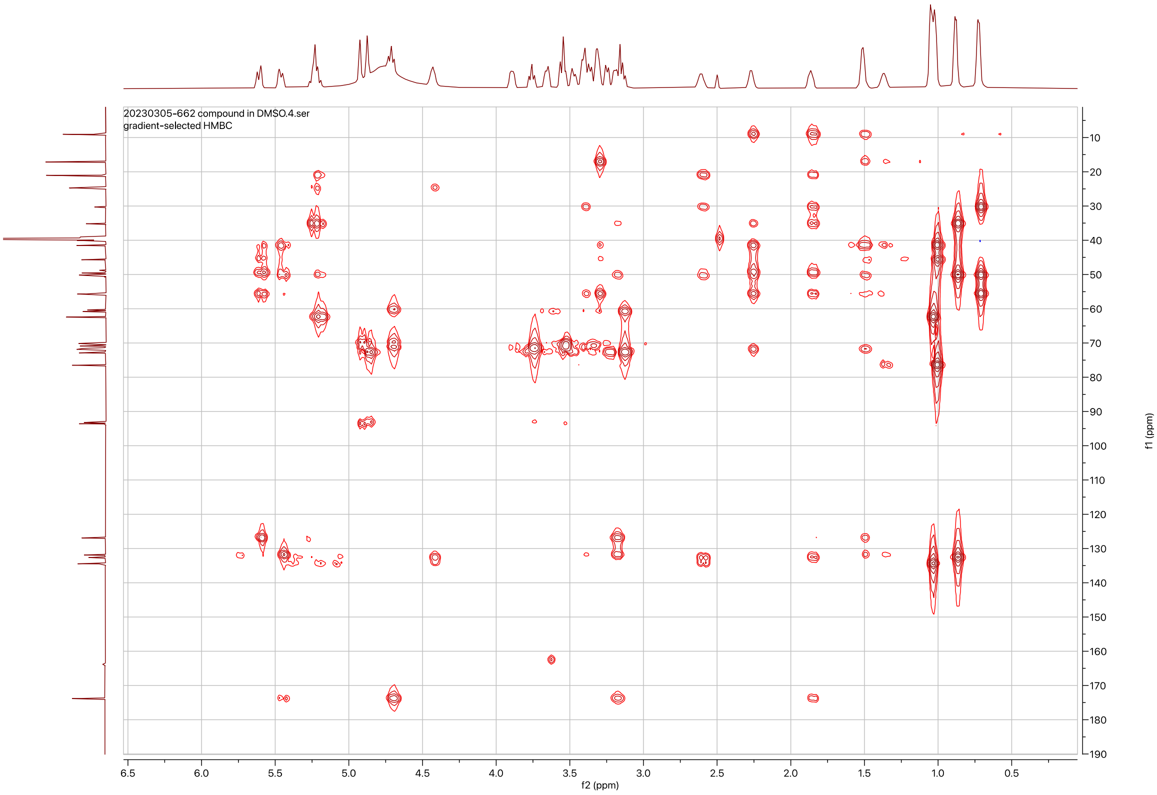

Figure S47. ^{1}H-^{13}C HMBC spectrum of compound **7** in DMSO-*d*$_6$ (500 MHz)

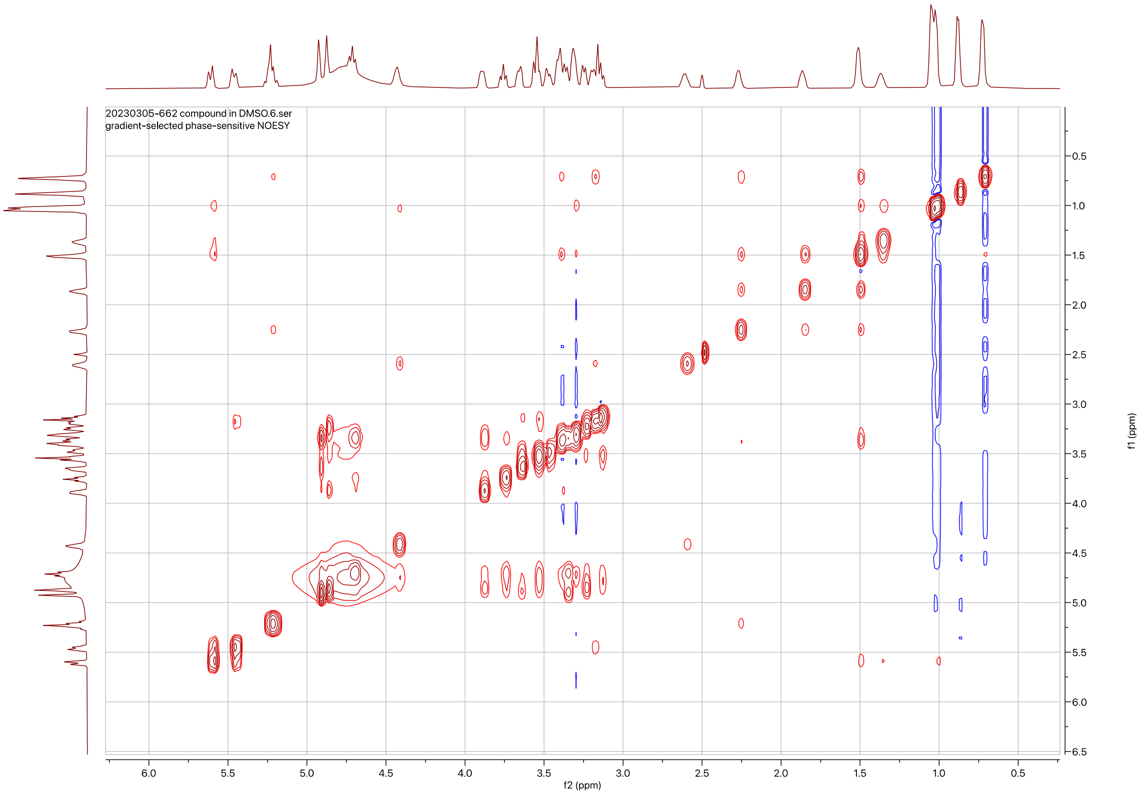

Figure S48. ^{1}H-^{1}H NOESY spectrum of compound **7** in DMSO-*d*6 (500 MHz)

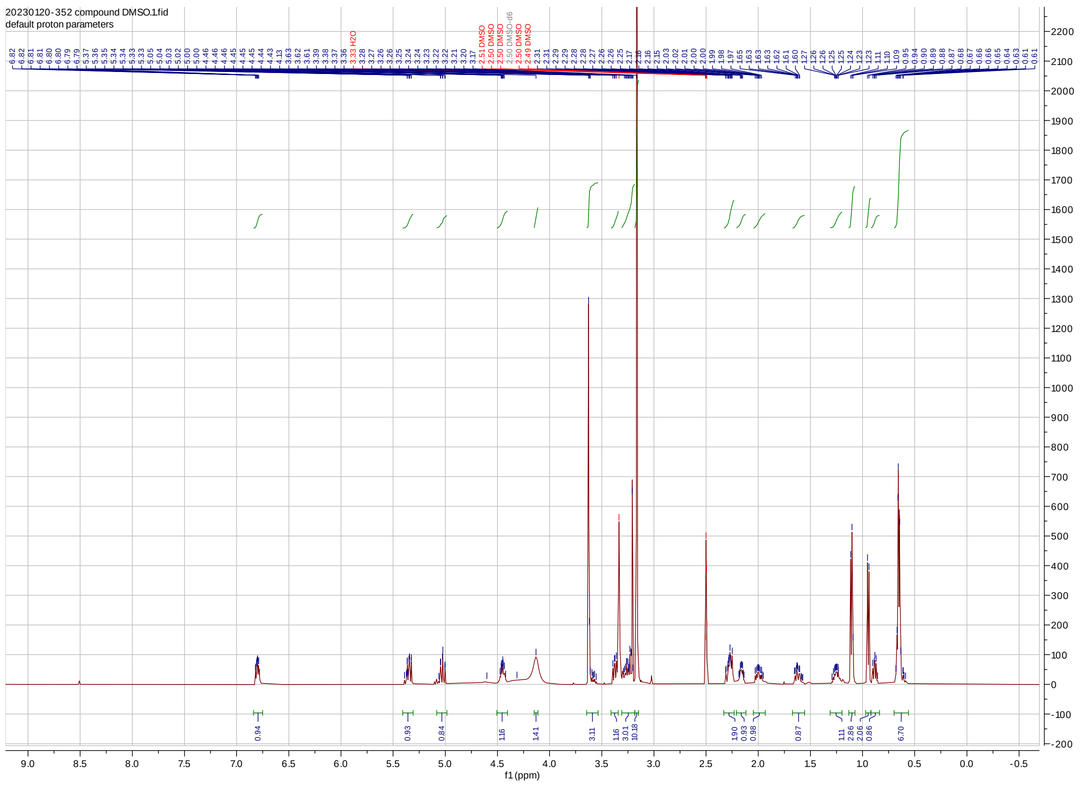

Figure S49. ^{1}H NMR spectrum of compound **7a** in DMSO-d_6 (500 MHz)

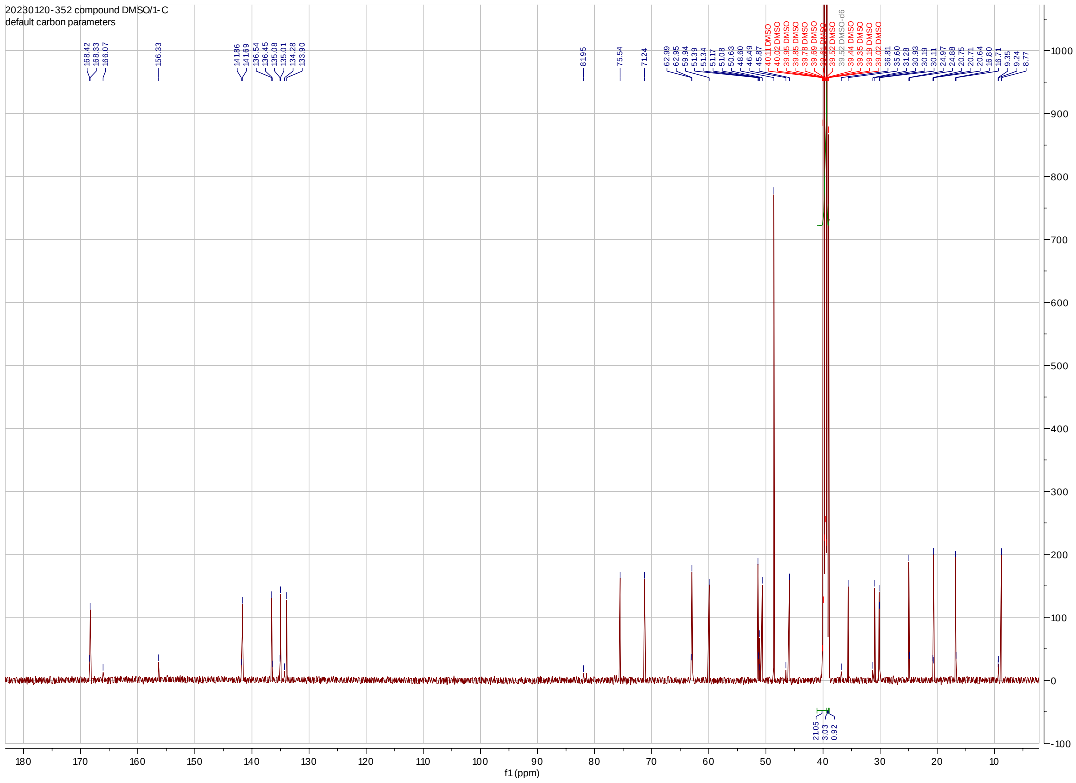

Figure S50. ^{13}C NMR spectrum of compound **7a** in DMSO-d_6 (125 MHz)

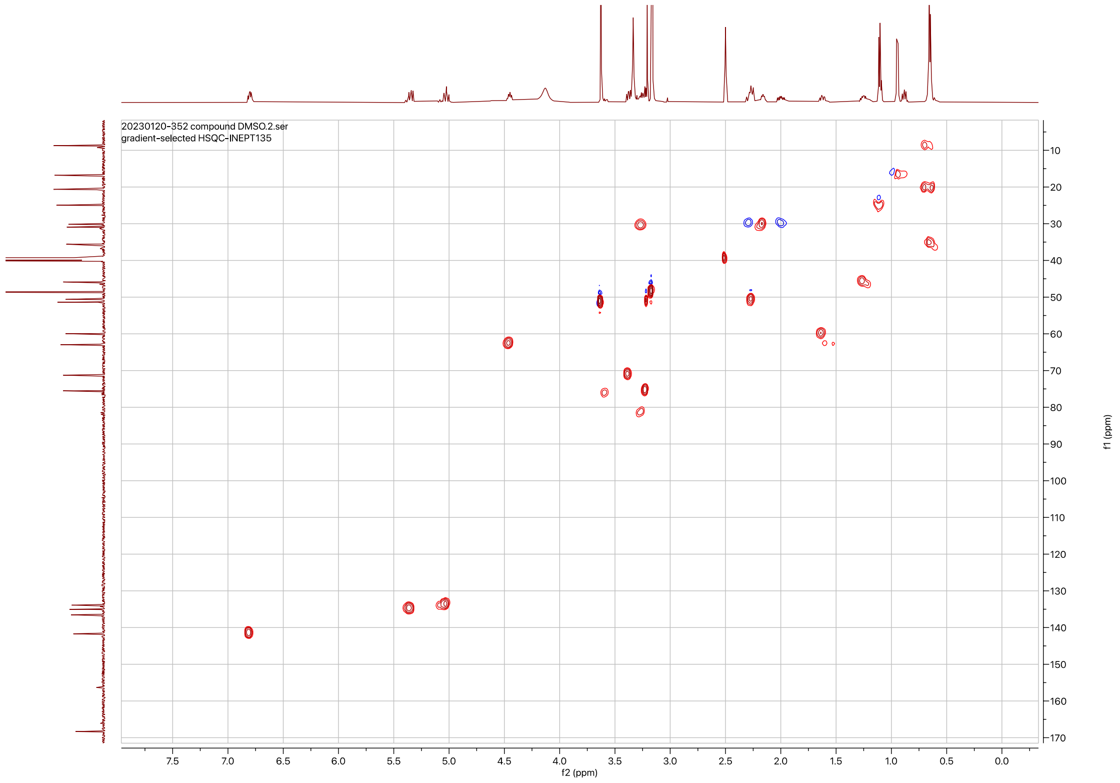

Figure S51. ^{1}H-^{13}C HSQC spectrum of compound **7a** in DMSO-d_6 (500 MHz)

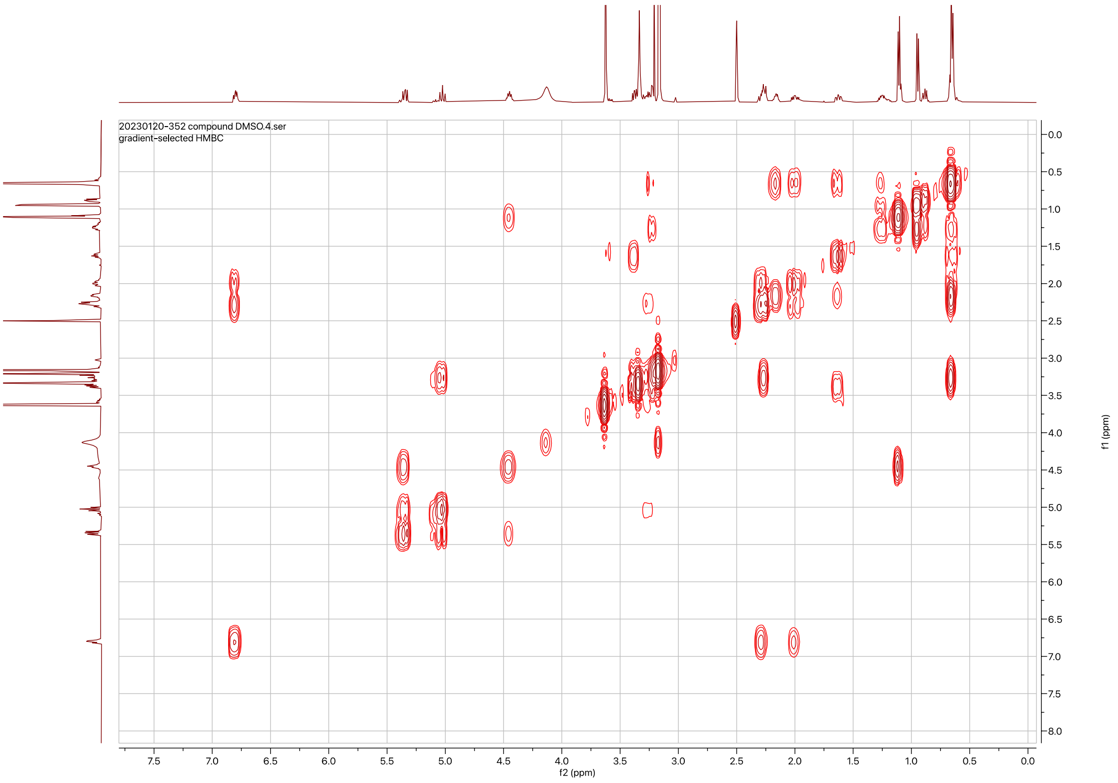

Figure S52. ^1H-^1H COSY spectrum of compound **7a** in DMSO-d_6 (500 MHz)

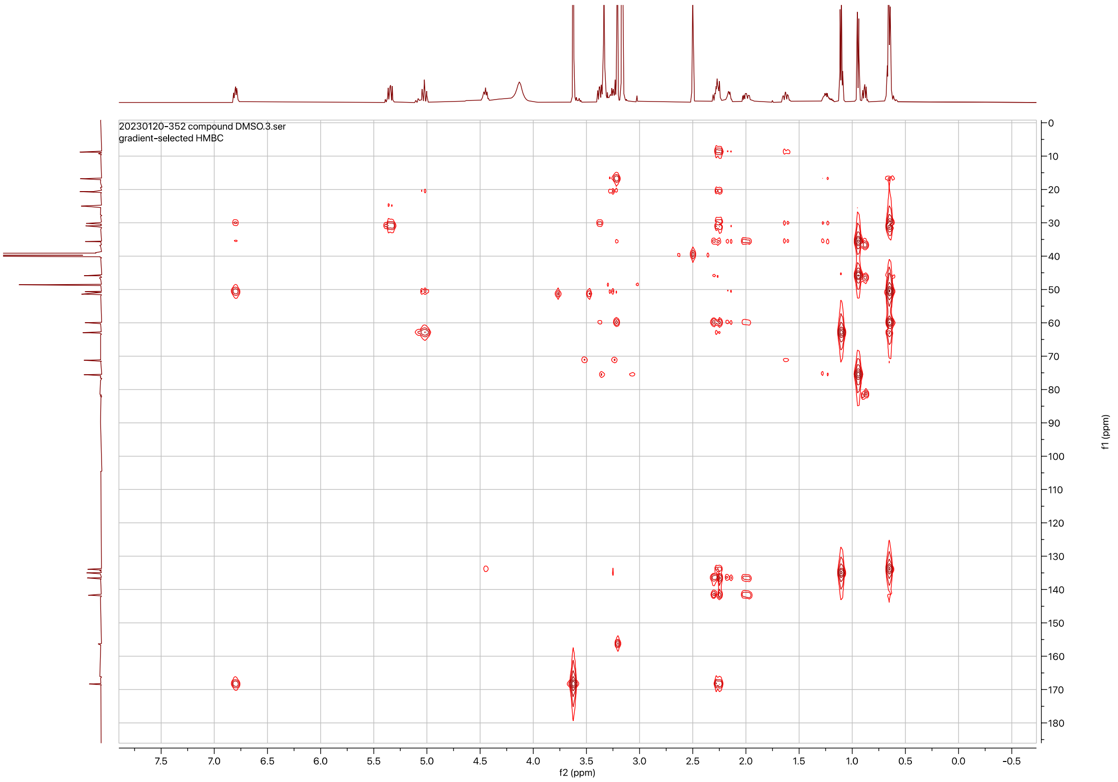

Figure S53. ^{1}H-^{13}C HMBC spectrum of compound **7a** in DMSO-d_6 (500 MHz)

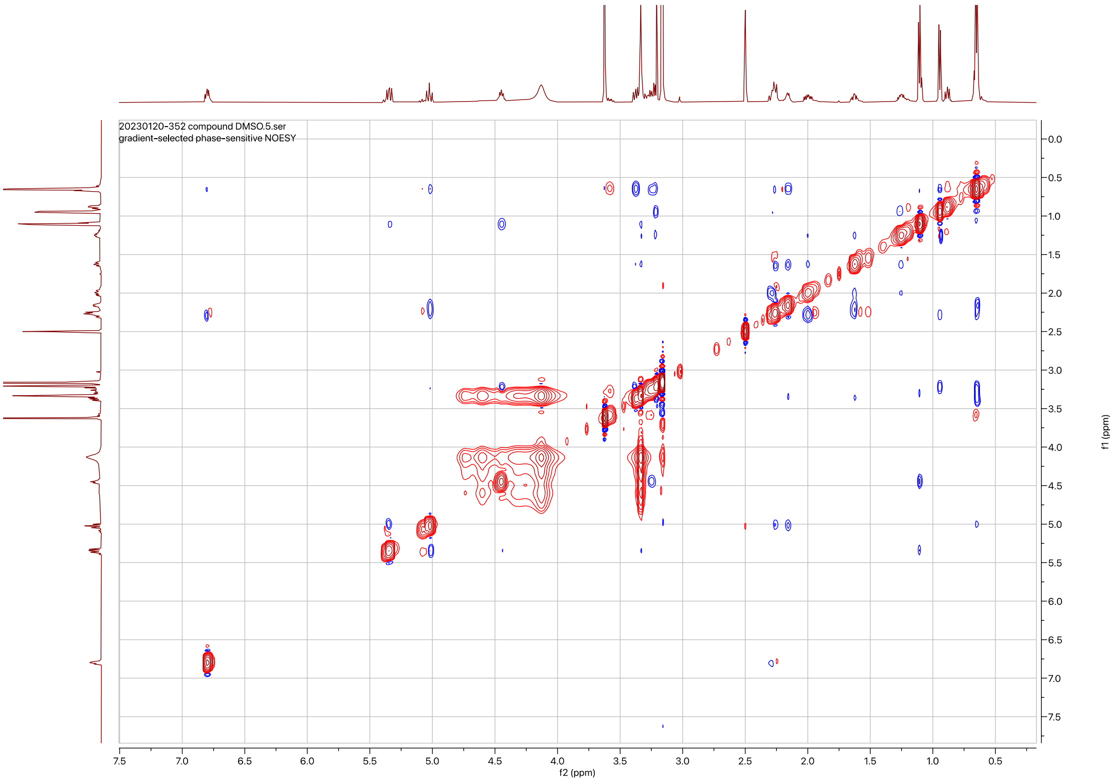

Figure S54. ^{1}H-^{1}H NOESY spectrum of compound **7a** in DMSO-*d₆* (500 MHz)

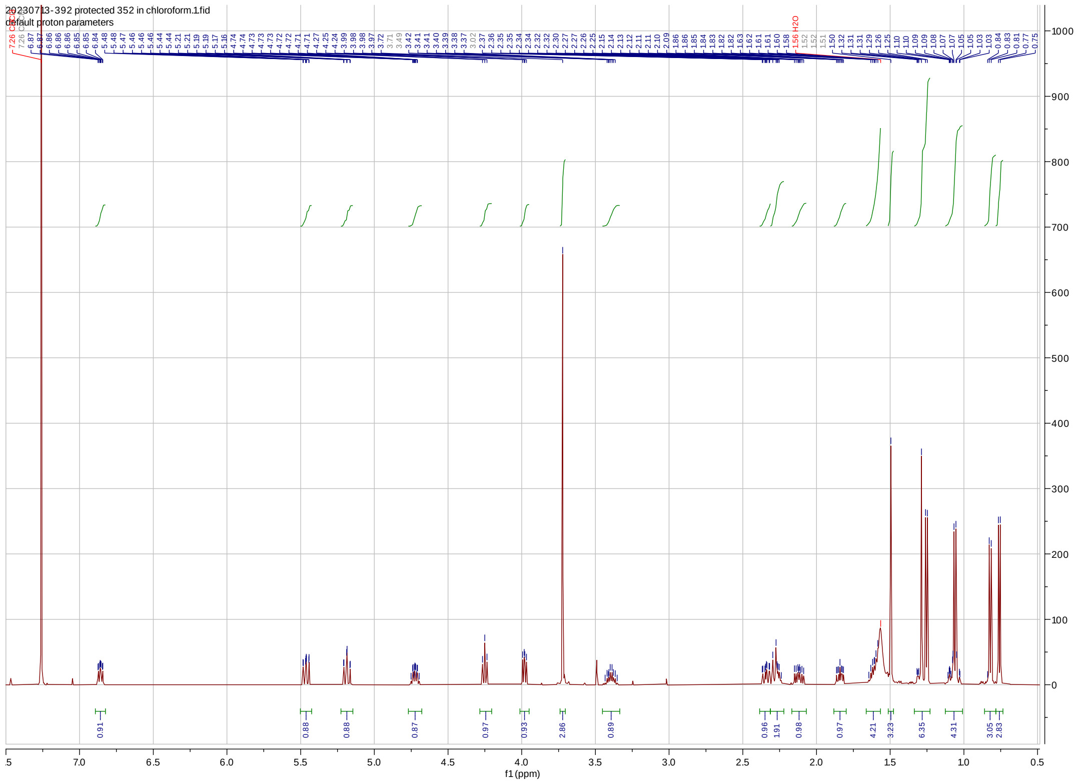

Figure S55. ^{1}H NMR spectrum of compound **7b** in $CDCl_3$ (500 MHz)

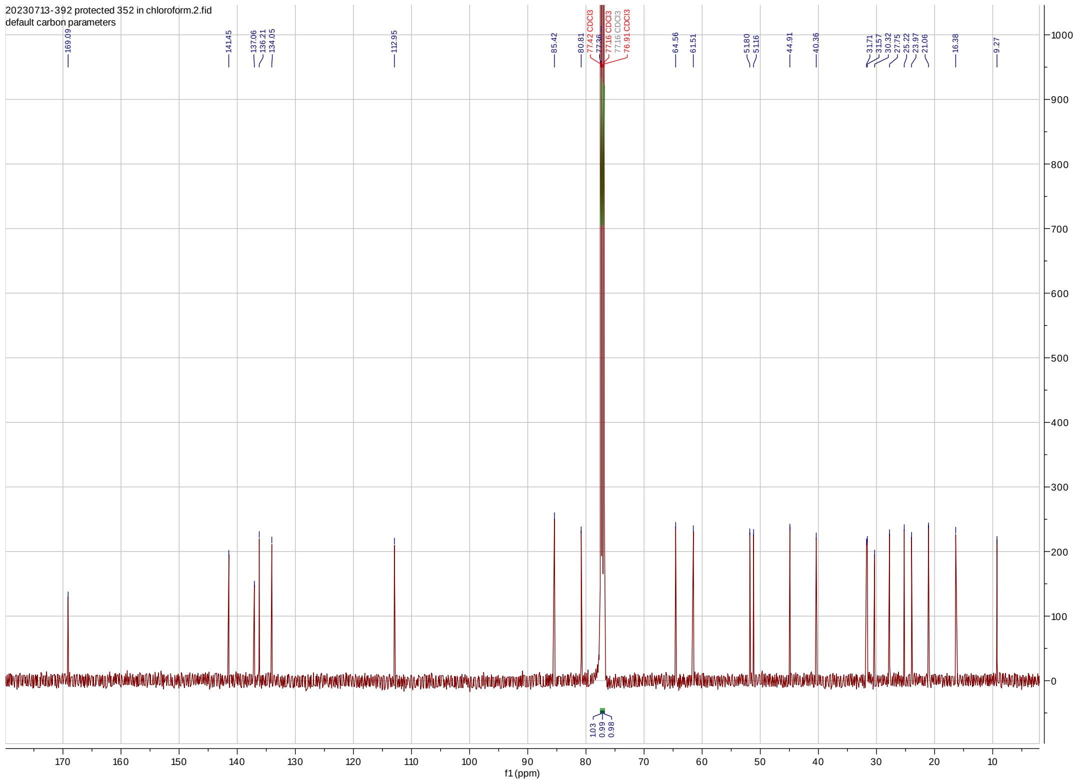

Figure S56. ^{13}C NMR spectrum of compound **7b** in $CDCl_3$ (125 MHz)

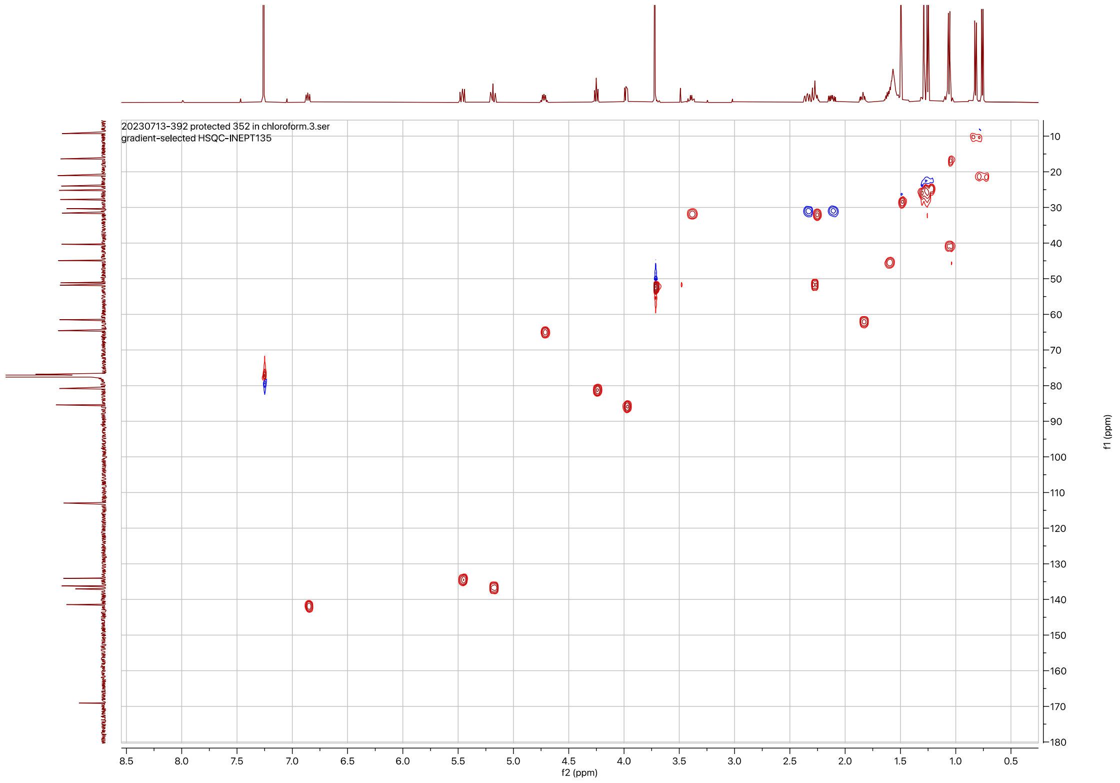

Figure S57. ^{1}H-^{13}C HSQC spectrum of compound **7b** in $CDCl_3$ (500 MHz)

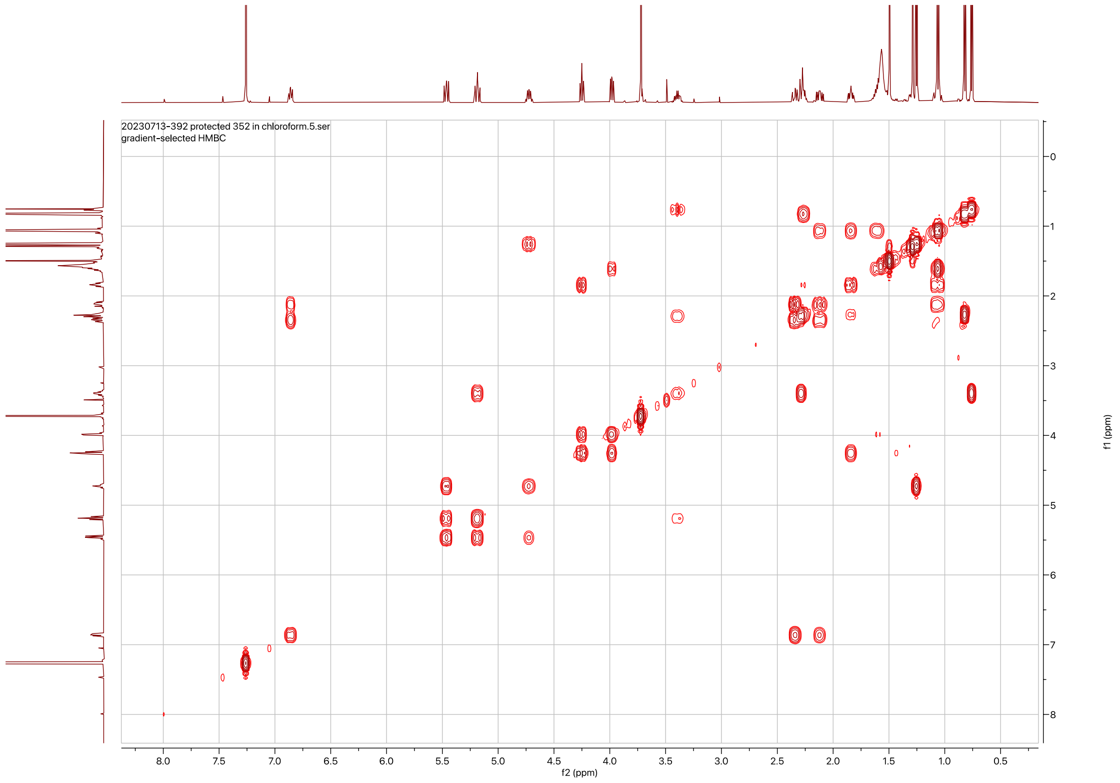

Figure S58. ^{1}H-^{1}H COSY spectrum of compound **7b** in $CDCl_3$ (500 MHz)

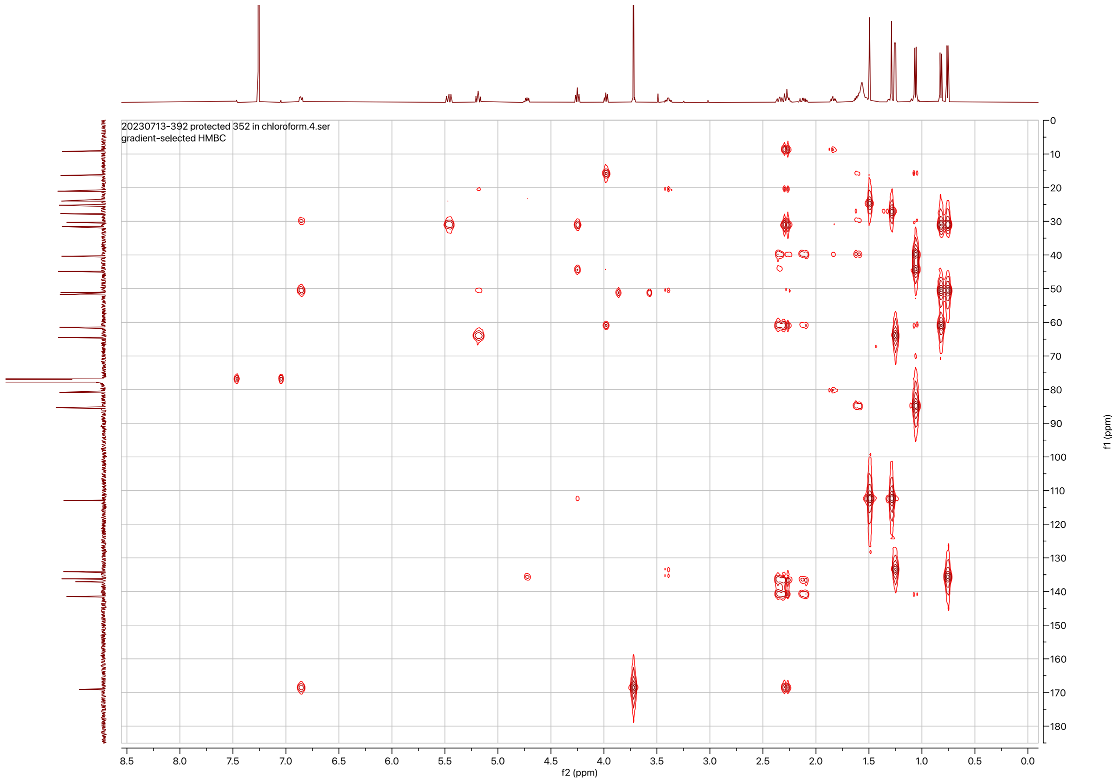

Figure S59. ^{1}H-^{13}C HMBC spectrum of compound **7b** in $CDCl_3$ (500 MHz)

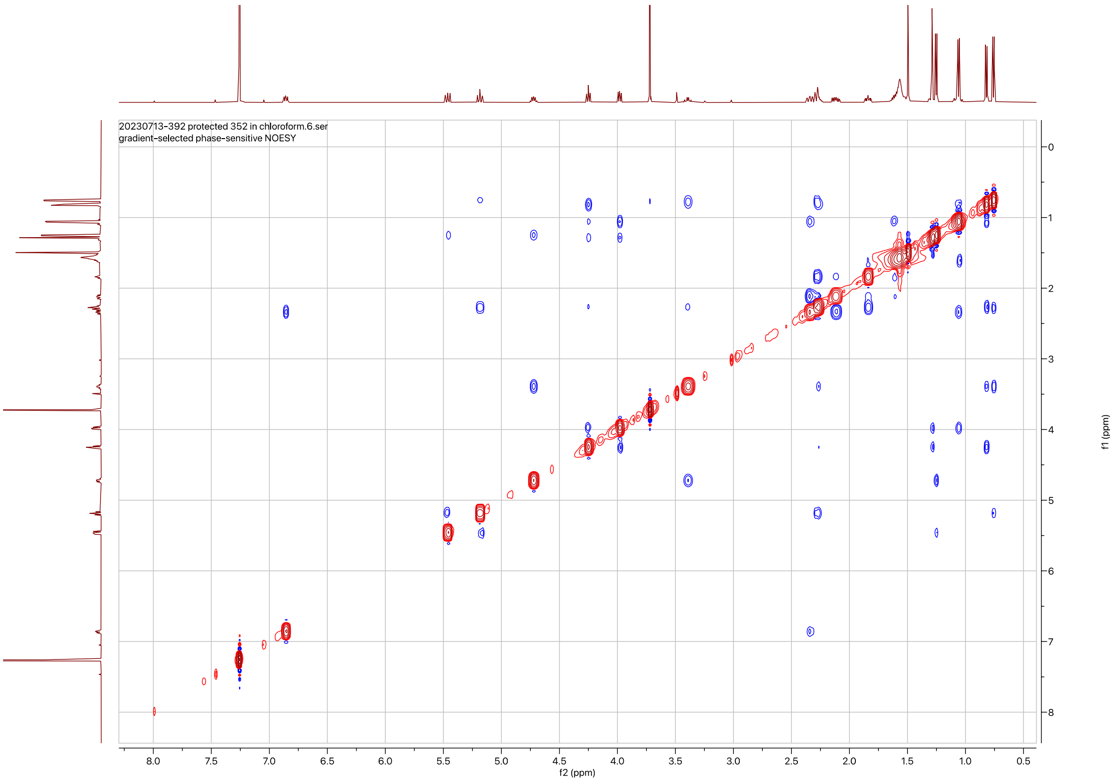

Figure S60. ^{1}H-^{1}H NOESY spectrum of compound **7b** in $CDCl_3$ (500 MHz)

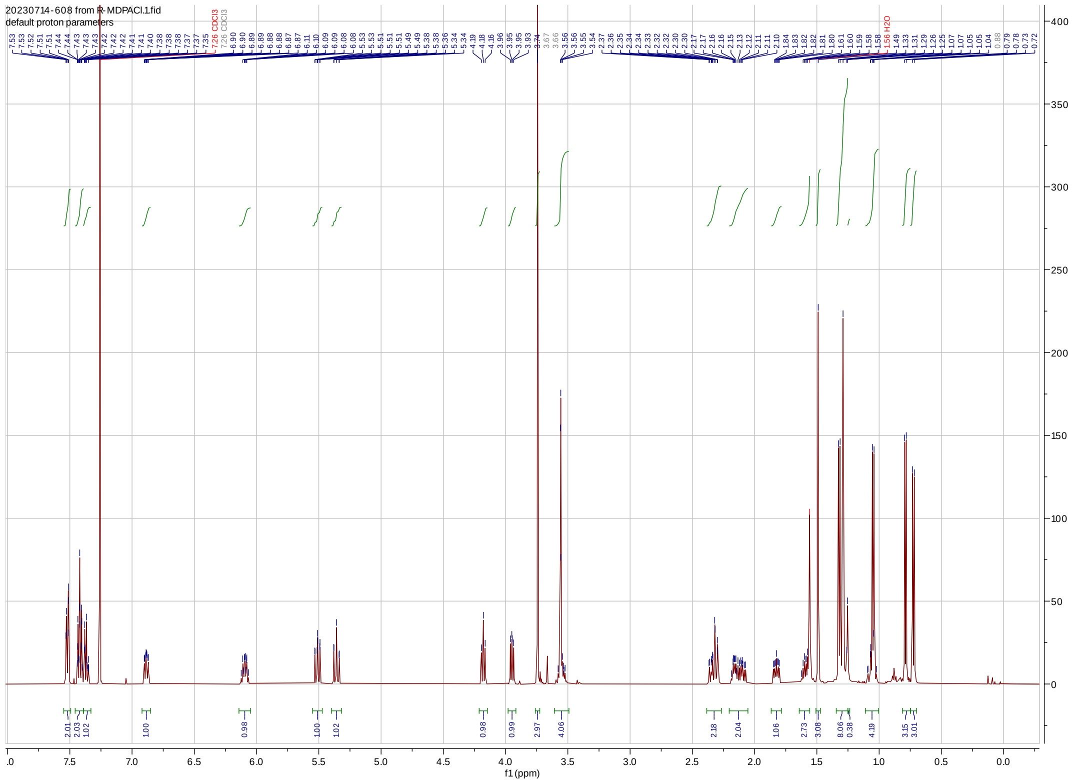

Figure S61. ^{1}H NMR spectrum of compound **7c** in $CDCl_3$ (500 MHz)

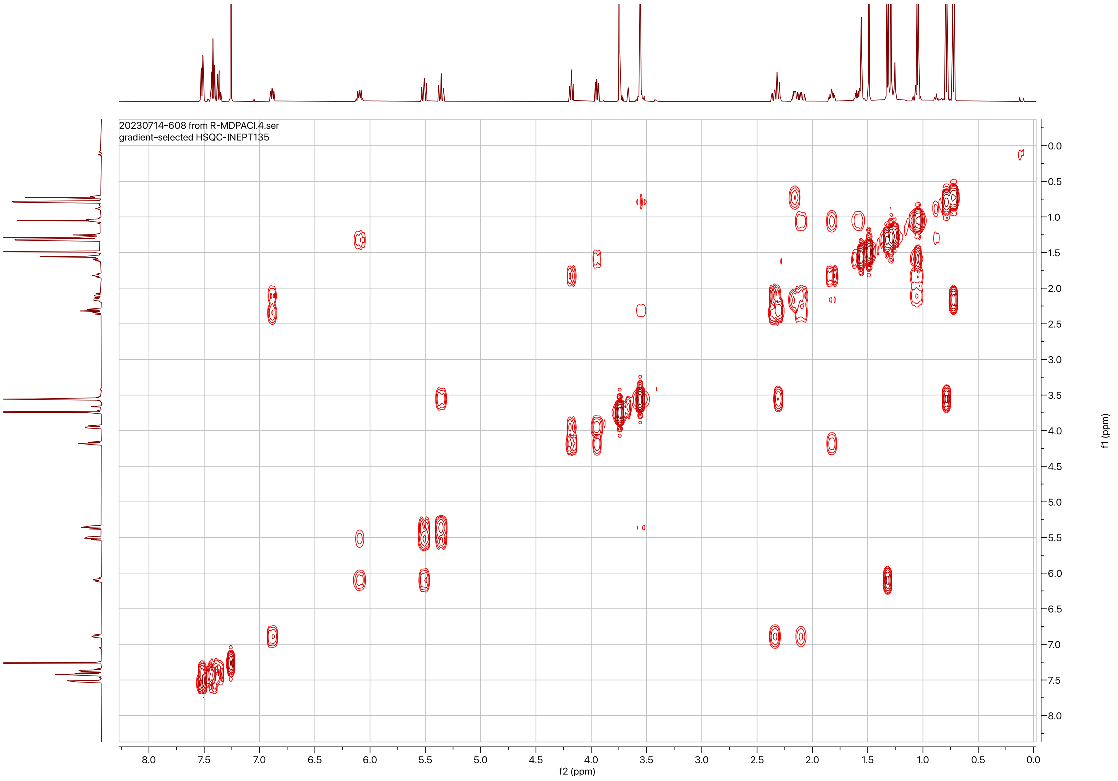

Figure S62. ^{1}H-^{1}H COSY spectrum of compound **7c** in $CDCl_3$ (500 MHz)

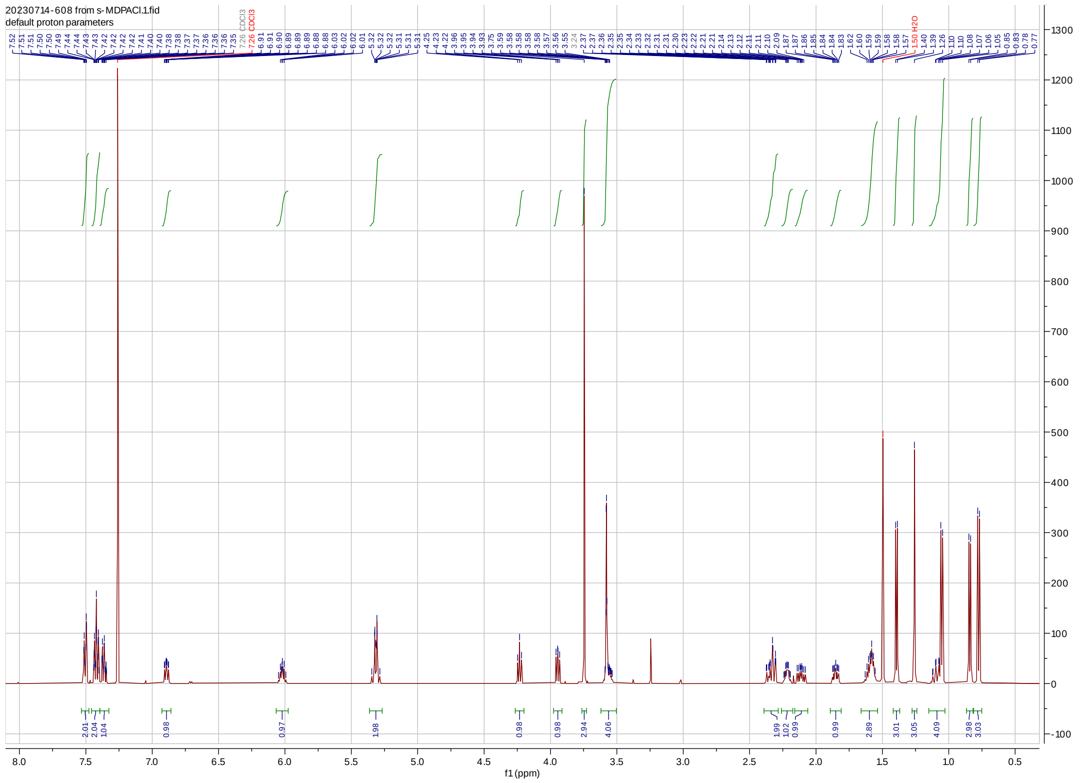

Figure S63. ^{1}H NMR spectrum of compound **7d** in $CDCl_3$ (500 MHz)

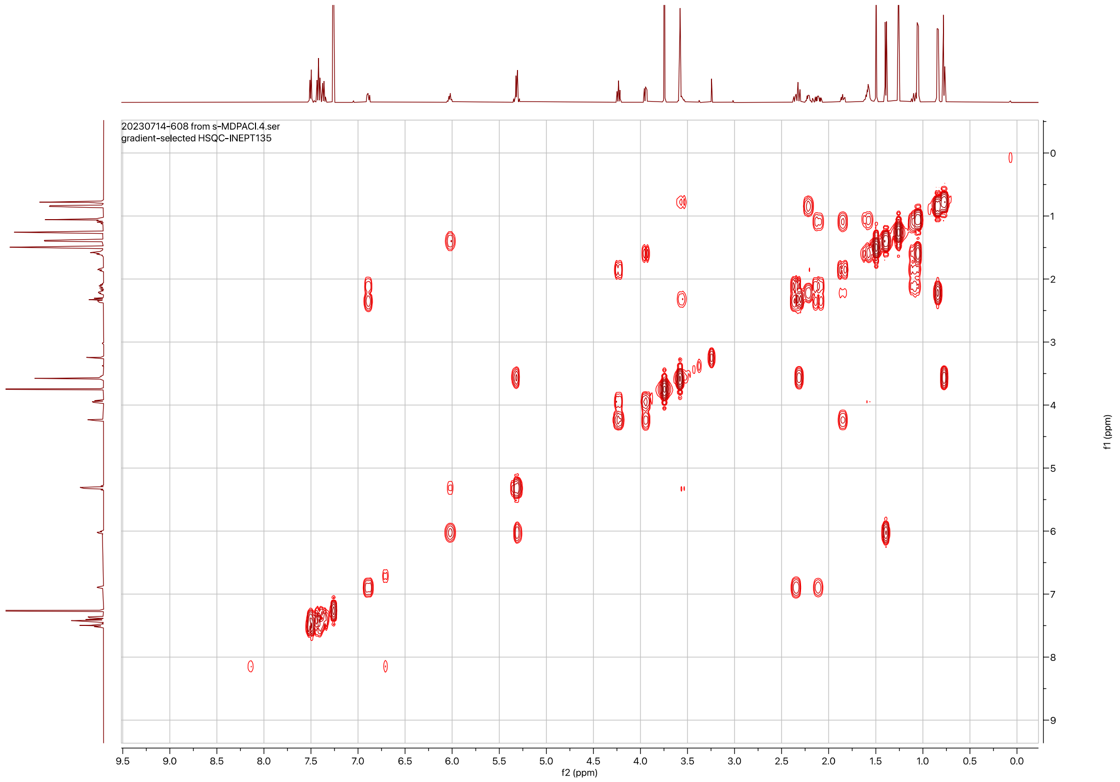

Figure S64. ^{1}H-^{1}H COSY spectrum of compound **7d** in $CDCl_3$ (500 MHz)

Supplementary Data

Data 1. Crystal data and structure refinement for compound 3 microED structure.

Type of instrument	Talos Arctica F200C
Wavelength	0.0215 Å
Data collection temperature	80 K
Unit cell dimensions	a = 9.4900 (10)
	b = 13.250 (2)
	c = 14.680 (4)
Volume	1845.9 (6)
Z	4
Crystal system	Orthorhombic
Space group	$P2_12_12_1$
Density (calculated)	1.160 Mg/m^3
F(000)	93
Measured reflections	1766
Reflections with I > 2s(*I*)	1083
Resolution	1.0 Å
Completeness	89.4%
Index ranges	9 ≤ h ≤ -9, 11 ≤ k ≤ -11, 14 ≤ l ≤ -14

Structure Solution and Refinement

Structure solution program	SHELXD (Uson & Sheldrick, 1999)
Primary solution method	Direct methods
Hydrogen placement	Riding
Structure refinement program	SHELXL-2018/3 (Sheldrick, 2018)
Refinement method	Full matrix least-squares on F^2
Data / restraints / parameters	1766 / 192 / 211
Goodness-of-fit on F^2	1.105
Final R indices [I>2s(I)]	R1 = 0.1224, *w*R2 = 0.3107
R indices (all data)	R1 = 0.1826, *w*R2 = 0.3459
Type of weighting scheme used	Sigma
Weighting scheme used	$w=1/s^2(Fo^2)$
Max shift/error	0.000
Average shift/error	0.000
Largest diff. peak and hole	0.14 and -0.11 e.Å^{-3}

Data 2. Cartesian Coordinates and Energies

For all minimum structures, no imaginary frequency was observed. Energies are reported in this section directly from the output file at the optimization level of theory (ωB97X-D /def2-SVP/IEEPCM(water)). E_SP, H and G are energies combining final single point energy with thermal corrections. (ωB97X-D/def2-QZVPP/SMD(water)). All the energies here are in Hartree.

11'

E=-1079.914612

E_SP=-1081.166849

H=-1080.67007

G=-1080.750823

Imag. Freq. 0

Cartesian coordinates

C	-1.359299	0.228753	2.614811
C	-0.782231	1.561264	2.248241
C	-1.159860	2.319442	1.214728
C	-0.557356	3.649320	0.865159
C	0.219952	3.652451	-0.470959
C	0.728982	5.059058	-0.796617
C	1.351811	2.666900	-0.396958
C	1.473776	1.523674	-1.081300
C	2.585317	0.575086	-0.774798
C	2.540551	-0.251368	0.437733
C	3.207258	-1.615458	0.504911
C	2.358194	-2.697766	-0.185714
C	0.927156	-2.617775	0.258140
C	-0.087839	-2.254976	-0.541854
C	-1.452319	-2.088421	-0.074058
C	-2.453806	-1.595233	-0.823677
C	-3.799020	-1.395753	-0.249767
O	-4.655656	-0.931456	-1.161895
C	-5.984909	-0.682557	-0.725695
O	-4.111407	-1.607409	0.902404
C	2.949974	-4.089748	0.049027

O	4.463490	-1.576881	-0.117648
O	3.426251	0.852106	0.338515
C	0.495809	1.018858	-2.110489
H	-2.196720	-0.054399	1.960933
H	-1.721029	0.227964	3.655897
H	-0.593028	-0.562226	2.551524
H	0.022504	1.934413	2.896320
H	-1.953687	1.951997	0.549185
H	0.121583	3.973308	1.673231
H	-1.354369	4.410561	0.797500
H	-0.482768	3.347312	-1.263160
H	1.246652	5.078088	-1.767317
H	1.439624	5.405091	-0.028225
H	-0.101433	5.780909	-0.836887
H	2.124864	2.891493	0.348655
H	3.123185	0.160445	-1.638376
H	1.693970	-0.092705	1.117084
H	3.311923	-1.880893	1.577731
H	2.398946	-2.468200	-1.263818
H	0.721260	-2.844454	1.313062
H	0.113525	-2.024129	-1.593984
H	-1.668725	-2.350690	0.967437
H	-2.307582	-1.317338	-1.870418
H	-6.452957	-1.605089	-0.354215
H	-6.530209	-0.310489	-1.599258
H	-5.997514	0.070326	0.075171
H	2.908044	-4.358309	1.116624
H	4.000889	-4.114619	-0.270325
H	2.394866	-4.851118	-0.517601
H	4.839953	-0.707895	0.080216

H	0.026198	1.834329	-2.676549
H	-0.309778	0.443247	-1.625604
H	0.987526	0.347692	-2.831027

7'

E=-1156.345332

E_SP=-1157.694458

H=-1157.16351

G=-1157.240819

Imag. Freq. 0

Cartesian coordinates

C	5.703156	-1.546905	0.015654
C	4.618005	-0.811711	-0.769485
C	3.397849	-0.559675	0.084453
C	2.779470	0.615764	0.205294
C	1.581838	0.886790	1.077837
C	1.970453	1.903610	2.158261
C	0.386760	1.359752	0.202962
C	-0.031839	0.302040	-0.853230
C	-1.163079	-0.637245	-0.416981
C	-1.373503	-1.828436	-1.375672
C	-1.532674	-3.073892	-0.466094
C	-1.942925	-2.511456	0.897506
C	-1.053639	-1.259614	0.989136
C	-1.425640	-0.331283	2.108561
C	-1.343031	1.002343	2.123243
C	-0.772669	1.921768	1.069893
C	-1.857242	2.562317	0.216391

O	-1.391370	3.645943	-0.406385
C	-2.285784	4.326504	-1.276663
O	-2.996076	2.179262	0.097937
C	-1.815510	-3.510146	2.034932
O	-2.402735	-4.020756	-1.013368
O	-2.505788	-1.688035	-2.217837
C	-0.373148	0.947781	-2.200287
H	5.328776	-2.504579	0.406500
H	6.036785	-0.938358	0.872090
H	6.569659	-1.747776	-0.630441
H	4.309717	-1.456712	-1.611511
H	3.014651	-1.431118	0.630615
H	0.854431	-0.331299	-1.020286
H	3.163718	1.472904	-0.363272
H	1.307737	-0.051837	1.583265
H	1.190380	2.007630	2.926299
H	2.158729	2.897497	1.719178
H	2.890867	1.581795	2.666709
H	0.765555	2.230537	-0.358745
H	-2.105424	-0.061164	-0.415870
H	-0.517871	-1.961629	-2.054331
H	-0.539214	-3.544584	-0.345268
H	-2.995999	-2.180823	0.816292
H	-0.017225	-1.618369	1.148787
H	-1.826564	-0.812252	3.007771
H	-1.707677	1.515755	3.019262
H	-0.345061	2.782938	1.605531
H	-3.167873	4.683141	-0.727268
H	-2.615337	3.663918	-2.090019
H	-1.728315	5.174612	-1.687170

H	-2.154776	-3.093953	2.995349
H	-0.767740	-3.831652	2.154508
H	-2.422607	-4.403259	1.824615
H	-2.933209	-3.525117	-1.656844
H	0.455698	1.577143	-2.556877
H	-1.273480	1.577870	-2.135994
H	-0.574400	0.186101	-2.967401
H	-3.172647	-1.165344	-1.758589
O	5.118568	0.361034	-1.370893
H	5.436849	0.938573	-0.666786

H_2O

E=-76.343847

E_SP=-76.458684

H=-76.433266

G=-76.45168

Imag. Freq. 0

Cartesian coordinates

O	0.000000	0.000000	0.120080
H	-0.000000	0.752965	-0.480319
H	-0.000000	-0.752965	-0.480319

IM1

E=-1080.339791

E_SP=-1081.608032

H=-1081.097934

G=-1081.177637

Imag. Freq. 0

Cartesian coordinates

C	-2.629091	4.363918	-0.102751
C	-1.276695	3.997105	0.422575
C	-0.145382	3.999740	-0.288677
C	1.193594	3.581110	0.238392
C	1.682113	2.191997	-0.299810
C	3.138563	1.939709	0.086802
C	0.758461	1.187444	0.278835
C	-0.436510	0.746024	-0.259957
C	-1.267379	0.074507	0.653406
C	-2.732423	-0.165813	0.506263
C	-3.124888	-1.249418	-0.530450
C	-2.416126	-2.581946	-0.186230
C	-0.947226	-2.330156	-0.144001
C	-0.180239	-2.421218	0.971372
C	1.245174	-2.160775	1.035547
C	2.017623	-1.696794	0.032275
C	3.477520	-1.530239	0.248603
O	4.105145	-1.260098	-0.895576
C	5.516439	-1.085685	-0.828406
O	4.039565	-1.631584	1.313185
C	-2.784312	-3.663448	-1.203409
O	-4.507682	-1.367758	-0.567021
O	-3.316178	-0.564338	1.715542
C	-0.878177	1.018493	-1.670905
H	-2.591630	4.635335	-1.167739
H	-3.055669	5.211338	0.457552
H	-3.333978	3.524941	0.015620

H	-1.229039	3.698328	1.478365
H	-0.182091	4.286895	-1.347580
H	1.190300	3.558618	1.340332
H	1.965497	4.305822	-0.065934
H	1.596791	2.211815	-1.395911
H	3.254101	1.837163	1.176685
H	3.766203	2.777748	-0.246803
H	3.513373	1.028765	-0.395628
H	0.975514	0.897193	1.313343
H	-0.895493	-0.059111	1.672203
H	-3.172559	0.781564	0.136542
H	-2.802962	-0.922856	-1.530829
H	-2.758523	-2.885004	0.816001
H	-0.471518	-2.102288	-1.105769
H	-0.662855	-2.711937	1.909634
H	1.733813	-2.338835	1.997896
H	1.644751	-1.511376	-0.977396
H	5.999360	-1.992190	-0.438578
H	5.768449	-0.235361	-0.178735
H	5.848769	-0.889698	-1.852619
H	-3.876226	-3.771249	-1.246099
H	-2.345227	-4.630740	-0.923396
H	-2.425094	-3.397717	-2.209936
H	-4.800259	-1.466005	0.350238
H	-0.848347	0.096780	-2.272007
H	-0.238224	1.755406	-2.165877
H	-1.907439	1.404959	-1.697103
H	-3.492826	0.204588	2.267646

IM2

E=-1080.374123

E_SP=-1081.636882

H=-1081.124422

G=-1081.199164

Imag. Freq. 0

Cartesian coordinates

C	1.984608	3.851943	0.530237
C	0.868266	3.324874	-0.314335
C	-0.389485	3.138518	0.124373
C	-1.552643	2.772092	-0.733421
C	-2.234279	1.442164	-0.357533
C	-3.411324	1.237318	-1.312685
C	-1.177172	0.323297	-0.482457
C	0.073318	0.486285	0.305721
C	1.300363	-0.004972	-0.321043
C	2.695916	0.441949	0.181212
C	3.419521	-0.848282	0.622173
C	2.786347	-1.948086	-0.237870
C	1.298655	-1.616492	-0.137972
C	0.372265	-2.311805	-1.077923
C	-0.953843	-2.136887	-1.032614
C	-1.585514	-1.191005	-0.060966
C	-3.082694	-1.417099	0.094507
O	-3.534061	-0.805907	1.180269
C	-4.927528	-0.918985	1.461691
O	-3.756909	-2.097294	-0.634144
C	3.138769	-3.357980	0.205524

O	4.802794	-0.737581	0.536110
O	3.444005	0.959267	-0.888486
C	0.022442	0.678251	1.773204
H	1.742774	3.816831	1.602035
H	2.180271	4.903204	0.262487
H	2.935198	3.321800	0.367656
H	1.074556	3.164380	-1.380751
H	-0.607434	3.334541	1.181474
H	-1.242480	2.729850	-1.790325
H	-2.312210	3.566794	-0.655661
H	-2.607212	1.494385	0.676703
H	-3.056529	1.187641	-2.353878
H	-4.096882	2.092360	-1.232909
H	-4.001651	0.335125	-1.123725
H	-0.915914	0.264292	-1.546659
H	1.269311	0.167205	-1.404142
H	2.637803	1.166858	1.006899
H	3.165745	-1.051643	1.677067
H	3.112536	-1.792781	-1.281580
H	0.983880	-1.823355	0.899554
H	0.810987	-2.947460	-1.852272
H	-1.613822	-2.619559	-1.756593
H	-1.204959	-1.380559	0.952732
H	-5.212674	-1.973633	1.569406
H	-5.518232	-0.462178	0.655251
H	-5.091675	-0.380934	2.399976
H	4.229717	-3.491385	0.180332
H	2.686311	-4.116089	-0.450043
H	2.794126	-3.543317	1.235413
H	4.976669	-0.170816	-0.229347

H	0.300364	-0.274807	2.257253
H	-0.965014	0.980943	2.136140
H	0.784509	1.396256	2.104072
H	3.239388	1.887589	-1.035490

IM3

E=-1080.404034

E_SP=-1081.656072

H=-1081.141417

G=-1081.21471

Imag. Freq. 0

Cartesian coordinates

C	-6.281484	-0.224630	-0.066130
C	-4.938575	-0.599852	-0.613138
C	-3.764061	-0.226231	-0.099264
C	-2.419060	-0.595037	-0.654041
C	-1.540369	-1.351742	0.355587
C	-2.197587	-2.665903	0.784509
C	-0.128142	-1.652261	-0.178665
C	0.694869	-0.619462	-1.000276
C	0.940312	0.774564	-0.380198
C	0.463856	2.005364	-1.177541
C	0.449747	3.165044	-0.119760
C	0.826460	2.532278	1.224447
C	0.436851	1.056434	1.044925
C	0.911480	0.142251	2.138901
C	1.079956	-1.184443	2.102687
C	0.884697	-2.097408	0.899864

C	2.135202	-2.045514	0.094233
O	3.195482	-2.667360	0.394122
C	4.387196	-2.501917	-0.415242
O	2.067593	-1.291047	-0.921341
C	0.213205	3.241435	2.421324
O	1.377196	4.170073	-0.466449
O	1.281331	2.315319	-2.265202
C	0.392962	-0.604739	-2.483470
H	-6.863294	-1.122126	0.199372
H	-6.874767	0.323083	-0.816193
H	-6.193001	0.406252	0.830551
H	-4.937880	-1.233412	-1.510104
H	-3.752682	0.402964	0.801675
H	-1.906795	0.336411	-0.946651
H	-2.539183	-1.195912	-1.571375
H	-1.456372	-0.729566	1.257636
H	-3.191351	-2.475562	1.212239
H	-1.603340	-3.190628	1.546537
H	-2.324914	-3.341120	-0.077006
H	-0.238747	-2.512767	-0.860726
H	2.035827	0.887219	-0.355643
H	-0.562279	1.847260	-1.547721
H	-0.563780	3.590685	-0.060131
H	1.926981	2.584186	1.294026
H	-0.668067	1.029578	1.046406
H	1.100153	0.622063	3.104781
H	1.423710	-1.692469	3.006459
H	0.718019	-3.124838	1.246820
H	4.171953	-2.820810	-1.441349
H	5.138284	-3.144467	0.048507

H 4.693852 -1.450235 -0.387917

H -0.886309 3.177134 2.394730

H 0.493958 4.304942 2.413709

H 0.557013 2.815423 3.375150

H 0.912399 4.909348 -0.871581

H -0.611985 -0.203551 -2.656961

H 0.432318 -1.624007 -2.889649

H 1.107369 0.038337 -3.010524

H 1.804996 3.081319 -1.976624

IM4

E=-1080.386175

E_SP=-1081.648428

H=-1081.135166

G=-1081.211057

Imag. Freq. 0

Cartesian coordinates

C -6.001749 0.049797 -0.902950

C -4.778856 -0.748607 -0.900960

C -3.750121 -0.636439 0.017566

C -2.644554 -1.455256 -0.111496

C -1.506867 -1.490493 0.827813

C -1.751323 -2.729490 1.724902

C -0.167032 -1.548549 0.052631

C -0.028898 -0.365448 -0.947145

C 0.686107 0.869436 -0.385970

C 0.594083 2.101264 -1.305923

C 0.321873 3.305811 -0.363617

C	0.735701	2.820854	1.029049
C	0.259657	1.358493	1.011072
C	0.798374	0.543793	2.152163
C	1.152690	-0.744797	2.145912
C	1.021071	-1.748959	1.026327
C	2.331639	-1.958414	0.276951
O	2.286604	-3.077623	-0.444090
C	3.432148	-3.390229	-1.227667
O	3.297715	-1.238043	0.318838
C	0.204646	3.686827	2.158603
O	0.954310	4.474645	-0.795873
O	1.821752	2.288230	-1.991205
C	0.623015	-0.811582	-2.259409
H	-6.059008	0.771143	-0.080854
H	-6.866549	-0.636365	-0.882442
H	-6.079602	0.562897	-1.877884
H	-4.671152	-1.493370	-1.698966
H	-3.805962	0.093112	0.830000
H	-1.056902	-0.043855	-1.202077
H	-2.627526	-2.171351	-0.944673
H	-1.550023	-0.601843	1.471715
H	-2.764560	-2.721570	2.148897
H	-1.044835	-2.704570	2.565429
H	-1.608821	-3.664031	1.163083
H	-0.208251	-2.470145	-0.550905
H	1.761944	0.641804	-0.317116
H	-0.224379	1.997243	-2.036146
H	-0.764396	3.503064	-0.346139
H	1.841689	2.816449	1.058467
H	-0.847231	1.385654	1.075815

H	0.929555	1.085390	3.095352
H	1.573481	-1.154284	3.069704
H	0.853910	-2.729495	1.499261
H	3.204450	-4.329842	-1.740926
H	4.317615	-3.511169	-0.588922
H	3.626159	-2.596259	-1.963021
H	-0.897659	3.676798	2.172687
H	0.532991	4.727874	2.021958
H	0.560284	3.351276	3.144233
H	1.783030	4.175500	-1.198401
H	0.594906	-0.007304	-3.007654
H	0.104933	-1.684446	-2.682867
H	1.680967	-1.076654	-2.117336
H	1.638268	2.582614	-2.888738

TS1

E=-1080.339059

E_SP=-1081.60671

H=-1081.097367

G=-1081.175784

Imag. Freq. -173.18

Cartesian coordinates

C	-2.663651	4.296842	-0.042790
C	-1.290247	3.957022	0.446531
C	-0.182527	3.957510	-0.300541
C	1.180594	3.569357	0.188871
C	1.668815	2.189892	-0.350355
C	3.136599	1.952832	0.003087

C	0.774620	1.151603	0.238786
C	-0.391396	0.663145	-0.291112
C	-1.207984	-0.089683	0.611587
C	-2.710684	-0.189872	0.532634
C	-3.189819	-1.213220	-0.512431
C	-2.416612	-2.522084	-0.243353
C	-0.968416	-2.158553	-0.150710
C	-0.193836	-2.369845	0.961837
C	1.223983	-2.125435	1.057608
C	2.008266	-1.687608	0.050206
C	3.465874	-1.490944	0.287386
O	4.112670	-1.288300	-0.855844
C	5.521857	-1.094613	-0.772217
O	3.996631	-1.520993	1.370841
C	-2.686666	-3.568043	-1.322729
O	-4.568409	-1.363490	-0.466164
O	-3.257876	-0.604999	1.753798
C	-0.863088	0.936166	-1.692526
H	-2.663811	4.542196	-1.114845
H	-3.081325	5.154034	0.509512
H	-3.356745	3.455090	0.118441
H	-1.204862	3.682778	1.506608
H	-0.259188	4.219250	-1.364199
H	1.206036	3.553226	1.291145
H	1.924476	4.313967	-0.138098
H	1.568291	2.205340	-1.445671
H	3.276829	1.847482	1.090152
H	3.750878	2.797527	-0.339577
H	3.512442	1.046994	-0.489142
H	1.003208	0.885145	1.277131

H -0.845741 -0.148243 1.640436

H -3.085556 0.809344 0.240889

H -2.935539 -0.848963 -1.519524

H -2.751504 -2.903089 0.734310

H -0.477404 -1.905579 -1.096366

H -0.691491 -2.726276 1.869097

H 1.690737 -2.281300 2.033659

H 1.653603 -1.552140 -0.973270

H 6.003112 -1.967850 -0.311315

H 5.750143 -0.199222 -0.176817

H 5.872876 -0.965151 -1.800500

H -3.769746 -3.734532 -1.400812

H -2.202198 -4.524057 -1.081552

H -2.318921 -3.229026 -2.303683

H -4.802232 -1.456320 0.468414

H -0.909444 0.009124 -2.284657

H -0.200959 1.629508 -2.220208

H -1.870030 1.380207 -1.689563

H -3.344144 0.145983 2.350358

TS1_endo1

E=-1080.333504

E_SP=-1081.598276

H=-1081.088771

G=-1081.166106

Imag. Freq. -280.97

Cartesian coordinates

C -2.806790 3.960563 -0.420257

C	-1.473633	3.727606	0.216854
C	-0.292900	3.813126	-0.402137
C	1.029872	3.498729	0.229572
C	1.644485	2.150731	-0.263119
C	3.088612	1.996703	0.212700
C	0.751250	1.066090	0.237451
C	-0.348035	0.546526	-0.402245
C	-1.223598	-0.255845	0.386482
C	-2.730931	-0.309017	0.167334
C	-3.247394	-1.626979	0.736995
C	-2.445272	-2.761360	0.082805
C	-0.988452	-2.333565	0.078161
C	-0.179818	-2.400081	-1.040920
C	1.219045	-2.099756	-1.069218
C	1.918411	-1.657976	0.003158
C	3.377424	-1.379876	-0.097434
O	3.892906	-1.149113	1.102726
C	5.288943	-0.874459	1.166329
O	4.007916	-1.371765	-1.126993
C	-3.016034	-3.157933	-1.275736
O	-3.017945	-1.529732	2.124305
O	-3.299472	0.792005	0.812655
C	-0.659522	0.767207	-1.848633
H	-2.710015	4.221614	-1.484471
H	-3.356357	4.769754	0.087579
H	-3.420437	3.050472	-0.329632
H	-1.488019	3.438544	1.275981
H	-0.268812	4.079536	-1.467189
H	0.941140	3.475212	1.328396
H	1.763535	4.283299	-0.016579

H	1.637387	2.171840	-1.362868
H	3.143232	1.819181	1.297694
H	3.658818	2.908909	-0.012986
H	3.584275	1.166724	-0.307436
H	0.881756	0.809527	1.295705
H	-0.964439	-0.320534	1.447956
H	-2.972748	-0.239777	-0.901527
H	-4.323798	-1.731406	0.523420
H	-2.514442	-3.632549	0.754578
H	-0.500356	-2.372426	1.055635
H	-0.634152	-2.652123	-2.002793
H	1.739849	-2.183275	-2.026377
H	1.487791	-1.600707	1.003816
H	5.524185	0.045217	0.612050
H	5.865117	-1.708408	0.743382
H	5.525921	-0.746348	2.226793
H	-4.066396	-3.459446	-1.160723
H	-2.471513	-4.012157	-1.701650
H	-2.985909	-2.336738	-2.007811
H	-3.551694	-2.182372	2.588374
H	0.109095	1.354051	-2.361345
H	-1.621445	1.286052	-1.972116
H	-0.744416	-0.204815	-2.364988
H	-3.368369	0.549362	1.748733

TS1_endo2

E=-1080.334984

E_SP=-1081.600324

H=-1081.091013

G=-1081.168402

Imag. Freq. -255.04

Cartesian coordinates

C	-6.719337	-1.284027	-0.213206
C	-5.357746	-1.313032	-0.835728
C	-4.266562	-0.731422	-0.330282
C	-2.902819	-0.749074	-0.956394
C	-1.835262	-1.344888	-0.020931
C	-2.020074	-2.864396	0.135778
C	-0.451134	-1.042426	-0.505204
C	0.703990	-1.169170	0.224718
C	1.925220	-0.928682	-0.478328
C	3.285915	-1.490035	-0.166438
C	4.325235	-0.596316	-0.855722
C	4.077832	0.842326	-0.350917
C	2.591373	1.076094	-0.240202
C	2.013792	1.397622	0.974172
C	0.635698	1.716423	1.185025
C	-0.283060	1.800717	0.192715
C	-1.713198	2.074589	0.509506
O	-2.322968	2.648469	-0.518676
C	-3.700442	2.984708	-0.359731
O	-2.243346	1.806538	1.560755
C	4.773666	1.880800	-1.226259
O	5.623631	-1.035586	-0.617733
O	3.626038	-1.538944	1.190674
C	0.701972	-1.476316	1.691349
H	-6.721485	-0.722519	0.732526
H	-7.081261	-2.304928	-0.010598

H	-7.452975	-0.820061	-0.892126
H	-5.266893	-1.858012	-1.784688
H	-4.344293	-0.191460	0.624042
H	-2.614404	0.280555	-1.225335
H	-2.920851	-1.317291	-1.901425
H	-1.955771	-0.884325	0.975785
H	-1.852522	-3.376323	-0.823987
H	-3.048277	-3.076643	0.462039
H	-1.327634	-3.284955	0.877673
H	-0.350497	-0.801204	-1.570269
H	1.789187	-0.803477	-1.557624
H	3.313026	-2.493520	-0.632013
H	4.161676	-0.637209	-1.944909
H	4.500134	0.884047	0.665763
H	2.067263	1.366657	-1.156780
H	2.642178	1.332462	1.867885
H	0.295594	1.829380	2.217581
H	-0.009919	1.797773	-0.864498
H	-3.845131	3.600953	0.537348
H	-4.306644	2.071566	-0.279443
H	-3.980789	3.543923	-1.257310
H	4.686530	2.889520	-0.798938
H	4.343443	1.894434	-2.239756
H	5.840679	1.630326	-1.309387
H	5.686630	-1.196421	0.334171
H	-0.294354	-1.354819	2.132398
H	1.406231	-0.822807	2.225564
H	1.026870	-2.511585	1.877218
H	3.437051	-2.408820	1.555414

TS1_trans

E=-1080.330435

E_SP=-1081.601607

H=-1081.092515

G=-1081.172276

Imag. Freq. -234.71

Cartesian coordinates

C	-3.766626	4.854367	-1.412279
C	-3.823819	3.761404	-0.389692
C	-3.135778	2.617474	-0.440610
C	-3.188033	1.536862	0.600847
C	-1.853731	1.337350	1.340230
C	-1.978303	0.269553	2.441278
C	-0.764049	0.933432	0.395944
C	0.566892	1.171687	0.527566
C	1.419611	0.612751	-0.507598
C	2.734585	1.233293	-0.928051
C	3.898536	0.787874	-0.023497
C	3.784794	-0.745556	0.109563
C	2.352975	-1.021868	0.458330
C	1.500464	-1.793707	-0.296406
C	0.175238	-2.164578	0.138798
C	-0.678235	-2.806120	-0.677249
C	-2.061211	-3.136586	-0.226911
O	-2.713287	-3.815105	-1.156737
C	-4.057415	-4.188283	-0.862531
O	-2.525127	-2.820036	0.842412
C	4.760152	-1.306304	1.140883

O	5.122251	1.227660	-0.506850
O	3.037474	0.997636	-2.276793
C	1.174925	1.945456	1.665008
H	-3.441045	5.803485	-0.956489
H	-4.761961	5.040885	-1.847166
H	-3.073543	4.609575	-2.230577
H	-4.493997	3.929498	0.464146
H	-2.463179	2.445520	-1.292621
H	-3.470427	0.575524	0.134015
H	-3.967882	1.769027	1.342808
H	-1.584238	2.293353	1.817087
H	-1.037906	0.169898	3.003410
H	-2.232047	-0.711474	2.011625
H	-2.772021	0.552642	3.148474
H	-1.086438	0.342949	-0.470132
H	0.868085	0.212140	-1.363246
H	2.606957	2.324896	-0.840131
H	3.764824	1.230190	0.976654
H	4.012392	-1.196619	-0.871382
H	2.053880	-0.806583	1.490399
H	1.819683	-2.130200	-1.289068
H	-0.143979	-1.893543	1.149369
H	-0.394505	-3.104543	-1.689590
H	-4.422345	-4.727852	-1.741584
H	-4.671022	-3.295386	-0.680647
H	-4.089548	-4.836632	0.023608
H	4.537032	-0.917520	2.146361
H	5.780837	-1.000827	0.873223
H	4.720192	-2.403459	1.174251
H	5.109264	1.110079	-1.466905

H	1.745021	1.286671	2.338998
H	0.408403	2.442142	2.269952
H	1.864471	2.721771	1.301071
H	3.078704	0.052168	-2.463908

TS2

E=-1080.368925

E_SP=-1081.624647

H=-1081.115511

G=-1081.189579

Imag. Freq. -176.78

Cartesian coordinates

C	-5.269591	-2.189439	0.134230
C	-3.894001	-2.286143	-0.427375
C	-2.772686	-1.905333	0.206256
C	-1.436044	-2.040165	-0.393779
C	-0.225395	-2.275166	0.504652
C	0.013464	-3.742847	0.822366
C	0.902601	-1.568651	-0.267087
C	0.165098	-0.493719	-1.066794
C	-0.046304	0.863025	-0.474389
C	-1.098842	1.739503	-1.190395
C	-1.714801	2.619064	-0.060008
C	-0.792904	2.458753	1.145247
C	-0.337971	0.992033	1.030745
C	0.773933	0.637552	1.973983
C	1.773519	-0.232014	1.808339
C	2.075418	-1.118207	0.616236

C	3.193936	-0.475904	-0.192340
O	4.346589	-0.527358	0.453433
C	5.466896	0.106842	-0.161043
O	3.051705	0.047666	-1.272346
C	-1.443770	2.855394	2.459668
O	-1.805705	3.964886	-0.460087
O	-0.519029	2.523181	-2.189711
C	0.261535	-0.576685	-2.552596
H	-5.274435	-1.758450	1.144488
H	-5.739320	-3.185127	0.167880
H	-5.901910	-1.569317	-0.521128
H	-3.804661	-2.709030	-1.435765
H	-2.828257	-1.472600	1.211538
H	-1.184252	-0.965158	-0.885465
H	-1.425883	-2.663625	-1.300687
H	-0.392921	-1.734662	1.448506
H	0.183804	-4.323493	-0.096798
H	-0.853255	-4.170455	1.346781
H	0.889507	-3.862559	1.475750
H	1.315260	-2.277603	-1.001104
H	0.930799	1.345944	-0.674494
H	-1.890050	1.101520	-1.627740
H	-2.710438	2.223645	0.198496
H	0.088616	3.099567	0.961529
H	-1.218559	0.368117	1.281720
H	0.737336	1.155077	2.937597
H	2.494425	-0.332231	2.624246
H	2.508286	-2.043443	1.028495
H	6.306870	-0.028949	0.526948
H	5.266888	1.176277	-0.311692

H	5.689176	-0.361374	-1.129295
H	-2.293843	2.193835	2.691609
H	-1.820807	3.886130	2.391857
H	-0.737780	2.814809	3.301469
H	-2.703579	4.155530	-0.750663
H	0.153062	-1.607632	-2.912493
H	1.294563	-0.257240	-2.770391
H	-0.429419	0.095741	-3.072899
H	-0.802255	3.432387	-1.994116

Trans_int

E=-1080.335565

E_SP=-1081.605443

H=-1081.095948

G=-1081.174288

Imag. Freq. 0

Cartesian coordinates

C	-1.655204	6.034371	-1.196745
C	-2.310028	4.936567	-0.415191
C	-2.050913	3.632630	-0.547509
C	-2.701514	2.539603	0.250074
C	-1.719673	1.771856	1.154383
C	-2.447428	0.709151	1.987896
C	-0.635177	1.122570	0.338167
C	0.657450	0.953782	0.669305
C	1.542741	0.212532	-0.289861
C	2.943905	0.802449	-0.590519
C	3.962735	-0.073115	0.152453

C	3.400362	-1.496345	0.001133
C	1.918774	-1.296975	0.313712
C	0.932410	-2.051577	-0.389353
C	-0.386888	-2.242661	0.089566
C	-1.313873	-2.816689	-0.714519
C	-2.724668	-2.999449	-0.239417
O	-3.458666	-3.578586	-1.166609
C	-4.833010	-3.812229	-0.854013
O	-3.107964	-2.650144	0.848323
C	4.083976	-2.534158	0.876545
O	5.264073	0.097933	-0.307360
O	3.240430	0.847082	-1.963882
C	1.273484	1.419202	1.960675
H	-1.146185	6.746756	-0.527294
H	-2.400555	6.615480	-1.763854
H	-0.913670	5.640123	-1.907248
H	-3.064861	5.247178	0.319929
H	-1.294317	3.318377	-1.279709
H	-3.180412	1.814238	-0.433604
H	-3.504755	2.960635	0.875641
H	-1.269679	2.503108	1.844534
H	-1.756018	0.224342	2.693551
H	-2.877130	-0.075905	1.346233
H	-3.265761	1.163101	2.567525
H	-0.969361	0.709149	-0.623149
H	1.022682	0.125862	-1.254321
H	2.983263	1.847050	-0.252046
H	3.945645	0.190719	1.224455
H	3.510167	-1.795305	-1.056882
H	1.721224	-1.254582	1.393347

H	1.172438	-2.414127	-1.397582
H	-0.661329	-1.911913	1.094406
H	-1.069006	-3.162312	-1.722640
H	-5.266367	-4.291410	-1.736337
H	-5.339853	-2.860631	-0.644784
H	-4.915051	-4.470895	0.020907
H	3.960788	-2.289924	1.943183
H	5.159631	-2.549819	0.651270
H	3.679784	-3.541448	0.702779
H	5.197101	0.303004	-1.250739
H	1.659200	0.572556	2.551282
H	0.552831	1.951013	2.592094
H	2.122880	2.096553	1.780056
H	3.078919	-0.010361	-2.375459

References

1. Frisch, M. J.; Trucks, G. W.; Schlegel, H. B.; Scuseria, G. E.; Robb, M. A.; Cheeseman, J. R.; Scalmani, G.; Barone, V.; Petersson, G. A.; Nakatsuji, H.; Li, X.; Caricato, M.; Marenich, A. V.; Bloino, J.; Janesko, B. G.; Gomperts, R.; Mennucci, B.; Hratchian, H. P.; Ortiz, J. V.; Izmaylov, A. F.; Sonnenberg, J. L.; Williams; Ding, F.; Lipparini, F.; Egidi, F.; Goings, J.; Peng, B.; Petrone, A.; Henderson, T.; Ranasinghe, D.; Zakrzewski, V. G.; Gao, J.; Rega, N.; Zheng, G.; Liang, W.; Hada, M.; Ehara, M.; Toyota, K.; Fukuda, R.; Hasegawa, J.; Ishida, M.; Nakajima, T.; Honda, Y.; Kitao, O.; Nakai, H.; Vreven, T.; Throssell, K.; Montgomery Jr., J. A.; Peralta, J. E.; Ogliaro, F.; Bearpark, M. J.; Heyd, J. J.; Brothers, E. N.; Kudin, K. N.; Staroverov, V. N.; Keith, T. A.; Kobayashi, R.; Normand, J.; Raghavachari, K.; Rendell, A. P.; Burant, J. C.; Iyengar, S. S.; Tomasi, J.; Cossi, M.; Millam, J. M.; Klene, M.; Adamo, C.; Cammi, R.; Ochterski, J. W.; Martin, R. L.; Morokuma, K.; Farkas, O.; Foresman, J. B.; Fox, D. J. *Gaussian 16 Rev. C.01*, Wallingford, CT, **2016**.
2. Chai, J.-D.; Head-Gordon, M. Long-range Corrected Hybrid Density Functionals with Damped Atom–atom Dispersion Corrections. *Phys. Chem. Chem. Phys.* **2008**, *10*, 6615–6620
3. Weigend, F.; Ahlrichs, R., Balanced basis sets of split valence, triple zeta valence and quadruple zeta valence quality for H to Rn: Design and assessment of accuracy. *Phys. Chem. Chem. Phys.* **2005**, *7* (18), 3297-3305.
4. Scalmani, G.; Frisch, M. J., Continuous surface charge polarizable continuum models of solvation. I. General formalism. *Chem. Phys.* **2010,** *132* (11), 114110.
5. Bannwarth, C.; Ehlert, S.; Grimme, S., GFN2-xTB—An Accurate and Broadly Parametrized Self-Consistent Tight-Binding Quantum Chemical Method with Multipole Electrostatics and Density-Dependent Dispersion Contributions. *J. Chem. Theory Comput.* **2019,** *15* (3), 1652-1671.
6. Pracht, P.; Bohle, F.; Grimme, S., Automated exploration of the low-energy chemical space with fast quantum chemical methods. *Phys. Chem. Chem. Phys.* **2020,** *22* (14), 7169-7192.
7. Marenich, A. V.; Cramer, C. J.; Truhlar, D. G., Universal Solvation Model Based on Solute Electron Density and on a Continuum Model of the Solvent Defined by the Bulk Dielectric Constant and Atomic Surface Tensions. *J. Phys. Chem. B.* **2009,** *113* (18), 6378-6396.
8. Grimme, S., Supramolecular Binding Thermodynamics by Dispersion-Corrected Density Functional Theory. *Chem. Eur. J.* **2012,** *18* (32), 9955-9964.
9. Luchini, G.; Alegre-Requena, J.; Funes-Ardoiz, I.; Paton, R., GoodVibes: automated thermochemistry for heterogeneous computational chemistry data [version 1; peer review: 2 approved with reservations]. *F1000research.* **2020,** *9* (291).
10. Kelly, C. P.; Cramer, C. J.; Truhlar, D. G., Aqueous Solvation Free Energies of Ions and Ion−Water Clusters Based on an Accurate Value for the Absolute Aqueous Solvation Free Energy of the Proton. *J. Phys. Chem. B.* **2006,** *110* (32), 16066-16081.
11. Fifen, J. J.; Dhaouadi, Z.; Nsangou, M., Revision of the Thermodynamics of the Proton in Gas Phase. *J. Phys. Chem. A.* **2014,** *118* (46), 11090-11097.

12. Ohtani, I.; Kusumi, T.; Kashman, Y.; Kakisawa, H. High-Field FT NMR Application of Mosher's Method. The Absolute Configurations of Marine Terpenoids. *J. Am. Chem. Soc.* **1991**, *113* (11), 4092–4096.
13. Jones, C. G.; Martynowycz, M. W.; Hattne, J.; Fulton, T. J.; Stoltz, B. M.; Rodriguez, J. A.; Nelson, H. M.; Gonen, T. The cryoEM method MicroED as a powerful tool for small molecule structure determination. *ACS Cent. Sci.* **2018**, *4*, 1587−1592.
14. Kabsch, W. *XDS*. *Acta Crystallogr. D Biol. Crystallogr.* **2010**, *66* (2), 125–132.
15. Kabsch, W. *XDS*. *Acta Crystallogr. D Biol. Crystallogr.* **2010**, *66* (2), 133-144.
16. Hattne, J.; Reyes, F. E.; Nannenga, B. L.; Shi, D.; de la Cruz, M. J.; Leslie, A. G. W.; Gonen, T. MicroED Data Collection and Processing. *Acta Crystallogr. A Found. Adv.* **2015**, *71* (4), 353–360.
17. Sheldrick, G. M. A Short History of*SHELX*. *Acta Crystallogr. A* **2008**, *64* (1), 112–122.
18. Sheldrick, G. M. *SHELXT*– Integrated Space-Group and Crystal-Structure Determination. *Acta Crystallogr. A Found. Adv.* **2015**, *71* (1), 3–8.
19. Hübschle, C. B.; Sheldrick, G. M.; Dittrich, B. *ShelXle*: A Qt Graphical User Interface for*SHELXL*. *J. Appl. Crystallogr.* **2011**, *44* (6), 1281–1284.
20. Tanaka, T.; Nakashima, T.; Ueda, T.; Tomii, K.; Kouno, I. Facile Discrimination of Aldose Enantiomers by Reversed-Phase HPLC. *Chem. Pharm. Bull. (Tokyo)* **2007**, *55* (6), 899–901.
21. Bruhn T, Schaumloffel A, Hemberger Y, Pescitelli G, SpecDis, Version 1.71. Berlin, Germany, **2017**, http:/specdis-software.jimdo.com.
22. Liang X, Miao F, Song Y, Liu X, Ji N. Citrinovirin with a New Norditerpene Skeleton from the Marine Algicolous Fungus Trichoderma Citrinoviride. *Bioorg. Med. Chem. Lett.* **2016**, *26* (20), 5029–5031.
